# Nonpolitical Images Evoke Neural Predictors of Political Ideology

**DOI:** 10.1016/j.cub.2014.09.050

**Published:** 2014-11-17

**Authors:** Woo-Young Ahn, Kenneth T. Kishida, Xiaosi Gu, Terry Lohrenz, Ann Harvey, John R. Alford, Kevin B. Smith, Gideon Yaffe, John R. Hibbing, Peter Dayan, P. Read Montague

**Affiliations:** 1 Virginia Tech Carilion Research Institute, Virginia Tech, Roanoke, VA 24016, USA; 2 Computational Psychiatry Unit, Virginia Tech, Roanoke, VA 24016, USA; 3 Wellcome Trust Centre for Neuroimaging, University College London, 12 Queen Square, London WC1N 3BG, UK; 4 Department of Political Science, Rice University, Houston, TX 77251-1892, USA; 5 Department of Political Science, University of Nebraska-Lincoln, Lincoln, NE 68588-0328, USA; 6 Yale Law School, Yale University, New Haven, CT 06511, USA; 7 Gatsby Computational Neuroscience Unit, University College London, London WC1N 3AR, UK; 8 Department of Physics, Virginia Tech, Blacksburg, VA 24061, USA

## Abstract

Political ideologies summarize dimensions of life that define how a person organizes their public and private behavior, including their attitudes associated with sex, family, education, and personal autonomy [1, 2]. Despite the abstract nature of such sensibilities, fundamental features of political ideology have been found to be deeply connected to basic biological mechanisms [3, 4, 5, 6, 7] that may serve to defend against environmental challenges like contamination and physical threat [8, 9, 10, 11, 12]. These results invite the provocative claim that neural responses to nonpolitical stimuli (like contaminated food or physical threats) should be highly predictive of abstract political opinions (like attitudes toward gun control and abortion) [13]. We applied a machine-learning method to fMRI data to test the hypotheses that brain responses to emotionally evocative images predict individual scores on a standard political ideology assay. Disgusting images, especially those related to animal-reminder disgust (e.g., mutilated body), generate neural responses that are highly predictive of political orientation even though these neural predictors do not agree with participants’ conscious rating of the stimuli. Images from other affective categories do not support such predictions. Remarkably, brain responses to a single disgusting stimulus were sufficient to make accurate predictions about an individual subject’s political ideology. These results provide strong support for the idea that fundamental neural processing differences that emerge under the challenge of emotionally evocative stimuli may serve to structure political beliefs in ways formerly unappreciated.

## Highlights

• Literature suggests negativity bias might underlie variations in political views
• fMRI responses to disgusting images accurately predict political orientation
• Self-reports about affective images are not predictive of their political views
• Single-stimulus data can reliably classify conservatives from liberals

## Teaser

Ahn et al. show that fMRI responses to disgusting images accurately predict political orientation. The effect is strong enough to elicit good classification of conservatives from liberals from single-stimulus data, suggesting that emotional processes play a much larger role in structuring our abstract political beliefs than we currently believe.

## Results

**Figure 1:**
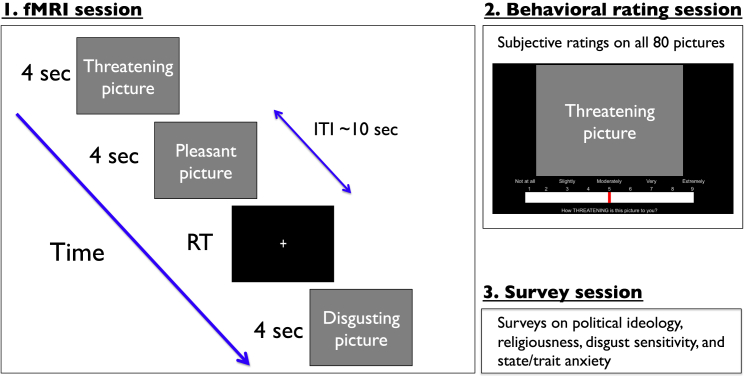
Time Course of the Experiment Each subject first participated in an affective picture-viewing task in the fMRI scanner, during which they viewed 80 color pictures (20 disgusting, 20 threatening, 20 pleasant, and 20 neutral pictures). Occasionally, a fixation cross appeared on the screen, and participants were asked to press a button as soon as they saw the cross. Each picture was presented for 4 s, and the fixation cross was presented until participants pressed a button. The mean intertrial interval (ITI) was 10 s. Next, participants completed a behavioral rating session and several computerized surveys (see the Supplemental Experimental Procedures). See also Figure S1.

We carried out a passive picture-viewing experiment to test the hypothesis that nonpolitical but affectively evocative images elicit brain responses that predict political ideology as assessed by a standard political ideology measure. Healthy volunteers (n = 83) were instructed to look at presented pictures while lying in the scanner, and, to control for attentiveness, we instructed them to press a button when a fixation cross appeared on the screen (Figure 1). Images were sampled from the International Affective Pictures database [14] and included disgusting, threatening, pleasant, and neutral images (see Appendix S1 available online). Each emotional condition had two subconditions (see the Supplemental Experimental Procedures). After the fMRI session, participants completed a behavioral rating session in which they rated all pictures they had seen in the scanner (using a nine-point Likert scale) as disgusting, threatening, or pleasant. Lastly, participants filled out computer-based questionnaires assessing their political attitudes, disgust sensitivity, and state/trait anxiety level. See the Supplemental Experimental Procedures for details of the behavioral rating and survey sessions.

**Figure 2:**
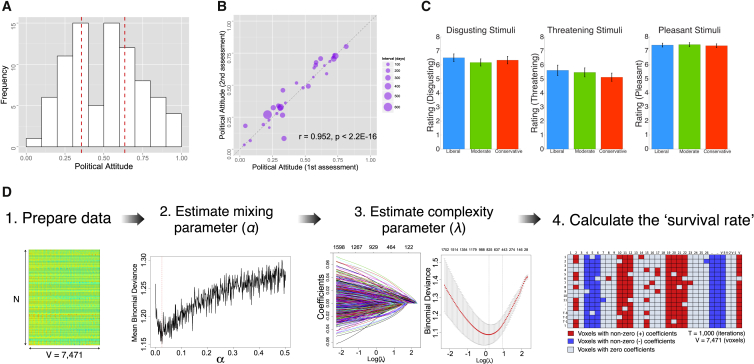
Behavioral Results and an Illustration of Workflow for Penalized Regression Analysis (A) Distribution of political attitudes (orientation). Political attitudes are scaled from 0 (extremely liberal) to 1 (extremely conservative) (mean = 0.500, SD = 0.225). We divided participants (n = 83) into three groups (liberal [n = 28], moderate [n = 27], and conservative [n = 28]) based on their political attitudes. Red dotted lines indicate tertiles (33.3% and 66.6%). (B) Test-retest reliability of political attitudes. The Pearson correlation coefficient is 0.952, p < 2.2 × 10^−16^, and the robust correlation coefficient is 0.986, p < 2.0 × 10^−16^. (C) Subjective ratings of emotional pictures for each group. Error bars indicate ±1 SE. (D) Schematic illustration of workflow for a machine-learning (penalized-regression) model. A 10-fold cross-validation is used to estimate two tuning parameters of the elastic net model. The survival rate was projected back into the brain space (see the Supplemental Experimental Procedures and Figure S3A). See also Figure S3.

Political ideology was summed from several survey items (Appendix S2), including ideological position, partisan affiliation, and policy preferences (e.g., gun control and immigration, presented in the well-known Wilson-Patterson format [15]). Survey items on political ideology were normalized continuously from 0 (extremely liberal) to 1 (extremely conservative) (see the Supplemental Experimental Procedures). Figure 2A shows its distribution across all participants. Political attitudes and interest did not show a significant linear relationship [r(81) = −0.148, p = 0.182], but instead showed a U-shaped curve (Figure S1A), indicating that greater political interest is associated with polarized political attitudes. When tested on a subset of participants, our measure of political attitudes shows excellent test-retest reliability (test-retest Pearson correlation coefficient = 0.952; Figure 2B). To focus our analyses on polarized political groups, we divided participants into three groups based on their political ideology scores (Table S1): liberal (n = 28), moderate (n = 27), and conservative (n = 28).

As seen in Figure 2C, groups did not significantly differ in subjective ratings of disgusting, threatening, or pleasant pictures (also see Table S2). Also, there were no significant group differences on self-report measures except that the conservative group had marginally higher disgust sensitivity than the liberal group [t(54) = 1.711, p = 0.093; Figure S1B and Table S1]. Note that emotional states can be implicit or nonconscious under some conditions [16]. Self-report measures may fail to detect some individual differences in disgust sensitivity [17].

Having characterized liberal and conservative groups behaviorally and confirmed blood-oxygen-level dependent (BOLD) responses to affective pictures (Figure S2 and Table S3), we used a machine-learning approach to predict individual differences in political orientation from the patterns of whole-brain BOLD responses. Specifically, we applied a penalized regression method called the elastic net [18] to our fMRI data (Figures 2D and S3 and the Supplemental Experimental Procedures). The elastic net algorithm offers several advantages for fMRI data, including automatic variable selection (i.e., regression coefficients of unimportant variables [voxels] shrink to zero) and model regularization, which increases the interpretability of the findings. The elastic net also enjoys a grouping effect, which clusters highly correlated predictors into a set of groups. The grouping effect is useful for fMRI data because they contain many correlated predictors (voxels) due to its inherent nature (i.e., a brain region may consist of many voxels) and spatial smoothing, which is a commonly used preprocessing step. Previous studies demonstrated that the elastic net performs better than least absolute shrinkage and selection operator (LASSO), especially when the number of predictors is much higher than the number of observations [18]. The elastic net is beginning to be applied to fMRI data [19, 20] and appears to be a promising tool for developing predictive models from neuroimaging (and other types of) data. Using the elastic net algorithm (penalized logistic regression analysis) and contrast maps ([disgusting > neutral], [threatening > neutral], or [pleasant > neutral]), we probed brain regions critical for cross-validated classification accuracy (liberal versus conservative groups; see the Supplemental Experimental Procedures).

**Figure 3:**
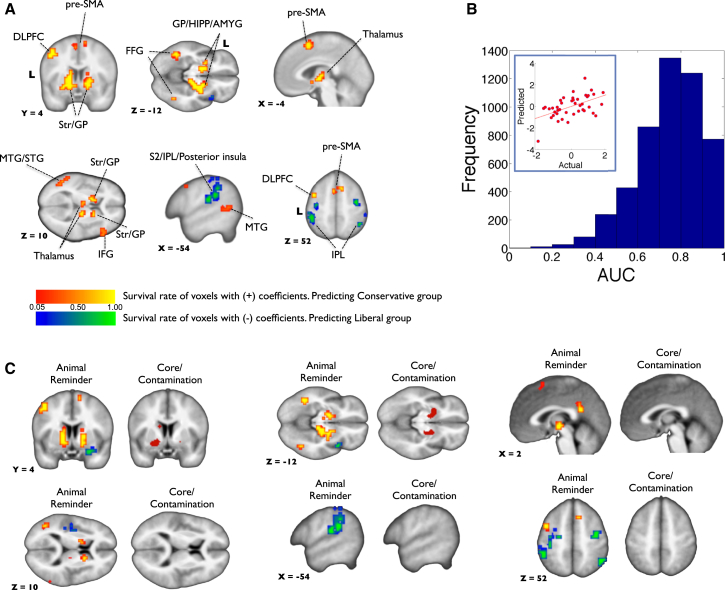
Multivariate Patterns of Brain Activity that Predict Political Ideology (A) Voxels predicting conservative (red-to-yellow) or liberal (blue-to-green) group membership from penalized logistic regression analysis (cluster size, k ≥ 10). Survival rate is closely related to voxel (regression) weights (see Figure S3B). DLPFC, dorsolateral prefrontal cortex; pre-SMA, presupplementary motor area; Str, striatum; GP, globus pallidus; HIPP, hippocampus; AMYG, amygdala; MTG/STG, middle/superior temporal gyrus; IFG, inferior frontal gyrus; S2, secondary somatosensory cortex; IPL, inferior parietal lobule; and FFG, fusiform gyrus. The color scale denotes the survival rate. (B) Distribution of cross-validated area under the curve (AUC). We ran 1,000 iterations of 5-fold cross-validation procedure. For each iteration, we first found the λ that minimized the out-of-sample binomial deviance of four folds (80% of the data). Then, for each of the five folds, we computed the area under the receiver-operating characteristic (ROC) curve using predictions from the model fit to the remaining data using the minimum λ. This resulted in the 5,000 (1,000 iterations × 5 AUCs per iteration) AUC calculations plotted in the histogram (mean = 0.757, median = 0.771, mode = 0.833, SD = 0.150). The inset in the top-left corner shows out-of-sample prediction performance on the half of the data (test set) when the model is trained on the other half of the data (training set) for penalized linear regression. The x and y axes show the *Z* scores of actual political attitudes and predicted political attitudes from BOLD signals, respectively. Pearson correlation coefficient = 0.52, p = 0.0004; robust correlation coefficient = 0.44, p = 0.0024. See the Supplemental Experimental Procedures for complete details. (C) Voxels predicting conservative or liberal group membership from each subcondition of disgust (i.e., using contrast maps of [animal-reminder disgust > neutral] or [core/contamination disgust > neutral]; see the Supplemental Experimental Procedures for the details of subconditions). The voxel survival criterion is the same as that for (A). See also Figure S2.

Figure 3A shows a network of brain regions predicting conservative and liberal group membership revealed by the machine-learning method with the [disgusting > neutral] contrast. Separate tests for the out-of-sample performance confirmed the robustness of the findings (Figure 3B and the Supplemental Experimental Procedures). No voxel survived cross-validations on other contrasts. Red-to-yellow and blue-to-green regions indicate voxels predicting conservative and liberal groups, respectively. As seen in Figure 3A, conservative group membership was predicted by increases in the basal ganglia (peak MNI = [16, 8, −8], k = 234)/thalamus (peak MNI = [20, −18, 6])/periaqueductal gray (PAG; peak MNI = [10, −24, −12]/hippocampus (peak MNI = [−14, −4, −14])/amygdala (peak MNI = [−18, −4, −14]), dorsolateral prefrontal cortex (DLPFC; peak MNI = [−44, 4, 52], k = 26), middle/superior temporal gyrus (MTG/STG; peak MNI = [−60, −44, 6], k = 33), presupplementary motor area (pre-SMA; peak MNI = [−4, 8, 56], k = 56), fusiform gyrus (FFG; peak MNI = [−42, −52, −10], k = 24 in the left side and [42, −60, −10], k = 16 in the right side), and inferior frontal gyrus (IFG; peak MNI = [52, 28, 4], k = 15). The increase in the secondary somatosensory cortex (S2)/posterior insula (peak MNI = [−40, −26, 19])/inferior parietal lobule (IPL; peak MNI = [−48, −40, 36], k = 125 in the left side and [48, −52, 54], k = 17 in the right side), frontal insula (MNI = [40, 16, −12], k = 19), and precentral gyrus (peak MNI = [−38, −12, 50], k = 25 in the left sid and [40, −12, 52], k = 13 in the right side) predicted liberal group membership. Note that the group differences using the traditional general linear modeling (GLM) revealed similar findings with some differences (Figure S2D, Table S4, and the Supplemental Experimental Procedures). The mean area under the curve (AUC) of the receiver-operating characteristic (ROC) curve was 0.981 (SD = 0.043). See the Supplemental Experimental Procedures and Figure S4 for more details and additional results using penalized linear regression across all participants. When we examined the prediction accuracy of each disgust subcondition (core/contamination and animal reminder), only animal-reminder disgust (e.g., mutilated body) was a strong predictor of political attitudes (Figure 3C; mean AUC = 0.998, SD = 0.003 for animal reminder; mean AUC = 0.548, SD = 0.125 for core/contamination).

Recent work suggests that BOLD time-series data from a single stimulus can categorically differentiate healthy individuals from those diagnosed with autism spectrum disorder (unpublished data). Lu et al. applied a machine-learning approach to time-series data from a specific region of interest and demonstrated that single-stimulus brain responses to a specific kind of stimulus could be used to make accurate categorical predictions of disorder status. We tested the hypothesis that a single-stimulus measurement combined with a machine-learning approach may contain enough information to predict liberal and conservative group membership per individual participant. Following Lu et al., we extracted the entire BOLD time-series response to the first disgusting picture. Time-series data every 2 s were spatially averaged within each of two types of patterns shown in Figure 3A: (+) voxels (red-to-yellow regions predicting conservative group) and (−) voxels (blue-to-green regions predicting liberal group) (see the Supplemental Experimental Procedures).

**Figure 4:**
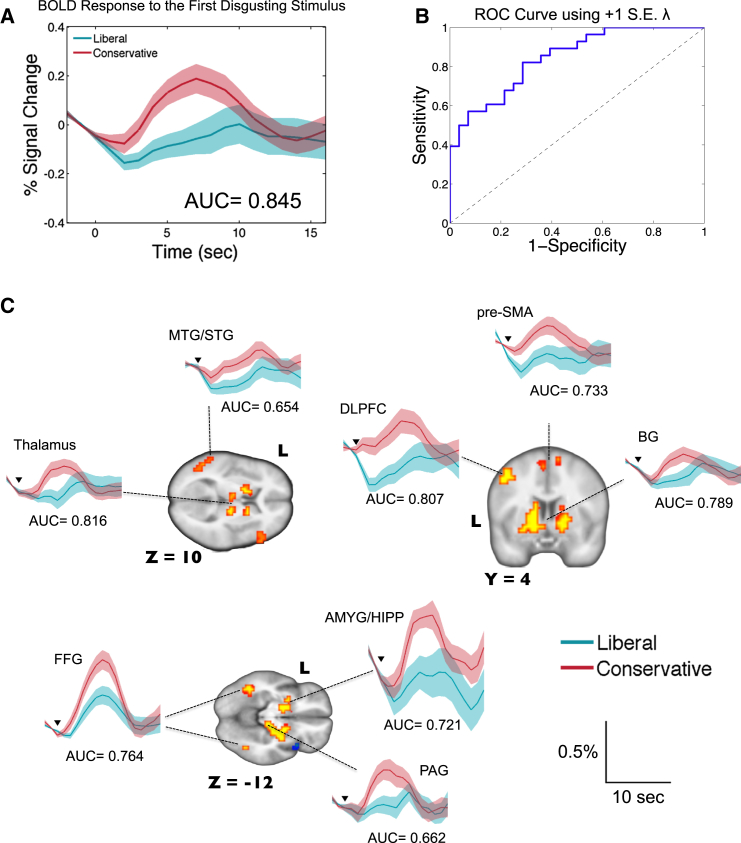
Single Presentation of a Disgusting Stimulus Is Sufficient to Make Accurate Predictions of Individual’s Political Orientation (A) Hemodynamic response to the first disgusting stimulus for the liberal and conservative groups, extracted from the red-to-yellow voxels in Figure 3A. Shaded regions indicate ±1 SE. Time-series data were linearly interpolated every 1 s for display purposes. “AUC” indicates the mean AUC of ROC curves over 1,000 iterations. (B) A representative ROC curve. (C) Hemodynamic response to the first disgusting stimulus, extracted from each predictive region, as well as the mean AUCs of the corresponding ROC curves. The x axis is time since stimulus presentation (s) and the y axis is the percent signal change (percentage). Black inverse triangles indicate the stimulus onset, the bottom of which is at 0.05% signal change. DLPFC, dorsolateral prefrontal cortex; pre-SMA, presupplementary motor area; BG, basal ganglia; AMYG/HIPP, amygdala/hippocampus; MTG/STG, middle/superior temporal gyrus; FFG, fusiform gyrus; and PAG, periaqueductal gray. See also Figure S4.

As seen in Figure 4A, the single-stimulus presentation of the disgusting pictures reliably differentiated the conservative and liberal groups in the (+) voxels. The hemodynamic response of the conservative group had a steeper slope and a higher peak than that of the liberal group. The mean AUC of the ROC curve using the single-stimulus presentation was 0.845 (SD = 0.009; Figure 4B). When we used each region of interest within the (+) voxels for the same analysis (Figure 4C), the thalamus (mean AUC = 0.816, SD = 0.023) and the DLPFC (mean AUC = 0.807, SD = 0.018) were the strongest predictive regions, followed by the basal ganglia (mean AUC = 0.789, SD = 0.005), FFG (mean AUC = 0.764, SD = 0.047), pre-SMA (mean AUC = 0.733, SD = 0.044), amygdala/hippocampus (mean AUC = 0.721, SD = 0.079), PAG (mean AUC = 0.662, SD = 0.100), and MTG/STG (mean AUC = 0.654, SD = 0.105). While increase in the (−) voxels predicted liberal group membership with full data, none of the BOLD time-series data from the (−) voxels survived using the single-stimulus analysis.

## Discussion

Neuroscience has started to provide rich information about the neurophysiological processes underlying political behavior. Our results have important implications for the links between biology, emotions, political ideology, and human nature more fundamentally. While previous studies using skin conductance response [9, 10, 11], neuroimaging [21, 22, 23, 24], and questionnaire [25, 26] measures suggested the role of emotions in political attitudes, to our knowledge, this is the first fMRI study revealing multivariate patterns of brain activity that differ between liberals and conservatives during emotional processing of sensory stimuli. Accumulating evidence suggests that cognition and emotion are deeply intertwined [27], and a view of segregating cognition and emotion is becoming obsolete [28]. People tend to think that their political views are purely cognitive (i.e., rational). However, our results further support the notion that emotional processes are tightly coupled to complex and high-dimensional human belief systems [13], and such emotional processes might play a much larger role than we currently believe, possibly outside our awareness of its influence [29]. Despite growing evidence from various fields, including genetics, cognitive neuroscience, and psychology, many political scientists remain skeptical of research connecting biological factors with political ideology, arguing variously that biology is irrelevant to central political questions [30], that the theoretical basis for expecting biology to be relevant is weak and murky [31], that acknowledging a role for biology is reductionist [32], and that recognizing the relevance of biology to human beliefs and behaviors is potentially dangerous [33]. We hope some of this skepticism can be alleviated from our demonstration that fMRI data, even from a single stimulus, can serve as a strong predictor of political ideology.

Several groups have suggested that people are born with certain dispositions and traits that influence the formation of their political beliefs [3, 4]. Also, several studies have shown that life history (e.g., [34]) and traumatic experiences [35] can affect political views. Our results are consistent with the idea that political beliefs are connected to neurobiological composition. But both genetics and life history play an important role in establishing both connections between neuroanatomical regions and the propensity for these regions to respond to environmental stimuli. We have not isolated the distinct roles played by genetics and life history in the development of the brain responses that we measured.

A wide range of brain regions contributed to the prediction of political ideology (Figure 3A), including those known from past work to be involved in the processing and interoception of disgust and other stimuli with negative affective valence, but also those involved in more basic aspects of attentive sensory processing: we found regions known to be involved in disgust recognition [17, 36, 37, 38] (e.g., insula, basal ganglia, and amygdala), perception of bodily signals [39] (e.g., insula), the experience of physical/social pain [40] or observing others in pain [41] (e.g., S2, insula, PAG, and thalamus), and emotion regulation [42] (e.g., DLPFC, insula, amygdala, and pre-SMA), along with regions involved in information integration [43] (e.g., thalamus and amygdala), attention [43, 44] (e.g., amygdala, IPL, FFG, STG/MTG), memory retrieval [44, 45] (e.g., hippocampus, amygdala, and IPL), and also inhibitory control [46] (e.g., IFG, DLPFC, and pre-SMA), perhaps to suppress innate responses. Although our results suggest that disgusting pictures evoke very different emotional processing in conservatives and liberals, it will take a range of targeted studies in the future to tease apart the separate contribution of each brain circuit.

We proposed that conservatives, compared to liberals, have greater negativity bias [13], which includes both disgusting and threatening conditions in our study. Our finding that only disgusting pictures, especially in the animal-reminder category, differentiate conservatives from liberals might be indicative of a primacy for disgust in the pantheon of human aversions, but it is also possible that this result is due to the fact that, compared to threat, disgust is much easier to evoke with visual images on a computer screen.

Lastly, this study raises several important but unaddressed questions. First, while political ideology has effects on many forms of behavior (including, but not limited to, voting behavior), it is unknown whether it does so thanks to the neural differences in affective processing that we measured. Second, and relatedly, it is important also to know how individual differences in the capacity to regulate emotion [26], and the neural bases of that capacity, are related to political ideology. A third set of questions concerns the bearing of the present study on the development of biological measures of political ideology. While it is of use in a variety of settings to measure political ideology (political polls, for instance, typically include some measurement of it), it remains an open question whether biological measures could become more accurate, or more useful, than the tools (such as self-report measures) currently employed. Determining the answer to that question would require answering a host of others: How would a machine-learning model based on data collected in one region (e.g., New York) support predictions of people’s political attitudes in another region (e.g., Texas)? How fine-grained are the categories of affective response that are tied to political ideology? Although our results show greater differentiation in political ideology in cases of animal-reminder disgust than core/contamination disgust, what are the links between political ideology and other forms of disgust, such as moral disgust? The more we learn about the sensitivity of political ideology to subtle differences in affective response and their neural bases, the more we will know about the feasibility of useful and portable tools for ideology’s biological measurement. This would then raise a further and difficult ethical question about the circumstances, if any, in which it is appropriate to use such tools. And, finally, the present study raises important questions about the possibility of, and obstacles to, understanding and cooperation across divides in political ideology. Would the recognition that those with different political beliefs from our own also exhibit different disgust responses from our own help us or hinder us in our ability to embrace them as coequals in democratic governance? Future work will be necessary to answer these important questions.

## Experimental Procedures

### Participants

Eighty-three healthy individuals (males/females = 41/42; age = 18–62; mean [SD] = 29.0 [11.3] years) in Roanoke and Blacksburg, VA, area were recruited from a large database maintained by the Human Neuroimaging Laboratory between September 2012 and September 2013. See the Supplemental Experimental Procedures for inclusion/exclusion criteria for participants and demographic data.

### fMRI Task

Participants were informed that they would complete a simple visual perception task. They were told to simply look at emotional pictures when they were presented but to press a button when they saw a fixation cross. Figure 1 depicts the time course of the fMRI experiment. It is a passive picture-viewing task presenting a total of 20 disgusting, 20 threatening, 20 pleasant, and 20 neutral pictures, the order of which was randomized for each participant. All the pictures were taken from the International Affective Picture System (IAPS) [14]. See Appendix S1 for IAPS picture numbers, description, and valence/arousal ratings of all pictures. Table S2 summarizes the mean IAPS valence and arousal ratings in each emotion condition. Each picture was presented for 4 s. Ten button-press (fixation-cross) trials were pseudorandomly mixed with emotional pictures to help participants stay fully awake and pay attention to visual stimuli. The fixation cross stayed on the screen until a button was pressed. Each trial was separated by a Poisson-distributed variable interval (mean = 10 s, SD = 10 s, minimum = 6 s, maximum = 17 s). The experiment took approximately 20 min in total. NEMO (http://labs.vtc.vt.edu/hnl/nemo/index.html) was used for stimuli presentation and behavioral response collection.

### MRI Data Acquisition and Analysis

The anatomical and functional imaging sessions were conducted on a 3.0 tesla Siemens Magnetom Trio scanner at VTCRI. We used SPM8 (http://www.fil.ion.ucl.ac.uk/spm/software/spm8/) for preprocessing and standard GLM fMRI analyses. For the elastic net analysis, we used the *glmnet* package for MATLAB (http://web.stanford.edu/∼hastie/glmnet_matlab/) and R [47]. See the Supplemental Experimental Procedures for complete details.

## Author Contributions

W.-Y.A., X.G., T.L., A.H., J.R.A., K.B.S., J.R.H., and P.R.M. conceived and designed the experiments; W.-Y.A. performed the research; W.-Y.A. and T.L. analyzed the data; P.R.M. supervised the project; and all authors wrote the paper.

## Supplemental Information

Document S1. Supplemental Experimental Procedures, Figures S1–S4, Tables S1–S4, and Appendices S1 and S2

Document S2. Article plus Supplemental Information
