# Supplementary material for: Nonpolitical Images Evoke Neural Predictors of Political Ideology
Source: Curr Biol. 2014 Nov 17;24(22):2693–9. doi: 10.1016/j.cub.2014.09.050 (PMC4245707; doi:10.1016/j.cub.2014.09.050)
Supplement: Document S1. Supplemental Experimental Procedures, Figures S1–S4, Tables S1–S4, and Appendices S1 and S2 [file mmc1.pdf]

Current Biology, Volume *24*
Supplemental Information

Nonpolitical Images Evoke Neural Predictors of Political Ideology

Woo-Young Ahn, Kenneth T. Kishida, Xiaosi Gu, Terry Lohrenz, Ann Harvey, John R. Alford, Kevin B. Smith, Gideon Yaffe, John R. Hibbing, Peter Dayan, and P. Read Montague

Figure S1

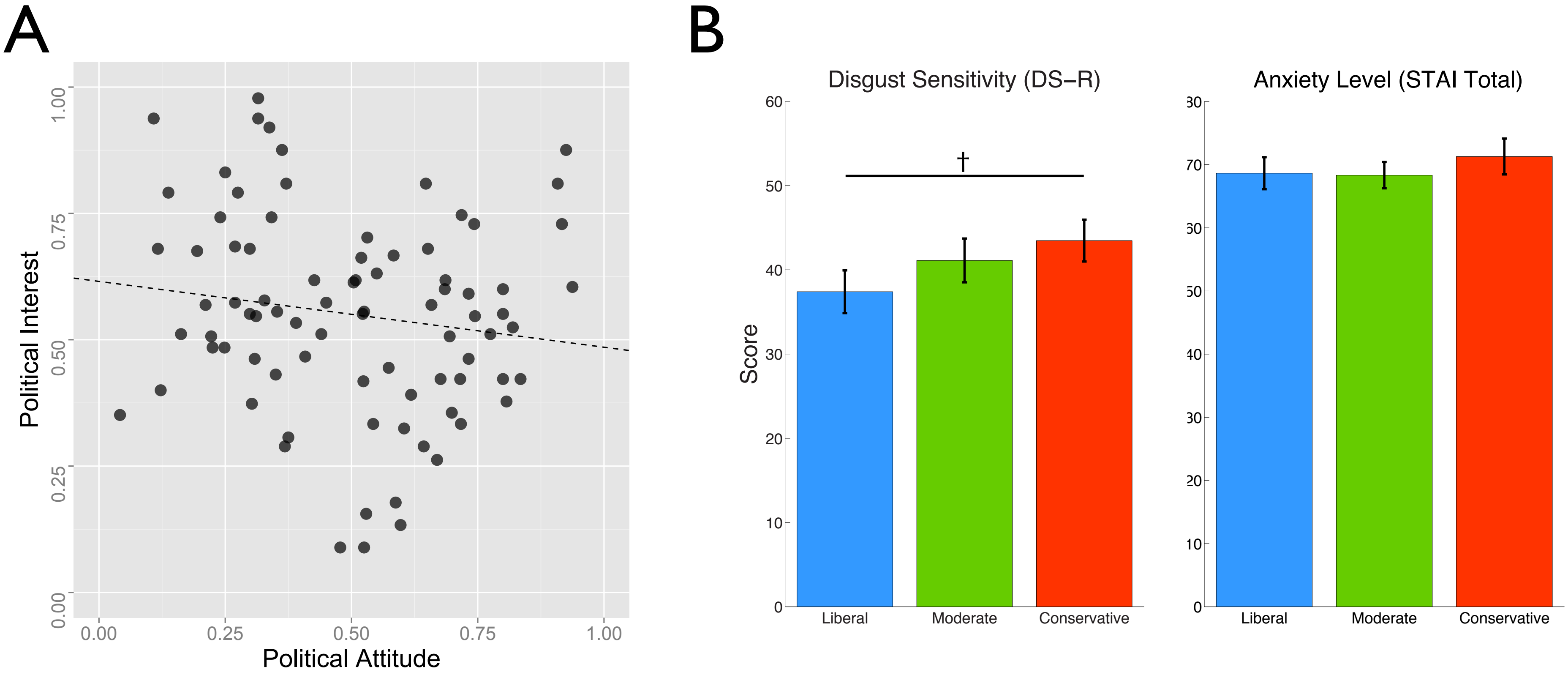
A
Political Interest
Political Attitude
0.00
0.25
0.50
0.75
1.00
B
Disgust Sensitivity (DS−R)
Anxiety Level (STAI Total)
Score
†
Liberal
Moderate
Conservative
Liberal
Moderate
Conservative

Figure S2

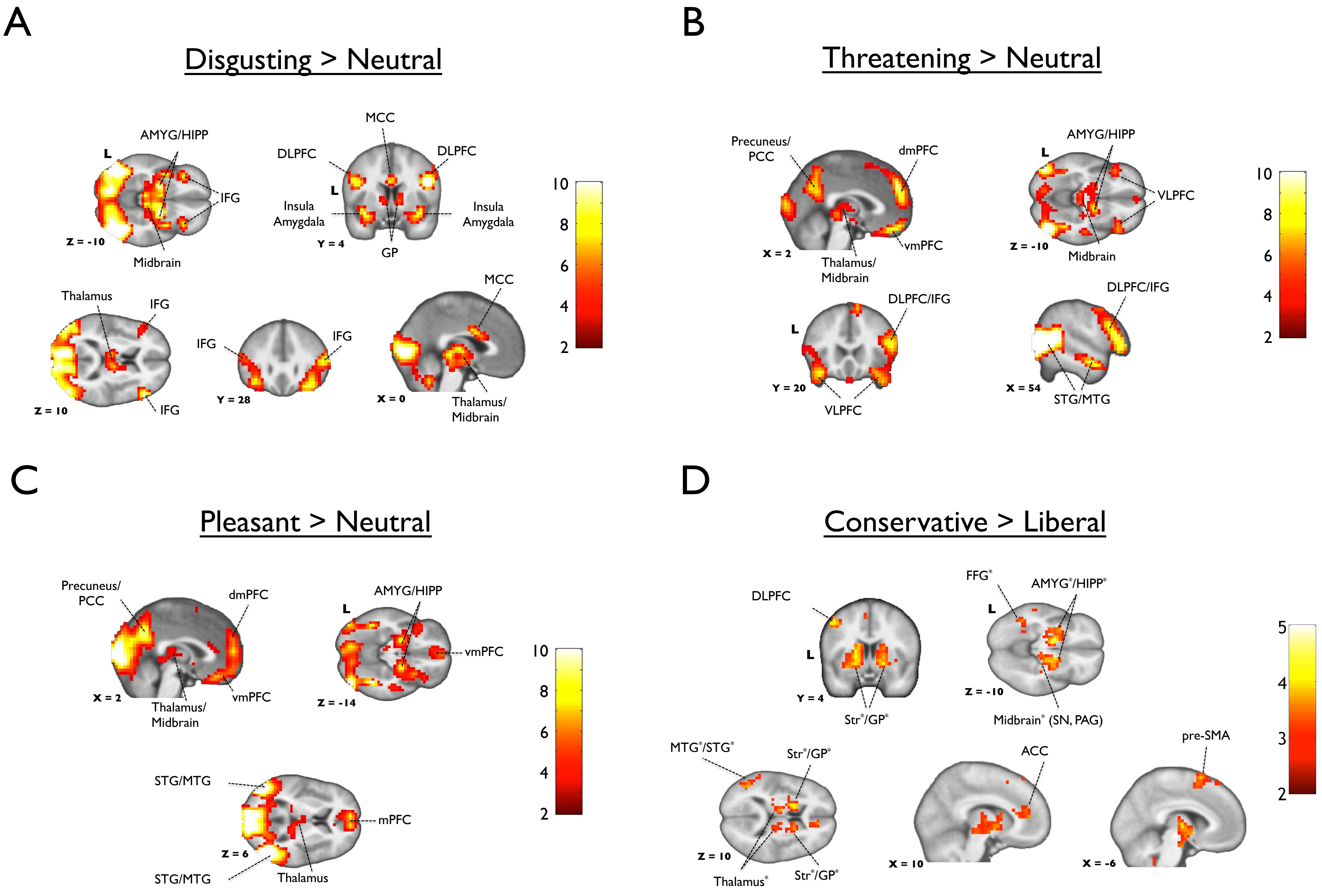
A
Disgusting > Neutral
AMYG/HIPP
L
IFG
Z = -10
Midbrain
MCC
DLPFC
DLPFC
L
Insula
Amygdala
Insula
Amygdala
Y = 4
GP
Thalamus
IFG
IFG
Z = 10
IFG
IFG
Y = 28
MCC
X = 0
Thalamus/
Midbrain
10
8
6
4
2
B
Threatening > Neutral
Precuneus/
PCC
dmPFC
X = 2
Thalamus/
Midbrain
vmPFC
AMYG/HIPP
L
VLPFC
Z = -10
Midbrain
10
8
6
4
2
DLPFC/IFG
L
Y = 20
VLPFC
DLPFC/IFG
X = 54
STG/MTG
C
Pleasant > Neutral
Precuneus/
PCC
dmPFC
X = 2
Thalamus/
Midbrain
vmPFC
AMYG/HIPP
L
vmPFC
Z = -14
STG/MTG
mPFC
Z = 6
STG/MTG
Thalamus
10
8
6
4
2
D
Conservative > Liberal
DLPFC
L
Y = 4
Str*/GP*
FFG*
AMYG*/HIPP*
L
Z = -10
Midbrain* (SN, PAG)
MTG*/STG*
Str*/GP*
Z = 10
Thalamus*
Str*/GP*
ACC
X = 10
pre-SMA
X = -6
5
4
3
2

Figure S3

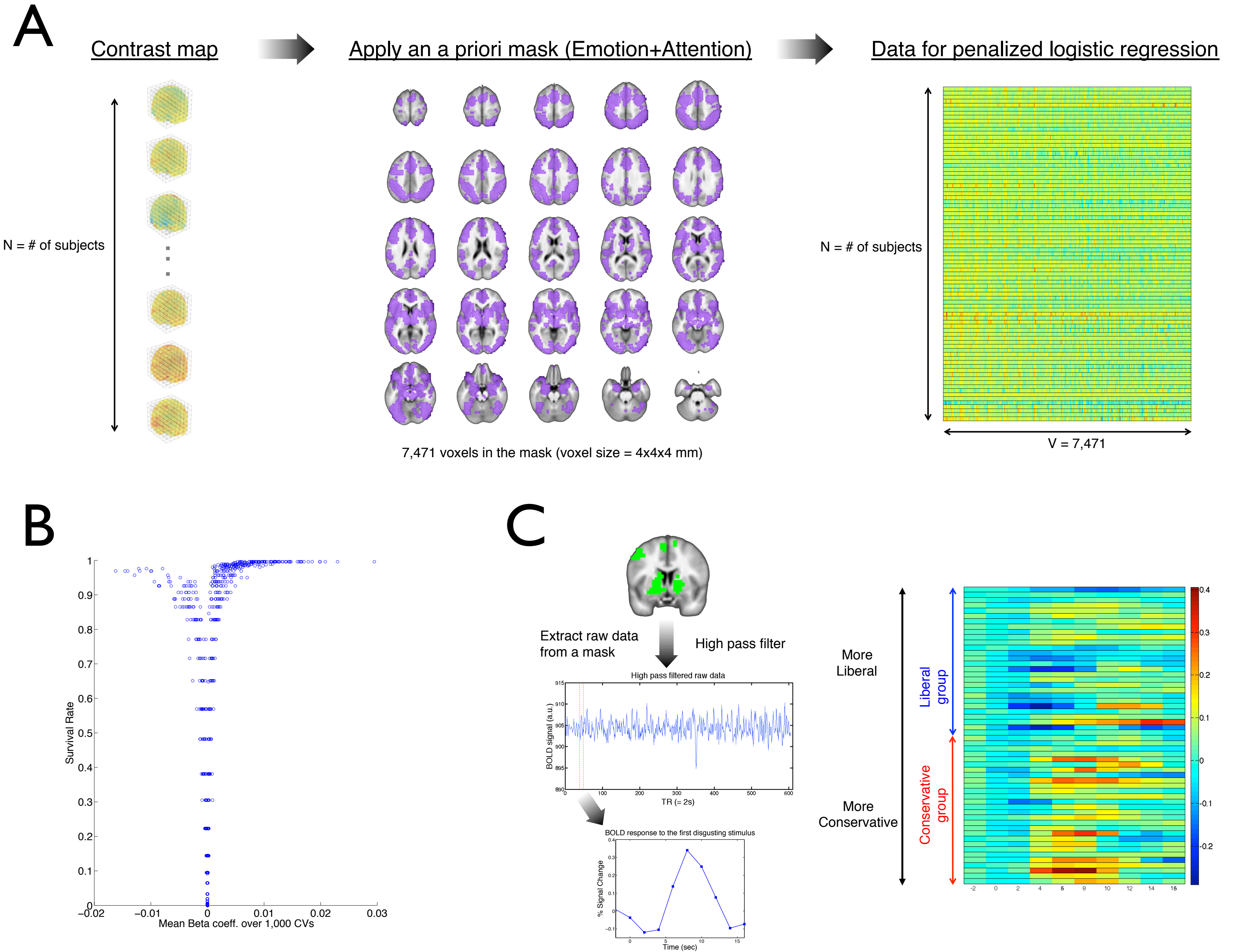
A
Contrast map
Apply an a priori mask (Emotion+Attention)
Data for penalized logistic regression
N = # of subjects
7,471 voxels in the mask (voxel size = 4x4x4 mm)
N = # of subjects
V = 7,471
B
Survival Rate
Mean Beta coeff. over 1,000 CVs
C
Extract raw data
from a mask
High pass filter
High pass filtered raw data
BOLD signal (a.u.)
TR (= 2s)
BOLD response to the first disgusting stimulus
% Signal Change
Time (sec)
More
Liberal
More
Conservative
Liberal
group
Conservative
group

Figure S4

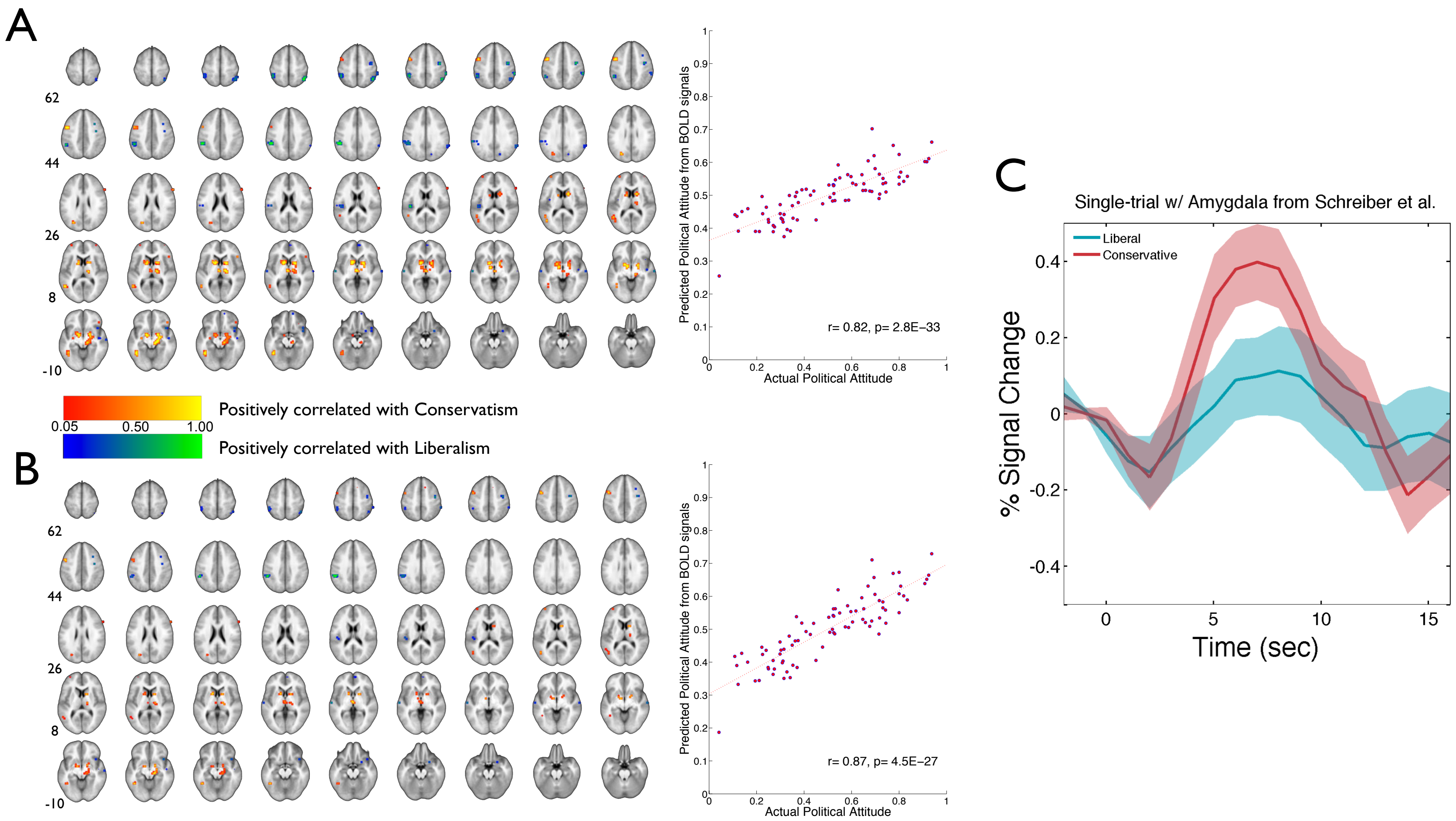
A
62
44
26
8
-10
Predicted Political Attitude from BOLD signals
Actual Political Attitude
r= 0.82, p= 2.8E−33
0.05
0.50
1.00
Positively correlated with Conservatism
Positively correlated with Liberalism
B
62
44
26
8
-10
Predicted Political Attitude from BOLD signals
Actual Political Attitude
r= 0.87, p= 4.5E−27
C
Single-trial w/ Amygdala from Schreiber et al.
Liberal
Conservative
% Signal Change
Time (sec)

Supplemental Figure Legends

Figure S1: Related to Figure 1. (A) The relationship between political attitudes and political interest. A dashed line is the linear regression fit ($r = -0.148$, $p = 0.182$) (B) Disgust Sensitivity (DS-R) and Anxiety Level (STAI Total) survey scores. The Liberal group reported marginally lower disgust sensitivity than the Conservative group. Error bars indicate ±1 S.E.

Figure S2: Related to Figure 3. (A, B, C) The contrasts of [Disgusting > Neutral], [Threatening > Neutral], and [Pleasant > Neutral] stimuli using the standard General Linear Model (GLM) analysis across all participants (n=83). All maps are at $p < 0.001$, whole-brain family-wise error (FWE) corrected at the cluster level. (D) The comparison of Conservative and Liberal groups using standard General Linear Model (GLM) analysis for the [Disgusting > Neutral] contrast. All maps are at $p < 0.005$ (uncorrected, cluster size > 10). Regions with * marks survived the FWE cluster-level correction. AMYG, amygdala; HIPP, hippocampus; IFG, inferior frontal gyrus; MCC, middle cingulate cortex; DLPFC, dorsolateral prefrontal cortex; GP, globus pallidus; PCC, posterior cingulate cortex; vmPFC, ventromedial prefrontal cortex; dmPFC, dorsomedial prefrontal cortex; VLPFC, ventrolateral prefrontal cortex; STG/MTG, superior/middle temporal gyrus; Str, striatum; SN, substantia nigra; PAG, periaqueductal gray; ACC, anterior cingulate cortex; pre-SMA, pre-supplementary motor area; FFG, fusiform gyrus. Color scale denotes t-scores.

Figure S3: Related to Figure 2. (A) Preparation of data for the full-data penalized regression analysis. First, prepare a map of the contrast of interest (e.g., [Disgust > Neutral]) for each participant. Second, apply an a priori mask generated from the Neurosynth website (neurosynth.org). We used the union of activations from “Emotion” and “Attention” terms (including both forward and reverse inference maps). Third, generate a matrix (number of subjects x number of voxels) for a penalized (logistic or linear) regression analysis. (B) An illustration of the relationship between survival rates and regression coefficients. The plot shows that higher survival rates are associated with greater magnitudes of beta coefficients in penalized logistic regression analysis. Each dot indicates each voxel within the mask (total number of voxels = 7,471). (C) Preparation of data for the single-stimulus fMRI (penalized logistic regression) analysis. (A) In each participant, extract the raw time series data that are spatially averaged within the red-to-yellow voxels (**Figure 3A**) or a region of interest, and then apply a high pass filter to the data, which are then linearly interpolated every 1s. Extract an epoch of [-2s, 16s] (indicated by red dashed lines) every 2s from the onset of the first disgusting picture (11 time points for each participant). (B) Generate a matrix (number of participants x number of time points) for the single-stimulus fMRI analysis. Data are sorted by political attitudes for display purposes. Labels on the x-axis indicate time since stimulus presentation (sec). Color scale denotes percent signal change.

Figure S4: Related to Figure 4. Voxels that are positively (red-to-yellow) or negatively (blue-to-green) correlated with political attitudes (using the [Disgusting > Neutral] contrast). We used penalized linear regression and all participants for generating the maps. The numbers on axial slices indicate MNI Z-axis coordinates. (A) Using α (mixing parameter) value optimized for penalized logistic regression ($\alpha = 0.026$). (B) Using α value optimized for penalized linear regression ($\alpha = 0.222$). Scatter plots on the right side indicate correlation plots between actual political attitudes and predicted political attitudes from BOLD data across all participants. Color scale denotes survival rate. (C) Hemodynamic response to the first disgusting stimulus, extracted from an independent amygdala ROI from [S1]. Shaded regions indicate ±1 S.E. See **Supplemental Experimental Procedure** for complete details.

Table S1. Demographic/survey data and IAPS ratings of the first disgusting stimuli for the Liberal, Moderate, and Conservative groups. Values indicate means and standard deviations (in parentheses) except for Sex. C/A = Core/Contamination versus Animal Reminder (disgust).

	Liberal (n=28)	Moderate (n=27)	Conservative (n=28)
Age	32.7 (15.5)	27.6 (9.2)	26.8 (11.2)
Sex (Male/Female)	17/11	13/14	11/17
Political orientation	0.25 (0.08)	0.50 (0.08)	0.75 (0.09)
Political interest	0.63 (0.18)	0.46 (0.22)	0.55 (0.16)
DS-R Total	37.39 (13.41)	41.11 (13.51)	43.46 (13.14)
STAI Total	68.64 (13.42)	68.33 (10.82)	71.29 (15.01)
IAPS valence rating of the first disgusting stimuli	2.30 (0.81)	2.17 (0.67)	2.22 (0.79)
IAPS arousal rating of the first disgusting stimuli	6.04 (1.20)	6.20 (0.93)	6.27 (0.76)
Onset of the first disgusting stimuli (sec)	30.96 (36.87)	45.33 (60.90)	50.57 (40.64)
Subcondition of the first disgusting stimuli (C/A)	14/14	13/14	12/16

Table S2. The mean and standard deviations (in parentheses) of IAPS valence and arousal ratings as well as behavioral ratings in each rating category.

	IAPS valence rating	IAPS arousal rating	Disgusting rating	Threatening rating	Pleasant rating
Disgusting stimuli	2.42 (0.82)	5.96 (0.99)	6.35 (1.39)	3.29 (1.90)	2.34 (0.62)
Threatening stimuli	2.79 (0.62)	6.28 (0.59)	3.15 (1.81)	5.40 (1.72)	2.84 (0.85)
Pleasant stimuli	7.87 (0.38)	4.62 (0.75)	1.08 (0.22)	1.11 (0.18)	7.42 (0.70)
Neutral stimuli	5.10 (0.39)	3.01 (0.72)	1.12 (0.29)	1.17 (0.35)	5.18 (0.34)

Table S3. Regions from the [Disgusting > Neutral], [Threatening > Neutral], and [Pleasant > Neutral] contrasts (Figure S2). Whole-brain family-wise error (FWE) corrected at the cluster level ($p < 0.05$). Height threshold, $t = 3.19$; extent thresholds = 44, 40, and 43 voxels (4x4x4 mm3). L: Left, R: Right.

Contrast	Region	Cluster size	Side	MNI coordinates x	y	z	Peak T-score
Disgusting > Neutral	Dorsolateral prefrontal cortex	3445	L	-46	4	26	8.46
	Dorsolateral prefrontal cortex		R	46	4	26	12.64
	Middle occipital gyrus		L	-50	-76	-2	14.35
	Middle occipital gyrus		R	50	-72	-6	13.22
	Fusiform gyrus		L	-42	-52	-18	13.31
	Fusiform gyrus		R	42	-52	-18	13.19
	Middle temporal gyrus		L	-46	-76	10	8.37
	Middle temporal gyrus		R	46	-76	10	9.05
	Supramarginal gyrus		L	-62	-28	34	7.92
	Amygdala	1701	L	-22	-8	-14	13.86
	Amygdala		R	22	-4	-14	12.12
	Hippocampus		L	-22	-32	-2	7.63
	Hippocampus		R	33	-32	-2	8.69
	Globus pallidus/caudate		L	-10	4	2	4.88
	Globus pallidus/caudate		R	10	4	2	5.47
	Anterior insula		L	-34	24	2	5.53
	Anterior insula		R	38	28	-2	5.87
	Posterior insula		L	-42	-4	-2	4.94
	Posterior insula		R	42	-4	-2	5.73
	Inferior frontal gyrus		L	-50	32	18	5.71
	Inferior frontal gyrus		R	50	32	10	9.34
	Thalamus		L	-6	-16	6	7.79
	Thalamus		R	6	-12	2	8.56
	Midbrain (substantia nigra/periaqueductal gray)		L	-6	-28	-6	8.46
	Midbrain (substantia nigra/periaqueductal gray)		R	6	-28	-6	8.57
	Middle cingulate cortex	59	L	-2	0	30	7.20
	Supramarginal gyrus	54	R	62	-24	34	6.23
	Middle temporal gyrus	1394	L	-46	-76	14	13.08
	Middle temporal gyrus		R	50	-68	2	19.41
	Superior temporal gyrus		R	54	-52	10	15.34
	Fusiform gyrus		L	-42	-48	-18	12.69
	Fusiform gyrus		R	42	-48	-18	16.12

Threatening > Neutral	Dorsolateral prefrontal cortex/Inferior frontal gyrus	1266	L	-46	24	6	4.90
	Dorsolateral prefrontal cortex/Inferior frontal gyrus		R	54	24	14	8.90
	Amygdala		L	-18	-8	-14	5.69
	Amygdala		R	22	-4	-14	7.25
	Hippocampus		L	-22	-28	-6	4.53
	Hippocampus		R	26	-24	-10	4.14
	Inferior frontal gyrus		L	-38	20	-18	7.42
	Inferior frontal gyrus		R	38	28	-14	7.89
	Midbrain (substantia nigra/periaqueductal gray)		L	-6	-28	-6	6.66
	Midbrain (substantia nigra/periaqueductal gray)		R	6	-28	-6	6.95
	Middle occipital gyrus	658	L	-46	-76	6	17.17
	Middle occipital gyrus		R	46	-76	2	15.09
	Dorsomedial prefrontal cortex	475	L	-2	48	34	9.52
	Precuneus	245	R	2	-60	30	7.15
	Ventromedial prefrontal cortex	110	R	2	40	-22	8.06
	Cerebellum	60	L	-18	-76	-38	6.24
Pleasant > Neutral	Amygdala	3854	L	-18	-8	-14	7.40
	Amygdala		R	22	-4	-14	8.15
	Hippocampus		L	-26	-24	-10	4.80
	Hippocampus		R	26	-28	-6	4.95
	Fusiform gyrus		L	-42	-52	-22	10.69
	Fusiform gyrus		R	42	-48	-18	13.09
	Middle occipital gyrus		L	-46	-80	6	15.83
	Middle occipital gyrus		R	50	-76	-2	16.81
	Middle/superior temporal gyrus		L	-46	-68	6	9.77
	Middle/superior temporal gyrus		R	50	-64	6	14.97
	Thalamus		L	-6	-12	6	3.63
	Precuneus		L	-2	-60	38	9.42
	Dorsomedial prefrontal cortex	582	L	-6	56	6	7.31
	Dorsomedial prefrontal cortex		R	6	56	10	8.03
	Ventromedial prefrontal cortex		R	2	36	-22	6.62
	Pre-supplmentary motor area	67	R	10	8	66	5.40
	Precentral gyrus	44	R	50	-4	50	6.21

Table S4. Conservative versus Liberal groups in the [Disgusting > Neutral] contrast (Figure S2D). Height threshold, t = 2.67; extent threshold = 10 voxels (4x4x4 mm^3). L: Left, R: Right. Activation with a * mark survives the whole-brain FWE correction at the cluster level ($p < 0.05$, $k \geq 109$).

Contrast	Region	Cluster size	Side	MNI coordinates			Peak T-score
				x	y	z	
Conservative > Liberal	Globus pallidus*	591	L	-14	-4	6	4.72
	Globus pallidus*		R	18	-8	-6	4.64
	Caudate*		L	-14	8	10	4.05
	Caudate*		R	18	8	14	3.70
	Putamen*		L	-18	8	10	4.31
	Putamen*		R	22	0	-2	3.12
	Amygdala/hippocampus*		L	-30	-4	-14	3.20
	Amygdala/hippocampus*		R	22	-8	-10	3.66
	Thalamus*		L	-10	-16	14	3.97
	Thalamus*		R	18	-12	2	4.17
	Middle/superior temporal gyrus*	163	L	-46	-68	14	3.99
	Fusiform gyrus*		L	-42	-52	-14	3.17
	Pre-supplementary motor area	79	L	-6	12	66	3.75
	Cerebellum	52	L	-22	-84	-42	3.79
	Cerebellum		R	22	-76	-42	4.18
	Anterior cingulate cortex	29	R	10	44	10	3.48
	Dorsolateral prefrontal cortex	26	L	-46	4	50	4.09
	Ventromedial prefrontal cortex	12	R	6	36	-22	3.03
	Inferior frontal gyrus	11	R	50	28	6	3.08
	Posterior cingulate cortex	10	L	-6	-48	30	3.11

Supplemental Experimental Procedures

Inclusion/exclusion criteria for participants and demographic data

Participants were required to be at least 18 years of age and to meet standard health and safety requirements for the MRI experiment. Additional twelve participants were excluded from all the analyses: 5 participants had more than 3mm of maximum movement; 2 participants had aberrant brain structure (e.g., extremely large ventricles); 1 participant fell asleep during the fMRI experiment and had extremely long reaction time (RT) for button-press trials; 2 participants showed no or little activity in the visual cortex for the disgust > neutral contrast in the first level analysis (threshold $p < 0.05$, $k = 10$, uncorrected); 2 participants had signal dropout. Participants gave informed consent in accordance with the Institutional Review Board at Virginia Tech Carilion Research Institute (VTCRI), VA, USA. After filling out a screening form and a written consent, participants were given written and verbal instructions on the task. Participants were divided into three groups based on their political ideology score. Neither age ($F(2,80)=2.331$, $p=0.104$) nor sex ($\chi^2(2)=2.597$, $p=0.273$) was significantly different across groups. When we tested just Liberal and Conservative groups, the Liberal group was marginally older than the Conservative group ($t(54)=1.857$, $p=0.069$). There were more males in the Liberal group compared to the Conservative group, but the group difference on age was not significant ($\chi^2(1)=1.786$, $p=0.181$).

Subconditions of emotional pictures used in the fMRI task

Each emotional condition, except the neutral condition, has two subconditions: 9 core/contamination (e.g., a dirty toilet) and 11 animal reminder (e.g., mutilated body) pictures in the disgusting condition, 10 actual threat (threatening objects aimed at another individual) and 10 no actual threat (e.g., a knife or a barking dog) pictures in the threatening condition, and 9 social pleasure (e.g., babies playing together) and 11 nonsocial pleasure (e.g., beautiful scenery) pictures in the pleasant condition. Also see Appendix S1 for more details.

Behavioral rating session

After the fMRI session, participants evaluated how disgusting, threatening, or pleasant were each of the 80 pictures according to a 9-point Likert scale. For the pleasant rating, 1 indicates "extremely unpleasant", 5 indicates "neutral" and 9 indicates "extremely pleasant". For the disgusting and threatening ratings, 1 indicates "not at all", 5 indicates "moderately", and 9 indicates "extremely". Ratings for each condition of emotion were elicited in separate blocks. The order of blocks was counterbalanced across participants. Importantly, participants did not know they had to evaluate the pictures when participating in the fMRI session. The rating session took approximately 20-30 minutes. **Table S2** summarizes the behavioral ratings of all pictures in each rating category, which are consistent with previously known IAPS valences and picture conditions.

Survey session

After the rating session, participants filled out three computer-based questionnaires. The first questionnaire (Appendix S2A) asked their political attitudes and involvement as well as their religiousness. Participants' political orientations were based on three components: their ideological position (item#1), partisan affiliation (item#2), and their policy preferences on the Wilson-Patterson questionnaire (Wilson-Patterson Issue Battery). Each component was first converted to a normalized score such that its minimum score is 0 and maximum score is 1 (e.g., if a participant selects "2. moderate, leaning liberal" on item#1, its normalized score is (2-1)/4 = 0.25). The political orientation (attitude) score was created by equally weighting the three components (min = 0 and max = 1). We also asked for whom they planned to vote (until November 6, 2012) or for whom they actually voted (after November 6, 2012). The second questionnaire (State-Trait Anxiety Inventory) assessed the state and trait anxiety [S2], and the third questionnaire (Disgust Scale-Revised) assessed the individual differences in disgust sensitivity (Appendix S2B) [S3, S4]. It took approximately 10-20 minutes to complete the survey session. Participants were debriefed and thanked for their participation after the survey session.

Relationship of political attitudes with demographic and other variables

We examined whether demographic data (age, sex) or other study variables (political interest, religiousness, trait anxiety, state anxiety, disgust sensitivity) are related to political attitudes. When we examined their relationships by simple correlations across all 83 participants, religiousness ($r(81) = 0.284$, $p = 0.009$), age ($r(81) = -0.232$, $p = 0.035$), and sex ($r(81) = 0.221$, $p = 0.045$) were significantly correlated with political attitudes at $p < 0.05$ (uncorrected). However, this type of uncorrected correlation analysis with multiple predictors is more likely to produce "false alarms". Thus, we next conducted a hierarchical Bayesian multiple regression analysis [S5, S6], which assigns a higher-level distribution across the regression coefficients of multiple predictors. Specifically, regression coefficients are coming from a t-distribution and its parameters (mean, scale, and df) are estimated from data, modeling a typical scenario of regression analyses (i.e., regression coefficients of many predictors are nearly zero and only a few predictors have non-zero regression coefficients). Because of the hierarchical structure, we are much less likely to have falsely significant results. See [S5, S6] for more details and the code for hierarchical Bayesian multiple regression ("MultiLinRegressHyperJags.R") is available at John K. Kruschke's website (*http://www.indiana.edu/%7Ekruschke/DoingBayesianDataAnalysis/Programs/*).

The results with the hierarchical Bayesian multiple regression showed that neither age nor sex was credibly associated with political attitude. However, political attitude was associated with religiousness (mean beta coefficient = 0.07, 95% highest density interval (HDI) of its posterior distribution = [0.019, 0.117]). Here, we used a heuristic Bayesian decision rule [S6]: a predictor is credibly associated with our independent variable if the 95% HDI of the predictor excludes zero. Note that the relationship between religiousness and conservatism has been well documented in previous literature (e.g., [S7, S8]).

We added age and sex as covariates in our standard General Linear Model (GLM) imaging analyses. For elastic net analysis (see below for the details), we used only fMRI data as predictors to test how accurately we can predict political attitudes with neural data alone. When we add age and sex as additional regressors to neural data for the elastic net analysis, both age and sex fail to survive at all (survival rate = 0.000), which means all the results using the elastic net approach remain unchanged even when we add the demographic variables.

Test-retest reliability of political attitudes

For a subset of participants (n=32), we re-assessed their political attitudes after some interval (mean = 171.7 days, SD = 142.1 days). The test-retest correlation coefficient was very high (Pearson correlation: $r(30) = 0.952$, $p < 2.2E-16$; Robust correlation: $r = 0.986$, $p < 2.0E-16$). The interval between the first and the second assessments was not significantly related to the difference of political attitudes between two assessments ($r(30) = 0.080$, $p = 0.662$).

fMRI data analysis

Image acquisition and preprocessing analysis: The anatomical and functional imaging sessions were conducted on a 3.0 tesla Siemens Magnetom Trio scanner at VTCRI. High-resolution T1-weighted scans (1x1x1 mm^3) were acquired using an MP-RAGE sequence (Siemens). Functional images were collected using echo-planar imaging with repetition time (TR) = 2,000ms and echo time (TE) = 25ms. 37 4mm slices (voxel size = 3.4 x 3.4 x 4mm^3) were acquired after angled 30 degrees with respect to the anteroposterior commissural line [S9].

Functional data were first spike-corrected to reduce the impact of artifacts using AFNI's 3dDespike (http://afni.nimh.nih.gov/afni). Data were subsequently preprocessed with SPM8 (http://www.fil.ion.ucl.ac.uk/spm/software/spm8/) for slice-timing correction using the first slice as the reference slice, motion correction, coregistration, gray/white matter segmentation, normalization to the Montreal Neurological Institute (MNI) template, and spatial smoothing using an 8mm full-width/half-maximum Gaussian kernel. Postprocessing voxels were 4 x 4 x 4 mm^3.

General linear model for standard fMRI analyses: All standard imaging analyses were conducted using the General Linear Model (GLM) implemented in SPM8. All first level analyses included a temporal high-pass filter (128s) and order 1 temporal autocorrelation (AR(1)) was assumed. For the main effects of emotional processing, the first level GLM was specified for each participant. The onsets for each picture subcondition (core/contamination disgust, animal reminder disgust, actual threat, no actual threat, social pleasure, nonsocial pleasure) and fixation crosses were convolved with a canonical hemodynamic response function (with time and dispersion derivatives) using a delta function of zero duration (or reaction time (RT) for fixation crosses) in SPM8. Initially, we probed to examine the separate neural correlates of emotional subconditions, but we collapsed them within each emotional condition to simplify the analysis. Six head motion parameters were also included in the first level GLM as covariates. The

random-effects analyses were conducted using the contrast images from the first level GLM. We separately examined the maps of [Disgusting - Neutral], [Threatening - Neutral], and [Pleasant - Neutral] contrasts (see **Figure S2** for family-wise error (FWE) cluster-level corrected maps and also **Table S3**).

To examine the neural correlates of political attitudes, for each contrast of [Disgusting - Neutral], [Threatening - Neutral], and [Pleasant - Neutral], we conducted independent t-tests comparing the Liberal and Conservative groups at the 2nd level GLM. Age and sex were added in the 2nd level analysis as covariates. To report clusters of voxels significantly related to political attitudes, we used a height threshold of $p < 0.005$ with extent threshold of $k \geq 10$ voxels or FWE cluster-level correction ($k \geq 107$) (**Figure S2D**), which was implemented in *CorrClusTh* by Thomas Nichols (http://go.warwick.ac.uk/tenichols/scripts/spm/spm8/CorrClusTh.m).

fMRI results on individual differences using standard GLM analysis: The contrasts with threatening or pleasant pictures revealed no regions surviving multiple corrections. However, in the [Disgusting > Neutral] contrast, the Conservative group showed greater activity than the Liberal group in several regions (**Figure S2D** and **Table S4**), including striatum, globus pallidus, thalamus, periaqueductal gray, substantia nigra, hippocampus, amygdala, dorsolateral prefrontal cortex, anterior cingulate cortex, pre-supplementary motor area, fusiform gyrus, and middle/superior temporal gyrus. No regions survived correction for multiple comparisons for the [Liberal group > Conservative group] comparison.

Preparation of data for penalized logistic regression analysis: For penalized logistic regression analysis, we prepared the data as described below (**Figure S3A**). First, we extracted a map of the [Disgust > Neutral] contrast for each participant. Then, we applied an a priori mask, which was generated from the Neurosynth website (www.neurosynth.org) [S10] containing a total of 7,471 voxels (voxel size = 4 x 4 x 4 mm^3); that is, we only used voxels inside the mask as predictors in the following analysis. We obtained the union of meta-analytic (positively correlated and both forward and reverse inference) maps of "Emotion" and "Attention" terms, thresholded at $q < 0.05$ False Discovery Rate corrected. Then, we generated a matrix (matrix size = number of participants x number of voxels) by reshaping each participant's contrast maps (three-dimensional data) into one-dimensional vectors for penalized regression analysis.

Cross-validated penalized logistic regression analysis: We used the elastic net [S11], a penalized (logistic) regression technique, to make cross-validated predictions of political group membership and select voxels important for such predictions. Here, we first briefly explained the penalization technique and its relationship to the ordinary regression analysis. We used the continuous regression analysis as an example because of its relative easiness to understand. For more detailed reviews, see [S11-S14].

In the usual multiple linear regression analysis, we have N observations (participants), p number of regressors (or predictors, $x_i = (x_{i1}, x_{i2}, \ldots, x_{ip})$), and N number of responses (y_i). For

example, p is the number of voxels (p = 7,471), x_i are subjects' beta coefficients of each voxel (x_i = a matrix whose size is N x p), and y_i is an individual difference measure (e.g., y_i are political ideology scores, a vector of length N). In ordinary multiple linear regression, the objective function ($f(\theta)$) to minimize is: $f(\theta)=\sum_{i=1}^{N}(y_i-\beta_0-x_i^T\cdot\beta)^2$ where β_0 is the intercept and β are regression coefficients (= a vector of length p). When there are too many predictors (i.e., $p \gg N$), it is well known that the ordinary multiple linear regression performs poorly in prediction accuracy (generalization) and interpretation [S11]. To improve the performance, several penalization methods have been proposed, which impose an L1 penalty, an L2 penalty, or both. The L1 penalty refers to when $\|\beta\|_1=\sum_{j=1}^{p}|\beta_j|$ (= sum of absolute values of coefficients) is constrained and the L2 penalty refers to when $\|\beta\|_2^2=\sum_{j=1}^{p}\beta_j{}^2$ (= sum of squared values of coefficients) is constrained when estimating regression coefficients. The elastic net that we used in our work employs both L1 and L2 penalization. As suggested by previous works [S15-S18], the elastic net has several advantages for fMRI data compared to other machine learning techniques such as the conventional Support Vector Machine (SVM) [S19] or the least absolute shrinkage and selection operator (LASSO) [S12]. First, like other penalized regression techniques (c.f., ridge regression), it does continuous shrinkage and automatic selection of predictors (i.e., the regression coefficients of unimportant predictors shrink to zero) due to L1 penalization. Thus, the elastic net automatically selects voxels that are critical for out-of-sample prediction accuracy (i.e., automatic variable selection), which increases the interpretability of the findings. Second, the elastic net enjoys a grouping effect, which clusters highly correlated predictors into a set of groups due to L2 penalization. Also, in fMRI studies, the number of predictors (p) is much greater than the number of subjects (N) (i.e., $p \gg N$) where the conventional LASSO is an inadequate choice (c.f., [S20, S21]).

The elastic net regression has two tuning parameters (α and λ), which are selected by cross validation (CV) over two-dimensional surface (**Figure 2D**). In the elastic net regression, the objective function to minimize for the Gaussian family is: $f(\theta)+\lambda\left[(1-\alpha)\|\beta\|_2^2/2+\alpha\|\beta\|_1\right]$ where the second term is called the *elastic net penalty* and $f(\theta)$ is the objective function for the ordinary multiple linear regression: $f(\theta)=\frac{1}{2N}\sum_{i=1}^{N}(y_i-\beta_0-x_i^T\beta)^2$. The tuning parameter λ ($\lambda \geq 0$) governs the overall complexity of the model (λ values close to zero approach the full (non-penalized) regression model), and α ($0 \leq \alpha \leq 1$) is the elastic-net mixing parameter, which compromises between the ridge (α = 0) and the LASSO (α = 1). For a penalized binomial logistic regression [S13], the objective function to minimize has the same form and the elastic net penalty: $f(\theta)+\lambda\left[(1-\alpha)\|\beta\|_2^2/2+\alpha\|\beta\|_1\right]$, but with different y_i (e.g., y_i = 0 for the Liberal

group and 1 for the Conservative group) and $f(\theta)$: $f(\theta) = -\left[\frac{1}{N}\sum_{i=1}^{N} y_i \cdot (\beta_0 + x_i^T \beta) - \log(1 + \exp(\beta_0 + x_i^T \beta))\right]$.

For the selection of tuning parameters and estimation of the elastic net penalized logistic regression model, we used the *glmnet* package for Matlab (http://www.stanford.edu/~hastie/glmnet_matlab/) and R [S14]. Regressors were standardized prior to fitting the model, which is a standard routine for a penalized logistic regression analysis. We also used a built-in Matlab function (*cvglmnet.m*) in the glmnet package for CV and another built-in function (*glmnetPredict.m*) for making predictions. Two authors (W.-Y.A. and T.L.) independently tested the penalized logistic regression procedure and verified the findings.

For the selection of tuning parameters, we first estimated the elastic-net mixing parameter (α) and then the overall complexity parameter (λ). For the estimation of α, we conducted the following procedure over 1,000 grids of α (α = 0.001, 0.002, 0.003, … , 0.998, 0.999, 1.000):

1.1. At each α value, first fits the elastic net (LASSO if α = 1) model paths to get the λ sequence.

1.2. Divide data randomly into 10 partitions.

   1.2.1. Train the model based on 90% of the data (9 partitions = 50 subjects' data) and compute the minimum binomial deviance (at the λ value that minimizes the binomial deviance) on the 10% of the data (1 partition = 6 subjects' data).

   1.2.2. Repeat step 1.2.1 10 times and compute the average binomial deviance over the 10 repetitions. Calling *cvglmnet.m* (in Matlab) command runs both steps 1.2.1 and 1.2.2.

1.3. CV performance can vary slightly depending on how the data set is divided into K (=10) partitions. To get more reliable estimates, repeat the whole steps 1.1 and 1.2 twenty times and compute the average binomial deviance over the 20 repetitions, which is in fact the average binomial deviance over 200 (=10*20) iterations at each α.

As seen in **Figure 2D** (red dashed line in step 2), α value of 0.026 minimized the average binomial deviance. Setting α to 0.026, we estimated the second tuning parameter, overall complexity λ. While α is set to 0.026, we first estimated the trace plot of coefficient values for each λ value (**Figure 2D**, left figure in step 3). Then, we repeated the following procedure 1,000 times:

2.1. Run a 10-fold CV (similar to steps 1.2.1 and 1.2.2 but with setting α to 0.026) to find the λ value that minimizes the binomial deviance and one that is +1 S.E. from the minimum λ value (**Figure 2D**, two dashed lines in the right figure in step 3. The numbers on top of x-axis are the number of survived regressors for each λ value).

2.2. Extract β coefficients of all regressors (voxels) using the λ value that is +1 S.E. from the minimum (i.e., +1 S.E. λ). Using +1 S.E. λ is a heuristic strategy to produce a less complex and more conservative model [S22]. The β coefficients of many regressors would shrink to zero. Record whether each regressor (voxel) survived (β coefficient is non-zero) or not (β coefficient is zero) in the current iteration.
2.3. Make predictions (fitted probabilities that range from 0 to 1) for the political group based on the β coefficients of the penalized logistic regression model. Then compute the area under the curve (AUC) of the receiver operating characteristic (ROC) curve.

Calculating the "survival rate" of voxels: After finishing 1,000 CVs, we generated a matrix that indicates whether each voxel survived (1 if its β coefficient is non-zero, 0 if its β coefficient is zero) CV on each iteration (= a matrix size of 1,000 x 7,471, **Figure 2D**, step 4). We defined the 'survival rate' as the proportion of each voxel surviving CV over 1,000 iterations (i.e., 0 indicates that the voxel survived 0 times out of 1,000 iterations and 1 indicates that it survived all 1,000 iterations). Survival rate is closely related to regression coefficients (**Figure S3B**). We also generated another matrix that contained the signs of mean β coefficients over 1,000 iterations. To separately visualize voxels that predict either the Conservative ($\beta > 0$) or the Liberal group ($\beta < 0$), we projected the [survival rate x the sign of the mean β coefficient] of each voxel back into the brain (voxel) space (**Figure 3A**).

Checking the validity of the elastic net model: To make sure our elastic net procedure is unbiased toward finding group differences, we tested if the penalized logistic regression can predict permuted outcome variable [S20]. In other words, we permuted (randomly sampled) our outcome variable (political ideology group membership), and let the elastic net model predict the permuted variable. If our elastic net model is valid and unbiased, AUC for permuted data should be 0.5 (at chance level). Indeed, mean AUC of the penalized logistic regression model was 0.50 over 1,000 iterations, which confirms that our model is unbiased.

Supplementary Information for **Figure 3B**, histogram of CV ROC AUCs: In order to more fully demonstrate the out-of-sample performance of the penalized logistic regression analysis, we did the following. First, following the main approach in the text, we categorized participants' political attitudes by terciles, and then restricted our analysis to the low (Liberal group) and high (Conservative group) terciles. We then repeated 1,000 times 1) Run the CV procedure implemented by *cvglmnet.m* and find the value of λ and the beta coefficients of predictors that minimizes binomial deviance. Here we use 5-fold cross validation to have enough sample size for computing AUC. We set the mixing parameter (α) to 0.026; 2) for each iteration at the minimum λ, and for each of the five folds, use the stored fits on the data not including the fold (80% of the data) from the iteration's CV model to predict the class probabilities for the data in the fold. Then use the MATLAB *perfcurve* function to compute the receiver operating

characteristic curve (ROC), and the area under this curve. 5 AUCs are computed for each iteration, giving a total of 5,000 data points for the histogram.

Penalized linear regression analysis: **Figure 3A** shows the voxels critical for cross-validated classification accuracy for predicting Liberal and Conservative group membership. In **Figure S4**, we used panelized linear regression, predicting political attitudes in a continuous fashion across all participants (n=83). The procedure is identical to cross-validated penalized logistic regression analysis reported above except the following: First, we used penalized linear regression, which has a different objective function to minimize for the Gaussian family as described above. Second, we re-estimated α (mixing parameter) value that is optimized for penalized linear regression across all participants. The estimated α value optimized for penalized linear regression was 0.222, but we report the results with both of the α values (for **Figure S4A**, we used 0.026, which was optimized for penalized logistic regression and for **Figure S4B**, we used 0.222). Third, we used a correlation coefficient as a measure of predictive accuracy. In each of 1,000 iterations, we first computed each participant's predicted political attitude. After 1,000 iterations, we computed each participant's mean predicted political attitudes over 1,000 iterations. Then, we calculated the correlation coefficient between actual political attitudes and the averaged predicted political attitudes. As seen in **Figures 3A, S4A, and S4B**, regions predicting continuous political attitudes substantially overlap with regions predicting Liberal and Conservative group membership. Right panels in **Figures S4A and S4B** show the correlations between actual political attitudes and predicted political attitudes from the BOLD data, with each of the two α values.

Out-of-sample prediction using the split half approach: **Figures S4A and S4B** show the voxels critical for predicting individual differences in political ideology using data from all the participants (n=83). Alternatively, we tested if our machine learning procedure can make accurate predictions on the half of the data (test set) when the model is trained on the other half of the data (training set). To test it, participants were first sorted based on their political ideology scores (i.e., sorted participant#1 is the most liberal participant, and sorted participant#83 is the most conservative participant). Then, participants #1, #3, #5, …, #81, #83 were used as the test set, and participants #2, #4, #6, …, #80, #82 were used as the training set. We used this procedure to ensure that training and test sets are widely and evenly distributed across the whole spectrum of political ideology scores.

We used the identical procedure reported for the penalized linear regression analysis except the following: First, we only used the training set (n=41) to fit the machine learning (elastic net) model (i.e., estimation of λ and the beta coefficients of predictors). Second, the predictions were made only on the test set (n=42) based on the model estimated only with the training set. Third, we used the minimum λ (instead of +1 S.E. λ) when making the predictions on the test set to achieve best accuracy. We found that many regions (e.g., striatum, thalamus, inferior parietal lobule) overlap substantially with what we found from using all participants

(**Figure S4**). The out-of-sample prediction performance (correlation coefficient between actual and predicted political attitudes) on the test set was 0.52 (**Figure 3B (figure inset)**, p = 0.0004, robust r = 0.44, robust p = 0.0024). The correlation value is lower than when we computed cross-validated predictive accuracy across all participants (e.g., **Figures S4A and S4B**), but we should keep in mind that we used only 41 participant' data for the training of the elastic net model, and performance of a machine learning model crucially depends on the amount of data we can use for the training. Also note that model performance can vary depending on how we divide the data into test and training sets. We found that the out-of-sample prediction performance (correlation coefficient) varied between 0.40 and 0.52 when we divided the data in different ways (e.g., using the one third (33%) of the data as the test set and two thirds (67%) of the data as the training set). The correlation coefficients were always highly significant ($p < 0.003$).

Single-stimulus fMRI analysis: The elastic net analysis described above used the whole brain [Disgusting > Neutral] contrast map (the number of predictors = 7,741), collected approximately for 20 minutes. Here, we used the BOLD time series data of the first disgusting stimulus only (20 seconds), which was spatial averaged from all the (+) voxels (**Figure 3A** red-to-yellow regions).

Figure S3C illustrates how we prepared the data for the single-stimulus fMRI analysis. In each participant, we extracted raw BOLD time series during the whole experiment that are spatially averaged within each of two types of regions (i.e., (+) voxels or (-) voxels). Each voxel was equally weighted in each mask. After applying a high pass filter and linearly interpolating time series data every 1s, we captured the data for the period 2 seconds prior to the first disgusting stimulus presentation to 16 seconds post-stimulus onset (a total of 20 seconds) every TR (= 2s). Thus, each subject had 11 time points or predictors. BOLD signals were converted to percentage signal change after correcting the baseline activity using the mean baseline value (2 seconds prior to 0 seconds post stimulus). Then, we combined all participants' data into a single matrix (matrix size = number of participants x number of time points) (**Figure S3C, right side**). When conducting the single-trial fMRI analysis on each region of interest (ROI), we extracted averaged raw time series from each region only (**Figures 4C**).

The remaining steps for penalized logistic regression analysis remained the same: We estimated a tuning parameter (λ) and the beta coefficients of predictors using CV and computed the survival rate and the AUC of the ROC curve. We used the α value (= 0.026) estimated from the full-data analysis, but for the single-stimulus fMRI analysis when $p < n$, AUC values did not depend much on α value.

Single-stimulus fMRI analysis on independent ROIs: We used two ROIs (amygdala and posterior insula) from a previous study [S1] for a single-stimulus analysis. To our knowledge, Schreiber et al. (2013) is the only study that found neural correlates of political orientation using non-political stimuli. For each ROI, we first generated a 8mm sphere centered on its peak coordinates (Amygdala=[20, -6, -10], posterior insula=[-36, -40, 18] in Talairach coordinates, which were converted to MNI coordinates using http://noodle.med.yale.edu/~papad/mni2tal/).

Then we conducted single-stimulus analysis as we described above. We found that mean AUC of the amygdala ROI (**Figure S4C**) was 0.565 (SD = 0.098) when we used +1 S.E. λ. With minimum λ that maximizes predictive accuracy, mean AUC was 0.745 (SD = 0.025). AUC of posterior insula was 0.500 (= at chance level) with either +1 S.E. or minimum λ, which is consistent with what we found in our own peak ROI of posterior insula. Although AUC with the amygdala ROI from [S1] is worse than AUC with our peak amygdala/hippocampus ROI (mean AUC = 0.721 with +1 S.E. λ), we should keep in mind that [S1] used a risky decision-making paradigm, which is very different from our passive-viewing paradigm using emotionally evocative images.

<u>Group comparisons on the characteristics of the first disgusting pictures:</u> We checked IAPS valence ratings, IAPS arousal ratings, and onsets of the first disgusting pictures in each political group (**Table S1**). None of IAPS ratings were significantly different between Liberal and Conservative groups (IAPS valence, t(54) = 0.336, p = 0.738; IAPS arousal, t(54) = 0.891, p = 0.377). The conservative group had marginally longer onset time than the Liberal group (t(54) = 1.891, p = 0.064). Group difference on subconditions of the first disgusting stimuli was not significant between the Liberal and Conservative groups ($\chi^2(1)=0.072$, p=0.789).

Supplemental References

S1. Schreiber, D., Fonzo, G., Simmons, A. N., Dawes, C. T., Flagan, T., Fowler, J. H., and Paulus, M. P. (2013). Red Brain, Blue Brain: Evaluative Processes Differ in Democrats and Republicans. PloS one *8*, e52970. Available at: http://www.plosone.org/article/info%3Adoi%2F10.1371%2Fjournal.pone.0052970#s5.

S2. Spielberger, C. D., and Gorsuch, R. L. (1983). Manual for the State-trait anxiety inventory (form Y) ("self-evaluation questionnaire").

S3. Haidt, J., McCauley, C., and Rozin, P. (1994). Individual differences in sensitivity to disgust: A scale sampling seven domains of disgust elicitors. Pers. Individ. Dif. *16*, 701–713.

S4. Olatunji, B. O., Williams, N. L., Tolin, D. F., Abramowitz, J. S., Sawchuk, C. N., Lohr, J. M., and Elwood, L. S. (2007). The Disgust Scale: Item analysis, factor structure, and suggestions for refinement. Psychol. Assessment *19*, 281–297.

S5. Ahn, W.-Y., Vasilev, G., Lee, S.-H., Busemeyer, J. R., Kruschke, J. K., Bechara, A., and Vassileva, J. (2014). Decision-making in stimulant and opiate addicts in protracted abstinence: evidence from computational modeling with pure users. Front Decision Neurosci *5*.

S6. Kruschke, J. K. (2011). Doing Bayesian Data Analysis (Burlington, MA: Elsevier).

S7. Brandt, M. J., and Reyna, C. (2010). The Role of Prejudice and the Need for Closure in Religious Fundamentalism. Pers. Soc. Psychol. B. *36*, 715–725.

S8. Duriez, B., Luyten, P., and Snauwaert, B. (2002). The importance of religiosity and values in predicting political attitudes: Evidence for the continuing importance of religiosity in Flanders (Belgium). Mental Health *5*, 35–54.

S9. Deichmann, R., Gottfried, J. A., Hutton, C., and Turner, R. (2003). Optimized EPI for fMRI studies of the orbitofrontal cortex.

S10. Yarkoni, T., Poldrack, R. A., Nichols, T. E., Van Essen, D. C., and Wager, T. D. (2011). Large-scale automated synthesis of human functional neuroimaging data. Nat. Methods *8*, 665–670.

S11. Zou, H., and Hastie, T. (2005). Regularization and variable selection via the elastic net. J. R. Stat. Soc. Ser. B. *67*, 301–320.

S12. Tibshirani, R. (1996). Regression shrinkage and selection via the lasso. J. R. Stat. Soc. Ser. B Stat. Methodol., 267–288.

S13. Simon, N., Friedman, J., and Hastie, T. (2011). Regularization paths for Cox's proportional hazards model via coordinate descent. J. Stat. Softw.

S14. Hastie, T. (2010). Regularization paths for generalized linear models via coordinate descent. *33*, 1. Available at: http://www.ncbi.nlm.nih.gov/pmc/articles/PMC2929880/.

S15. Ryali, S., Chen, T., Supekar, K., and Menon, V. (2012). Estimation of functional connectivity in fMRI data using stability selection-based sparse partial correlation with elastic net penalty. *59*, 3852–3861.

S16. Ryali, S., Supekar, K., Abrams, D. A., and Menon, V. (2010). Sparse logistic regression for whole-brain classification of fMRI data. *51*, 752–764.

S17. Smith, A., Bernheim, B. D., Camerer, C. F., and Rangel, A. (2014). Neural Activity Reveals Preferences Without Choices. Am. Econ. J.-Microecon. *6*, 1–36.

S18. Whelan, R., Watts, R., Orr, C. A., Althoff, R. R., Artiges, E., Banaschewski, T., Barker, G. J., Bokde, A. L. W., Büchel, C., Carvalho, F. M., et al. (2014). Neuropsychosocial profiles of current and future adolescent alcohol misusers. Nature *512*, 185–189.

S19. Cortes, C., and Vapnik, V. (1995). Support-vector networks. Mach. Learn.

S20. Wager, T. D., Atlas, L. Y., Leotti, L. A., and Rilling, J. K. (2011). Predicting Individual Differences in Placebo Analgesia: Contributions of Brain Activity during Anticipation and Pain Experience. J. Neurosci. *31*, 439–452.

S21. Wager, T. D., Atlas, L. Y., Lindquist, M. A., Roy, M., Woo, C.-W., and Kross, E. (2013). An fMRI-based neurologic signature of physical pain. N. Engl. J. Med. *368*, 1388–1397.

S22. Hastie, T. J., Tibshirani, R. J., and Friedman, J. J. H. (2009). The elements of statistical learning (Springer).

Appendix S1. IAPS valence and arousal ratings of stimuli in each (sub)condition

IAPS picture number	Condition	Subcondition	Valence	Arousal	Description
1111	disgusting	core/contamination	3.25	5.20	Snakes
1274	disgusting	core/contamination	3.17	5.39	Roaches
7360	disgusting	core/contamination	3.59	5.11	FliesOnPie
7380	disgusting	core/contamination	2.46	5.88	RoachOnPizza
9008	disgusting	core/contamination	3.47	4.45	Needle
9290	disgusting	core/contamination	2.88	4.40	Garbage
9300	disgusting	core/contamination	2.26	6.00	Dirty
9320	disgusting	core/contamination	2.65	4.93	Vomit
9390	disgusting	core/contamination	3.67	4.14	Dishes
9570	disgusting	animal reminder	1.68	6.14	Dog
3010	disgusting	animal reminder	1.79	7.26	Mutilation
3030	disgusting	animal reminder	1.91	6.76	Mutilation
3060	disgusting	animal reminder	1.79	7.12	Mutilation
3102	disgusting	animal reminder	1.40	6.58	Burn victim
3130	disgusting	animal reminder	1.58	6.97	Mutilation
3150	disgusting	animal reminder	2.26	6.55	Mutilation
3170	disgusting	animal reminder	1.46	7.21	Baby tumor
3250	disgusting	animal reminder	3.78	6.29	OpenChest
3266	disgusting	animal reminder	1.56	6.79	Injury
9405	disgusting	animal reminder	1.83	6.08	Sliced hand
2485	neutral	neutral	5.69	3.74	Man
2514	neutral	neutral	5.19	3.50	Woman
7004	neutral	neutral	5.04	2.00	Spoon
7010	neutral	neutral	4.94	1.76	Basket
7035	neutral	neutral	4.98	2.66	Mug
7095	neutral	neutral	5.99	4.21	Headlight
7100	neutral	neutral	5.24	2.89	Fire hydrant
7140	neutral	neutral	5.50	2.92	Bus
7150	neutral	neutral	4.72	2.61	Umbrella
7170	neutral	neutral	5.14	3.21	Light Bulb
7175	neutral	neutral	4.87	1.72	Lamp
7180	neutral	neutral	4.73	3.43	NeonBuilding
7190	neutral	neutral	5.55	3.84	Clock
7224	neutral	neutral	4.45	2.81	File cabinets
7233	neutral	neutral	5.09	2.77	Plate
7235	neutral	neutral	4.96	2.83	Chair

7491	neutral	neutral	4.82	2.39	Building
7500	neutral	neutral	5.33	3.26	Building
7550	neutral	neutral	5.27	3.95	Office
7595	neutral	neutral	4.55	3.77	Traffic
1440	pleasant	nonsocial	8.19	4.61	Seal
1460	pleasant	nonsocial	8.21	4.31	Kitten
1710	pleasant	nonsocial	8.34	5.41	Puppies
1750	pleasant	nonsocial	8.28	4.10	Bunnies
1920	pleasant	nonsocial	7.90	4.27	Porpoise
2040	pleasant	nonsocial	8.17	4.64	Baby
2070	pleasant	nonsocial	8.17	4.51	Baby
5760	pleasant	nonsocial	8.05	3.22	Nature
5849	pleasant	nonsocial	6.65	4.89	Flowers
7502	pleasant	nonsocial	7.75	5.91	Castle
8190	pleasant	nonsocial	8.10	6.28	Skier
2080	pleasant	social	8.09	4.70	Babies
2091	pleasant	social	7.68	4.51	Girls
2165	pleasant	social	7.63	4.55	Father
2360	pleasant	social	7.70	3.66	Family
2530	pleasant	social	7.80	3.99	Couple
2540	pleasant	social	7.63	3.97	Mother
2550	pleasant	social	7.77	4.68	Couple
5831	pleasant	social	7.63	4.43	Seagulls
8496	pleasant	social	7.58	5.79	Water slide
3500	threatening	actual threat	2.21	6.99	Attack
6350	threatening	actual threat	1.90	7.29	Attack
6550	threatening	actual threat	2.73	7.09	Attack
6821	threatening	actual threat	2.38	6.29	Gang
6838	threatening	actual threat	2.45	5.80	Police
9050	threatening	actual threat	2.43	6.36	Plane Crash
9622	threatening	actual threat	3.10	6.26	Jet
9910	threatening	actual threat	2.06	6.20	Auto accident
9911	threatening	actual threat	2.30	5.76	Car accident
9920	threatening	actual threat	2.50	5.76	Auto accident
1120	threatening	no actual threat	3.79	6.93	Snake
1302	threatening	no actual threat	4.21	6.00	Dog
2120	threatening	no actual threat	3.34	5.18	AngryFace
5972	threatening	no actual threat	3.85	6.34	Tornado
6200	threatening	no actual threat	3.20	5.82	AimedGun
6242	threatening	no actual threat	2.69	5.43	Gang
6260	threatening	no actual threat	2.44	6.93	AimedGun

6510	threatening	no actual threat	2.46	6.96	Attack
6830	threatening	no actual threat	2.82	6.21	Guns
9630	threatening	no actual threat	2.96	6.06	Bomb

Appendix S2: Survey Instrument

All participants completed the computer-based survey questionnaires. The questions and their coding scales for political ideology and disgust sensitivity are reproduced below.

A. Political (items 1-8) and religious (items 9 and 10) questions

1. Labels are often misleading, but in general do you consider yourself liberal, conservative, or something in between

    1. liberal
    2. moderate, leaning liberal
    3. moderate
    4. moderate, leaning conservative
    5. conservative

2. In general, do your consider yourself a Democrat, a Republican, or an Independent?

    1. strong Democrat
    2. weak Democrat
    3. Independent, leaning Democrat
    4. Independent
    5. Independent, leaning Republican
    6. weak Republican
    7. strong Republican
    8. other

3. (Until November 6, 2012) If the 2012 presidential election were held today, whom would you vote for? (After November 6, 2012) Who did you vote for President in 2012?

    (answer order was randomized for each participant)

    1. Barack Obama
    2. Mitt Romney
    3. Other
    4. Not voting
    5. Not sure

4. How interested are you in politics and public affairs?

    1. very interested
    2. somewhat interested
    3. not very interested
    4. not at all interested

5. How strongly would you say you feel about political issues? Imagine a scale of feelings ranging from 1-10 with 1 representing no feelings at all and 10 representing intense feelings, and place yourself on this scale.

1. 1—no feelings at all on political issues
2. 2
3. 3
4. 4
5. 5—moderately strong feelings about political issues
6. 6
7. 7
8. 8
9. 9
10. 10—intense feelings about political issues

6. For each of the following, note whether it is something you have ever done.

a. attended a political meeting or rally
b. worked in a political campaign in any capacity (even for no pay)
c. contributed money to a political cause, party, or candidate
d. held any governmental office no matter how minor
e. communicated your thoughts or requests to a governmental official

    1. yes
    2. no

7. How would you describe your voting behavior?

1. I vote in nearly every election.
2. I vote in most elections.
3. I rarely vote.
4. I never vote.
5. I am ineligible to vote.

8. How often do you have discussions about politics with others?

1. very often
2. somewhat often
3. rarely
4. never

9. How often do you attend religious services?

a. Never or very rarely
b. Occasionally
c. Once per week

d. More than once per week

10. Do you regularly say grace before meals?
    a. Always
    b. Usually
    c. Occasionally
    d. Never

<u>Wilson-Patterson Issue Battery</u>

Here is a list of various topics. Please indicate how you feel about each topic.

1. strongly agree
2. agree
3. uncertain
4. disagree
5. strongly disagree

a. School prayer
b. Pacifism
c. Stop immigration
d. Death penalty
e. Government-arranged healthcare
f. Premarital sex
g. Gay marriage
h. Abortion rights
i. Evolution
j. Biblical truth
k. Increase welfare spending
l. Protect gun rights
m. Increase military spending
n. Government regulation of business
o. Small government
p. Foreign aide
q. Lower taxes
r. Stem cell research
s. Abstinence-only sex education
t. Allow torture of terrorism suspects

B. Disgust Scale – Revised (http://people.stern.nyu.edu/jhaidt/Dscale-R.doc)

Please indicate how much you agree with each of the following statements, or how true it is about you. Please write a number (0-4) to indicate your answer:

0 = Strongly disagree (very untrue about me)
1 = Mildly disagree (somewhat untrue about me)
2 = Neither agree nor disagree
3 = Mildly agree (somewhat true about me)
4 = Strongly agree (very true about me)

____1. I might be willing to try eating monkey meat, under some circumstances.
____2. It would bother me to be in a science class, and to see a human hand preserved in a jar.
____3. It bothers me to hear someone clear a throat full of mucous.
____4. I never let any part of my body touch the toilet seat in public restrooms.
____5. I would go out of my way to avoid walking through a graveyard.
____6. Seeing a cockroach in someone else's house doesn't bother me.
____7. It would bother me tremendously to touch a dead body.
____8. If I see someone vomit, it makes me sick to my stomach.
____9. I probably would not go to my favorite restaurant if I found out that the cook had a cold.
____10. It would not upset me at all to watch a person with a glass eye take the eye out of the socket.
____11. It would bother me to see a rat run across my path in a park.
____12. I would rather eat a piece of fruit than a piece of paper
____13. Even if I was hungry, I would not drink a bowl of my favorite soup if it had been stirred by a used but thoroughly washed flyswatter.
____14. It would bother me to sleep in a nice hotel room if I knew that a man had died of a heart attack in that room the night before.

How disgusting would you find each of the following experiences? Please write a number (0-4) to indicate your answer:

0 = Not disgusting at all
1 = Slightly disgusting
2 = Moderately disgusting
3 = Very disgusting
4 = Extremely disgusting

____15. You see maggots on a piece of meat in an outdoor garbage pail.
____16. You see a person eating an apple with a knife and fork
____17. While you are walking through a tunnel under a railroad track, you smell urine.
____18. You take a sip of soda, and then realize that you drank from the glass that an acquaintance of yours had been drinking from.
____19. Your friend's pet cat dies, and you have to pick up the dead body with your bare hands.
____20. You see someone put ketchup on vanilla ice cream, and eat it.

____21. You see a man with his intestines exposed after an accident.
____22. You discover that a friend of yours changes underwear only once a week.
____23. A friend offers you a piece of chocolate shaped like dog-doo.
____24. You accidentally touch the ashes of a person who has been cremated.
____25. You are about to drink a glass of milk when you smell that it is spoiled.
____26. As part of a sex education class, you are required to inflate a new unlubricated condom, using your mouth.
____27. You are walking barefoot on concrete, and you step on an earthworm.
