# Supplementary material for: Nonpolitical Images Evoke Neural Predictors of Political Ideology
Source: Curr Biol. 2014 Nov 17;24(22):2693–9. doi: 10.1016/j.cub.2014.09.050 (PMC4245707; doi:10.1016/j.cub.2014.09.050)
Supplement: Document S2. Article plus Supplemental Information [file mmc2.pdf]

Current Biology 24, 2693–2699, November 17, 2014 ©2014 The Authors http://dx.doi.org/10.1016/j.cub.2014.09.050

Report

Nonpolitical Images Evoke Neural Predictors of Political Ideology

Woo-Young Ahn,[1,2] Kenneth T. Kishida,[1,2] Xiaosi Gu,[2,3] Terry Lohrenz,[1,2] Ann Harvey,[1,2] John R. Alford,[4] Kevin B. Smith,[5] Gideon Yaffe,[6] John R. Hibbing,[5] Peter Dayan,[7] and P. Read Montague[1,2,3,8,*]
[1]Virginia Tech Carilion Research Institute, Virginia Tech, Roanoke, VA 24016, USA
[2]Computational Psychiatry Unit, Virginia Tech, Roanoke, VA 24016, USA
[3]Wellcome Trust Centre for Neuroimaging, University College London, 12 Queen Square, London WC1N 3BG, UK
[4]Department of Political Science, Rice University, Houston, TX 77251-1892, USA
[5]Department of Political Science, University of Nebraska-Lincoln, Lincoln, NE 68588-0328, USA
[6]Yale Law School, Yale University, New Haven, CT 06511, USA
[7]Gatsby Computational Neuroscience Unit, University College London, London WC1N 3AR, UK
[8]Department of Physics, Virginia Tech, Blacksburg, VA 24061, USA

Summary

Political ideologies summarize dimensions of life that define how a person organizes their public and private behavior, including their attitudes associated with sex, family, education, and personal autonomy [1, 2]. Despite the abstract nature of such sensibilities, fundamental features of political ideology have been found to be deeply connected to basic biological mechanisms [3–7] that may serve to defend against environmental challenges like contamination and physical threat [8–12]. These results invite the provocative claim that neural responses to nonpolitical stimuli (like contaminated food or physical threats) should be highly predictive of abstract political opinions (like attitudes toward gun control and abortion) [13]. We applied a machine-learning method to fMRI data to test the hypotheses that brain responses to emotionally evocative images predict individual scores on a standard political ideology assay. Disgusting images, especially those related to animal-reminder disgust (e.g., mutilated body), generate neural responses that are highly predictive of political orientation even though these neural predictors do not agree with participants' conscious rating of the stimuli. Images from other affective categories do not support such predictions. Remarkably, brain responses to a single disgusting stimulus were sufficient to make accurate predictions about an individual subject's political ideology. These results provide strong support for the idea that fundamental neural processing differences that emerge under the challenge of emotionally evocative stimuli may serve to structure political beliefs in ways formerly unappreciated.

*Correspondence: read@vt.edu
This is an open access article under the CC BY license (http://creativecommons.org/licenses/by/3.0/).

Results

We carried out a passive picture-viewing experiment to test the hypothesis that nonpolitical but affectively evocative images elicit brain responses that predict political ideology as assessed by a standard political ideology measure. Healthy volunteers (n = 83) were instructed to look at presented pictures while lying in the scanner, and, to control for attentiveness, we instructed them to press a button when a fixation cross appeared on the screen (Figure 1). Images were sampled from the International Affective Pictures database [14] and included disgusting, threatening, pleasant, and neutral images (see Appendix S1 available online). Each emotional condition had two subconditions (see the Supplemental Experimental Procedures). After the fMRI session, participants completed a behavioral rating session in which they rated all pictures they had seen in the scanner (using a nine-point Likert scale) as disgusting, threatening, or pleasant. Lastly, participants filled out computer-based questionnaires assessing their political attitudes, disgust sensitivity, and state/trait anxiety level. See the Supplemental Experimental Procedures for details of the behavioral rating and survey sessions.

Political ideology was summed from several survey items (Appendix S2), including ideological position, partisan affiliation, and policy preferences (e.g., gun control and immigration, presented in the well-known Wilson-Patterson format [15]). Survey items on political ideology were normalized continuously from 0 (extremely liberal) to 1 (extremely conservative) (see the Supplemental Experimental Procedures). Figure 2A shows its distribution across all participants. Political attitudes and interest did not show a significant linear relationship [$r(81) = -0.148$, $p = 0.182$], but instead showed a U-shaped curve (Figure S1A), indicating that greater political interest is associated with polarized political attitudes. When tested on a subset of participants, our measure of political attitudes shows excellent test-retest reliability (test-retest Pearson correlation coefficient = 0.952; Figure 2B). To focus our analyses on polarized political groups, we divided participants into three groups based on their political ideology scores (Table S1): liberal (n = 28), moderate (n = 27), and conservative (n = 28).

As seen in Figure 2C, groups did not significantly differ in subjective ratings of disgusting, threatening, or pleasant pictures (also see Table S2). Also, there were no significant group differences on self-report measures except that the conservative group had marginally higher disgust sensitivity than the liberal group [$t(54) = 1.711$, $p = 0.093$; Figure S1B and Table S1]. Note that emotional states can be implicit or nonconscious under some conditions [16]. Self-report measures may fail to detect some individual differences in disgust sensitivity [17].

Having characterized liberal and conservative groups behaviorally and confirmed blood-oxygen-level dependent (BOLD) responses to affective pictures (Figure S2 and Table S3), we used a machine-learning approach to predict individual differences in political orientation from the patterns of whole-brain BOLD responses. Specifically, we applied a penalized regression method called the elastic net [18] to our fMRI data (Figures 2D and S3 and the Supplemental

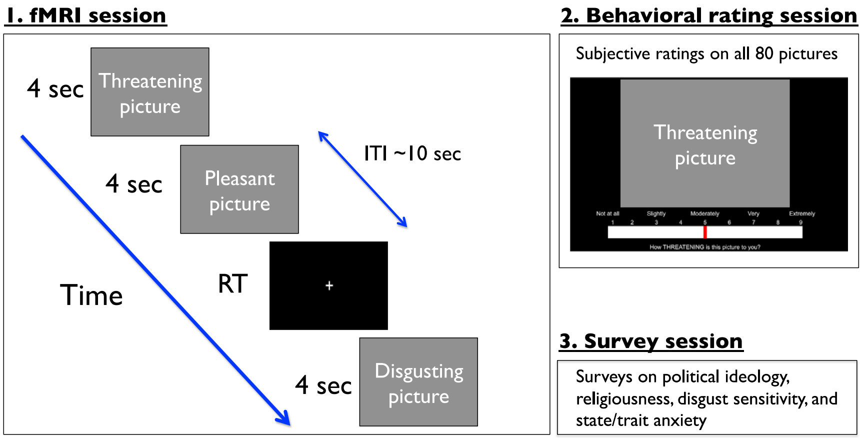

Figure 1. Time Course of the Experiment

Each subject first participated in an affective picture-viewing task in the fMRI scanner, during which they viewed 80 color pictures (20 disgusting, 20 threatening, 20 pleasant, and 20 neutral pictures). Occasionally, a fixation cross appeared on the screen, and participants were asked to press a button as soon as they saw the cross. Each picture was presented for 4 s, and the fixation cross was presented until participants pressed a button. The mean intertrial interval (ITI) was 10 s. Next, participants completed a behavioral rating session and several computerized surveys (see the Supplemental Experimental Procedures). See also Figure S1.

Experimental Procedures). The elastic net algorithm offers several advantages for fMRI data, including automatic variable selection (i.e., regression coefficients of unimportant variables [voxels] shrink to zero) and model regularization, which increases the interpretability of the findings. The elastic net also enjoys a grouping effect, which clusters highly correlated predictors into a set of groups. The grouping effect is useful for fMRI data because they contain many correlated predictors (voxels) due to its inherent nature (i.e., a brain region may consist of many voxels) and spatial smoothing, which is a commonly used preprocessing step. Previous studies demonstrated that the elastic net performs better than least absolute shrinkage and selection operator (LASSO), especially when the number of predictors is much higher than the number of observations [18]. The elastic net is beginning to be applied to fMRI data [19, 20] and appears to be a promising tool for developing predictive models from neuroimaging (and other types of) data. Using the elastic net algorithm (penalized logistic regression analysis) and contrast maps ([disgusting > neutral], [threatening > neutral], or [pleasant > neutral]), we probed brain regions critical for cross-validated classification accuracy (liberal versus conservative groups; see the Supplemental Experimental Procedures).

Figure 3A shows a network of brain regions predicting conservative and liberal group membership revealed by the machine-learning method with the [disgusting > neutral] contrast. Separate tests for the out-of-sample performance confirmed the robustness of the findings (Figure 3B and the Supplemental Experimental Procedures). No voxel survived cross-validations on other contrasts. Red-to-yellow and blue-to-green regions indicate voxels predicting conservative and liberal groups, respectively. As seen in Figure 3A, conservative group membership was predicted by increases in the basal ganglia (peak MNI = [16, 8, −8], k = 234)/thalamus (peak MNI = [20, −18, 6])/periaqueductal gray (PAG; peak MNI = [10, −24, −12]/hippocampus (peak MNI = [−14, −4, −14])/amygdala (peak MNI = [−18, −4, −14]), dorsolateral prefrontal cortex (DLPFC; peak MNI = [−44, 4, 52], k = 26), middle/superior temporal gyrus (MTG/STG; peak MNI = [−60, −44, 6], k = 33), presupplementary motor area (pre-SMA; peak MNI = [−4, 8, 56], k = 56), fusiform gyrus (FFG; peak MNI = [−42, −52, −10], k = 24 in the left side and [42, −60, −10], k = 16 in the right side), and inferior frontal gyrus (IFG; peak MNI = [52, 28, 4], k = 15). The increase in the secondary somatosensory cortex (S2)/posterior insula (peak MNI = [−40, −26, 19])/inferior parietal lobule (IPL; peak MNI = [−48, −40, 36], k = 125 in the left side and [48, −52, 54], k = 17 in the right side), frontal insula (MNI = [40, 16, −12], k = 19), and precentral gyrus (peak MNI = [−38, −12, 50], k = 25 in the left sid and [40, −12, 52], k = 13 in the right side) predicted liberal group membership. Note that the group differences using the traditional general linear modeling (GLM) revealed similar findings with some differences (Figure S2D, Table S4, and the Supplemental Experimental Procedures). The mean area under the curve (AUC) of the receiver-operating characteristic (ROC) curve was 0.981 (SD = 0.043). See the Supplemental Experimental Procedures and Figure S4 for more details and additional results using penalized linear regression across all participants. When we examined the prediction accuracy of each disgust subcondition (core/contamination and animal reminder), only animal-reminder disgust (e.g., mutilated body) was a strong predictor of political attitudes (Figure 3C; mean AUC = 0.998, SD = 0.003 for animal reminder; mean AUC = 0.548, SD = 0.125 for core/contamination).

Recent work suggests that BOLD time-series data from a single stimulus can categorically differentiate healthy individuals from those diagnosed with autism spectrum disorder (unpublished data). Lu et al. applied a machine-learning approach to time-series data from a specific region of interest and demonstrated that single-stimulus brain responses to a specific kind of stimulus could be used to make accurate categorical predictions of disorder status. We tested the hypothesis that a single-stimulus measurement combined with a machine-learning approach may contain enough information to predict liberal and conservative group membership per individual participant. Following Lu et al., we extracted the entire BOLD time-series response to the first disgusting picture. Time-series data every 2 s were spatially averaged within each of two types of patterns shown in Figure 3A: (+) voxels (red-to-yellow regions predicting conservative group) and (−) voxels (blue-to-green regions predicting liberal group) (see the Supplemental Experimental Procedures).

As seen in Figure 4A, the single-stimulus presentation of the disgusting pictures reliably differentiated the conservative and liberal groups in the (+) voxels. The hemodynamic response of the conservative group had a steeper slope and a higher peak than that of the liberal group. The mean AUC of the ROC curve using the single-stimulus presentation was 0.845 (SD = 0.009; Figure 4B). When we used each region of interest within the (+) voxels for the same analysis (Figure 4C), the thalamus (mean AUC = 0.816, SD = 0.023) and the DLPFC (mean AUC = 0.807, SD = 0.018) were the strongest predictive regions,

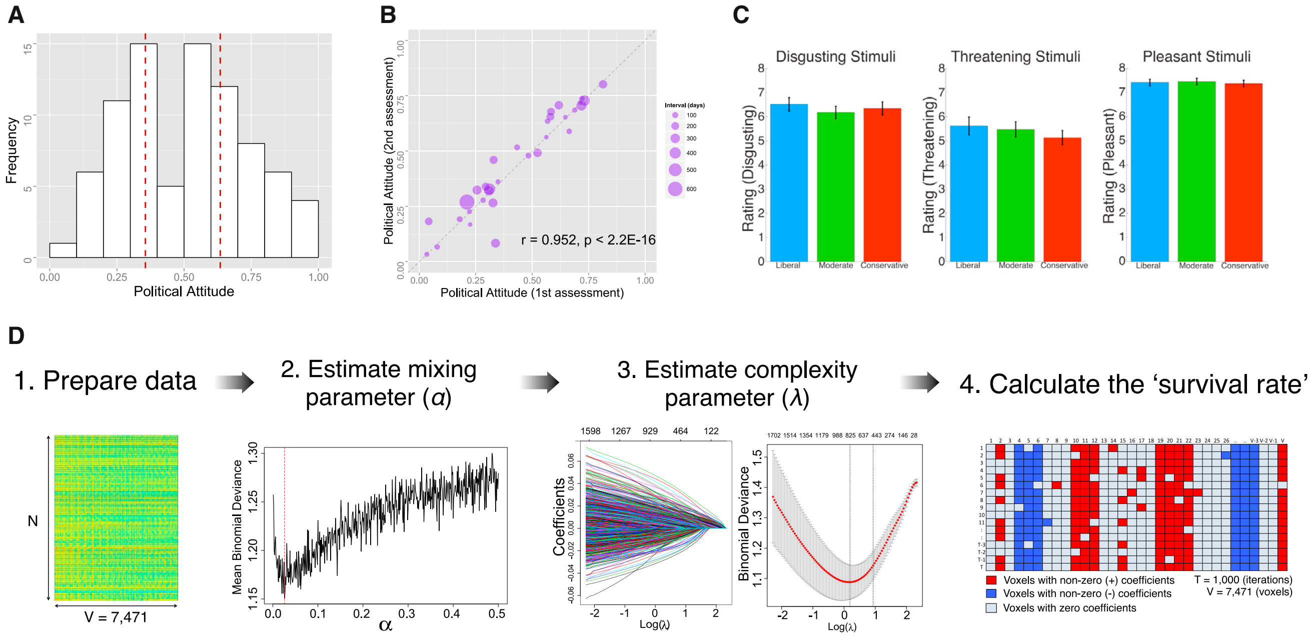

Figure 2. Behavioral Results and an Illustration of Workflow for Penalized Regression Analysis

(A) Distribution of political attitudes (orientation). Political attitudes are scaled from 0 (extremely liberal) to 1 (extremely conservative) (mean = 0.500, SD = 0.225). We divided participants (n = 83) into three groups (liberal [n = 28], moderate [n = 27], and conservative [n = 28]) based on their political attitudes. Red dotted lines indicate tertiles (33.3% and 66.6%).

(B) Test-retest reliability of political attitudes. The Pearson correlation coefficient is 0.952, $p < 2.2 \times 10^{-16}$, and the robust correlation coefficient is 0.986, $p < 2.0 \times 10^{-16}$.

(C) Subjective ratings of emotional pictures for each group. Error bars indicate ±1 SE.

(D) Schematic illustration of workflow for a machine-learning (penalized-regression) model. A 10-fold cross-validation is used to estimate two tuning parameters of the elastic net model. The survival rate was projected back into the brain space (see the Supplemental Experimental Procedures and Figure S3A). See also Figure S3.

followed by the basal ganglia (mean AUC = 0.789, SD = 0.005), FFG (mean AUC = 0.764, SD = 0.047), pre-SMA (mean AUC = 0.733, SD = 0.044), amygdala/hippocampus (mean AUC = 0.721, SD = 0.079), PAG (mean AUC = 0.662, SD = 0.100), and MTG/STG (mean AUC = 0.654, SD = 0.105). While increase in the (−) voxels predicted liberal group membership with full data, none of the BOLD time-series data from the (−) voxels survived using the single-stimulus analysis.

Discussion

Neuroscience has started to provide rich information about the neurophysiological processes underlying political behavior. Our results have important implications for the links between biology, emotions, political ideology, and human nature more fundamentally. While previous studies using skin conductance response [9–11], neuroimaging [21–24], and questionnaire [25, 26] measures suggested the role of emotions in political attitudes, to our knowledge, this is the first fMRI study revealing multivariate patterns of brain activity that differ between liberals and conservatives during emotional processing of sensory stimuli. Accumulating evidence suggests that cognition and emotion are deeply intertwined [27], and a view of segregating cognition and emotion is becoming obsolete [28]. People tend to think that their political views are purely cognitive (i.e., rational). However, our results further support the notion that emotional processes are tightly coupled to complex and high-dimensional human belief systems [13], and such emotional processes might play a much larger role than we currently believe, possibly outside our awareness of its influence [29]. Despite growing evidence from various fields, including genetics, cognitive neuroscience, and psychology, many political scientists remain skeptical of research connecting biological factors with political ideology, arguing variously that biology is irrelevant to central political questions [30], that the theoretical basis for expecting biology to be relevant is weak and murky [31], that acknowledging a role for biology is reductionist [32], and that recognizing the relevance of biology to human beliefs and behaviors is potentially dangerous [33]. We hope some of this skepticism can be alleviated from our demonstration that fMRI data, even from a single stimulus, can serve as a strong predictor of political ideology.

Several groups have suggested that people are born with certain dispositions and traits that influence the formation of their political beliefs [3, 4]. Also, several studies have shown that life history (e.g., [34]) and traumatic experiences [35] can affect political views. Our results are consistent with the idea that political beliefs are connected to neurobiological composition. But both genetics and life history play an important role in establishing both connections between neuroanatomical regions and the propensity for these regions to respond to environmental stimuli. We have not isolated the distinct roles played by genetics and life history in the development of the brain responses that we measured.

A wide range of brain regions contributed to the prediction of political ideology (Figure 3A), including those known from past work to be involved in the processing and interoception of disgust and other stimuli with negative affective valence, but also those involved in more basic aspects of attentive

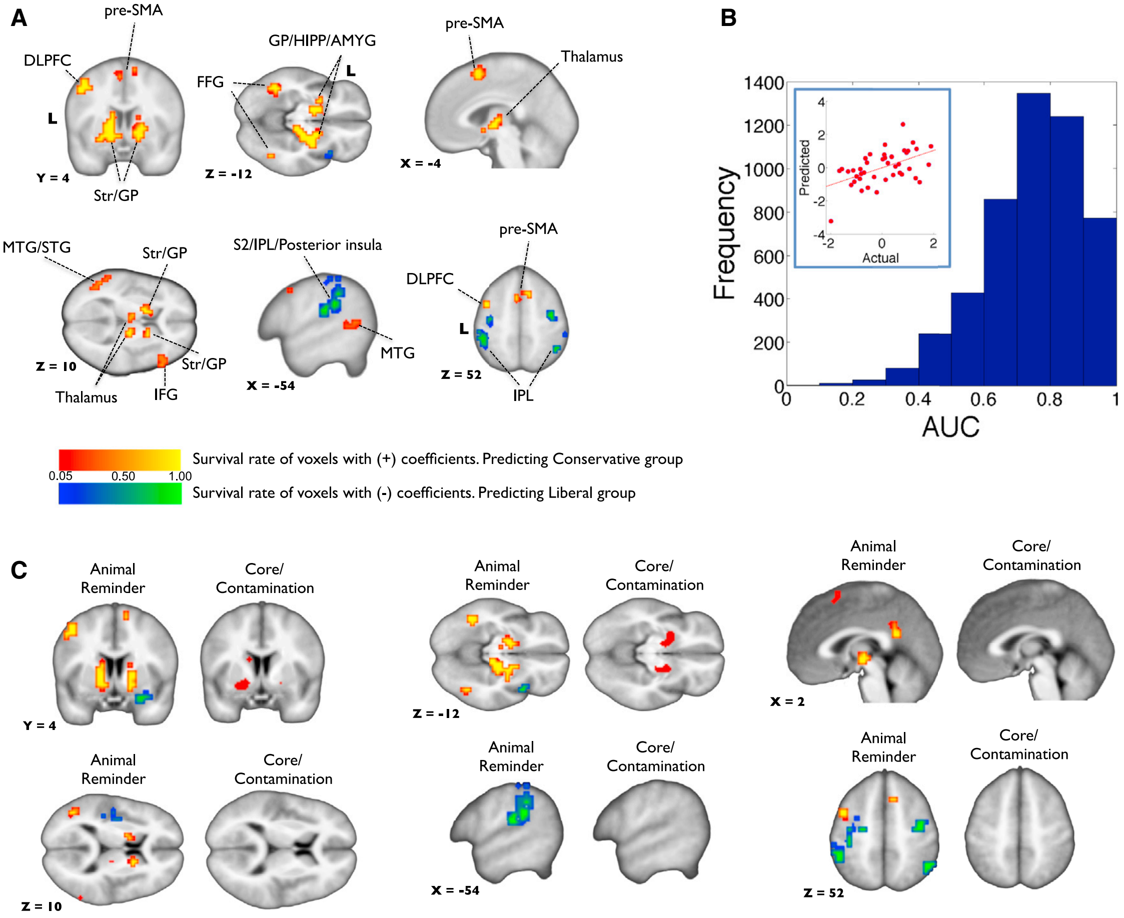

Figure 3. Multivariate Patterns of Brain Activity that Predict Political Ideology

(A) Voxels predicting conservative (red-to-yellow) or liberal (blue-to-green) group membership from penalized logistic regression analysis (cluster size, k $\geq$ 10). Survival rate is closely related to voxel (regression) weights (see Figure S3B). DLPFC, dorsolateral prefrontal cortex; pre-SMA, presupplementary motor area; Str, striatum; GP, globus pallidus; HIPP, hippocampus; AMYG, amygdala; MTG/STG, middle/superior temporal gyrus; IFG, inferior frontal gyrus; S2, secondary somatosensory cortex; IPL, inferior parietal lobule; and FFG, fusiform gyrus. The color scale denotes the survival rate.

(B) Distribution of cross-validated area under the curve (AUC). We ran 1,000 iterations of 5-fold cross-validation procedure. For each iteration, we first found the λ that minimized the out-of-sample binomial deviance of four folds (80% of the data). Then, for each of the five folds, we computed the area under the receiver-operating characteristic (ROC) curve using predictions from the model fit to the remaining data using the minimum λ. This resulted in the 5,000 (1,000 iterations $\times$ 5 AUCs per iteration) AUC calculations plotted in the histogram (mean = 0.757, median = 0.771, mode = 0.833, SD = 0.150). The inset in the top-left corner shows out-of-sample prediction performance on the half of the data (test set) when the model is trained on the other half of the data (training set) for penalized linear regression. The x and y axes show the *Z* scores of actual political attitudes and predicted political attitudes from BOLD signals, respectively. Pearson correlation coefficient = 0.52, p = 0.0004; robust correlation coefficient = 0.44, p = 0.0024. See the Supplemental Experimental Procedures for complete details.

(C) Voxels predicting conservative or liberal group membership from each subcondition of disgust (i.e., using contrast maps of [animal-reminder disgust > neutral] or [core/contamination disgust > neutral]; see the Supplemental Experimental Procedures for the details of subconditions). The voxel survival criterion is the same as that for (A).

See also Figure S2.

sensory processing: we found regions known to be involved in disgust recognition [17, 36–38] (e.g., insula, basal ganglia, and amygdala), perception of bodily signals [39] (e.g., insula), the experience of physical/social pain [40] or observing others in pain [41] (e.g., S2, insula, PAG, and thalamus), and emotion regulation [42] (e.g., DLPFC, insula, amygdala, and pre-SMA), along with regions involved in information integration [43] (e.g., thalamus and amygdala), attention [43, 44] (e.g., amygdala, IPL, FFG, STG/MTG), memory retrieval [44, 45] (e.g., hippocampus, amygdala, and IPL), and also inhibitory control [46] (e.g., IFG, DLPFC, and pre-SMA), perhaps to suppress innate responses. Although our results suggest that disgusting pictures evoke very different emotional processing in conservatives and liberals, it will take a range of targeted studies in the future to tease apart the separate contribution of each brain circuit.

We proposed that conservatives, compared to liberals, have greater negativity bias [13], which includes both disgusting and threatening conditions in our study. Our finding that only disgusting pictures, especially in the animal-reminder category, differentiate conservatives from liberals might be indicative of a primacy for disgust in the pantheon of human aversions, but it is also possible that this result is due to the fact that, compared to threat, disgust is much easier to evoke with visual images on a computer screen.

Lastly, this study raises several important but unaddressed questions. First, while political ideology has effects on many forms of behavior (including, but not limited to, voting

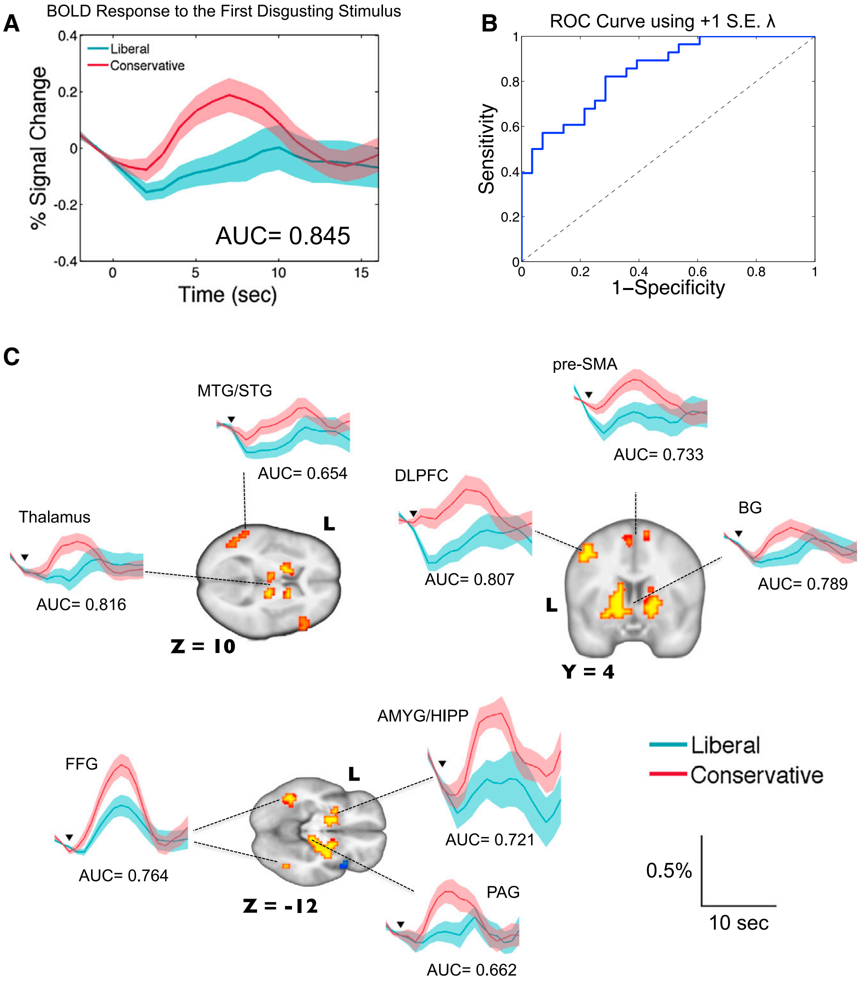

Figure 4. Single Presentation of a Disgusting Stimulus Is Sufficient to Make Accurate Predictions of Individual's Political Orientation

(A) Hemodynamic response to the first disgusting stimulus for the liberal and conservative groups, extracted from the red-to-yellow voxels in Figure 3A. Shaded regions indicate ±1 SE. Time-series data were linearly interpolated every 1 s for display purposes. "AUC" indicates the mean AUC of ROC curves over 1,000 iterations.

(B) A representative ROC curve.

(C) Hemodynamic response to the first disgusting stimulus, extracted from each predictive region, as well as the mean AUCs of the corresponding ROC curves. The x axis is time since stimulus presentation (s) and the y axis is the percent signal change (percentage). Black inverse triangles indicate the stimulus onset, the bottom of which is at 0.05% signal change. DLPFC, dorsolateral prefrontal cortex; pre-SMA, presupplementary motor area; BG, basal ganglia; AMYG/HIPP, amygdala/hippocampus; MTG/STG, middle/superior temporal gyrus; FFG, fusiform gyrus; and PAG, periaqueductal gray.

See also Figure S4.

behavior), it is unknown whether it does so thanks to the neural differences in affective processing that we measured. Second, and relatedly, it is important also to know how individual differences in the capacity to regulate emotion [26], and the neural bases of that capacity, are related to political ideology. A third set of questions concerns the bearing of the present study on the development of biological measures of political ideology. While it is of use in a variety of settings to measure political ideology (political polls, for instance, typically include some measurement of it), it remains an open question whether biological measures could become more accurate, or more useful, than the tools (such as self-report measures) currently employed. Determining the answer to that question would require answering a host of others: How would a machine-learning model based on data collected in one region (e.g., New York) support predictions of people's political attitudes in another region (e.g., Texas)? How fine-grained are the categories of affective response that are tied to political ideology? Although our results show greater differentiation in political ideology in cases of animal-reminder disgust than core/contamination disgust, what are the links between political ideology and other forms of disgust, such as moral disgust? The more we learn about the sensitivity of political ideology to subtle differences in affective response and their neural bases, the more we will know about the feasibility of useful and portable tools for ideology's biological measurement. This would then raise a further and difficult ethical question about the circumstances, if any, in which it is appropriate to use such tools. And, finally, the present study raises important questions about the possibility of, and obstacles to, understanding and cooperation across divides in political ideology. Would the recognition that those with different political beliefs from our own also exhibit different disgust responses from our own help us or hinder us in our ability to embrace them as coequals in democratic governance? Future work will be necessary to answer these important questions.

Experimental Procedures

Participants

Eighty-three healthy individuals (males/females = 41/42; age = 18–62; mean [SD] = 29.0 [11.3] years) in Roanoke and Blacksburg, VA, area were recruited from a large database maintained by the Human Neuroimaging Laboratory between September 2012 and September 2013. See the Supplemental Experimental Procedures for inclusion/exclusion criteria for participants and demographic data.

fMRI Task

Participants were informed that they would complete a simple visual perception task. They were told to simply look at emotional pictures when they were presented but to press a button when they saw a fixation cross. Figure 1 depicts the time course of the fMRI experiment. It is a passive picture-viewing task presenting a total of 20 disgusting, 20 threatening, 20 pleasant, and 20 neutral pictures, the order of which was randomized for each participant. All the pictures were taken from the International Affective Picture System (IAPS) [14]. See Appendix S1 for IAPS picture numbers, description, and valence/arousal ratings of all pictures. Table S2 summarizes the mean IAPS valence and arousal ratings in each emotion condition. Each picture was presented for 4 s. Ten button-press (fixation-cross) trials were pseudorandomly mixed with emotional pictures to help participants stay fully awake and pay attention to visual stimuli. The fixation cross stayed on the screen until a button was pressed. Each trial was separated by a Poisson-distributed variable interval (mean = 10 s, SD = 10 s, minimum = 6 s, maximum = 17 s). The experiment took approximately 20 min in total.

NEMO (http://labs.vtc.vt.edu/hnl/nemo/index.html) was used for stimuli presentation and behavioral response collection.

MRI Data Acquisition and Analysis

The anatomical and functional imaging sessions were conducted on a 3.0 tesla Siemens Magnetom Trio scanner at VTCRI. We used SPM8 (http://www.fil.ion.ucl.ac.uk/spm/software/spm8/) for preprocessing and standard GLM fMRI analyses. For the elastic net analysis, we used the *glmnet* package for MATLAB (http://web.stanford.edu/~hastie/glmnet_matlab/) and R [47]. See the Supplemental Experimental Procedures for complete details.

Supplemental Information

Supplemental Information includes Supplemental Experimental Procedures, four figures, four tables, and two appendices and can be found with this article online at http://dx.doi.org/10.1016/j.cub.2014.09.050.

Author Contributions

W.-Y.A., X.G., T.L., A.H., J.R.A., K.B.S., J.R.H., and P.R.M. conceived and designed the experiments; W.-Y.A. performed the research; W.-Y.A. and T.L. analyzed the data; P.R.M. supervised the project; and all authors wrote the paper.

Acknowledgments

This work was funded by the Wellcome Trust Principal Research Fellowship (P.R.M.), the Gatsby Charitable Trust (P.D.), the National Science Foundation (J.R.H., K.B.S., J.R.A., and P.R.M.), and the Kane Family Foundation (P.R.M.). The authors thank Sébastien Hétu, Jacob I. Lee, and Iris Vilares for their helpful comments on a previous version of the manuscript.

Received: May 30, 2014
Revised: August 29, 2014
Accepted: September 19, 2014
Published: October 30, 2014

References

1. Jost, J.T., Federico, C.M., and Napier, J.L. (2009). Political ideology: its structure, functions, and elective affinities. Annu. Rev. Psychol. *60*, 307–337.
2. Haidt, J. (2012). The Righteous Mind: Why Good People Are Divided by Religion and Politics (New York: Pantheon).
3. Fowler, J.H., Baker, L.A., and Dawes, C.T. (2008). Genetic variation in political participation. Am. Polit. Sci. Rev. *102*, 233–248.
4. Alford, J.R., Funk, C.L., and Hibbing, J.R. (2005). Are political orientations genetically transmitted? Am. Polit. Sci. Rev. *99*, 153–167.
5. Hatemi, P.K., Gillespie, N.A., Eaves, L.J., Maher, B.S., Webb, B.T., Heath, A.C., Medland, S.E., Smyth, D.C., Beeby, H.N., Gordon, S.D., et al. (2011). A genome-wide analysis of liberal and conservative political attitudes. J. Polit. *73*, 271–285.
6. Settle, J.E., Dawes, C.T., and Fowler, J.H. (2009). The heritability of partisan attachment. Polit. Res. Q. *62*, 601–613.
7. Kanai, R., Feilden, T., Firth, C., and Rees, G. (2011). Political orientations are correlated with brain structure in young adults. Curr. Biol. *21*, 677–680.
8. Amodio, D.M., Jost, J.T., Master, S.L., and Yee, C.M. (2007). Neurocognitive correlates of liberalism and conservatism. Nat. Neurosci. *10*, 1246–1247.
9. Oxley, D.R., Smith, K.B., Alford, J.R., Hibbing, M.V., Miller, J.L., Scalora, M., Hatemi, P.K., and Hibbing, J.R. (2008). Political attitudes vary with physiological traits. Science *321*, 1667–1670.
10. Dodd, M.D., Balzer, A., Jacobs, C.M., Gruszczynski, M.W., Smith, K.B., and Hibbing, J.R. (2012). The political left rolls with the good and the political right confronts the bad: connecting physiology and cognition to preferences. Philos. Trans. R. Soc. Lond. B Biol. Sci. *367*, 640–649.
11. Smith, K.B., Oxley, D., Hibbing, M.V., Alford, J.R., and Hibbing, J.R. (2011). Disgust sensitivity and the neurophysiology of left-right political orientations. PLoS ONE *6*, e25552.
12. Schaller, M., and Park, J.H. (2011). The behavioral immune system (and why it matters). Curr. Dir. Psychol. Sci. *20*, 99–103.
13. Hibbing, J.R., Smith, K.B., and Alford, J.R. (2014). Differences in negativity bias underlie variations in political ideology. Behav. Brain Sci. *37*, 297–307.
14. Lang, P.J., Bradley, M.M., and Cuthbert, B.N. (2008). International affective picture system (IAPS): affective ratings of pictures and instruction manual. Technical Report A-8 (Gainesville: University of Florida).
15. Wilson, G.D., and Patterson, J.R. (1968). A new measure of conservatism. Br. J. Soc. Clin. Psychol. *7*, 264–269.
16. Winkielman, P., and Berridge, K.C. (2004). Unconscious emotion. Curr. Dir. Psychol. Sci. *13*, 120–123.
17. Suzuki, A., Hoshino, T., Shigemasu, K., and Kawamura, M. (2006). Disgust-specific impairment of facial expression recognition in Parkinson's disease. Brain *129*, 707–717.
18. Zou, H., and Hastie, T. (2005). Regularization and variable selection via the elastic net. J. R. Stat. Soc., B *67*, 301–320.
19. Ryali, S., Supekar, K., Abrams, D.A., and Menon, V. (2010). Sparse logistic regression for whole-brain classification of fMRI data. Neuroimage *51*, 752–764.
20. Whelan, R., Watts, R., Orr, C.A., Althoff, R.R., Artiges, E., Banaschewski, T., Barker, G.J., Bokde, A.L.W., Büchel, C., Carvalho, F.M., et al.; IMAGEN Consortium (2014). Neuropsychosocial profiles of current and future adolescent alcohol misusers. Nature *512*, 185–189.
21. Westen, D., Blagov, P.S., Harenski, K., Kilts, C., and Hamann, S. (2006). Neural bases of motivated reasoning: an FMRI study of emotional constraints on partisan political judgment in the 2004 U.S. Presidential election. J. Cogn. Neurosci. *18*, 1947–1958.
22. Mitchell, J.P., Macrae, C.N., and Banaji, M.R. (2006). Dissociable medial prefrontal contributions to judgments of similar and dissimilar others. Neuron *50*, 655–663.
23. Tusche, A., Kahnt, T., Wisniewski, D., and Haynes, J.D. (2013). Automatic processing of political preferences in the human brain. Neuroimage *72*, 174–182.
24. Kaplan, J.T., Freedman, J., and Iacoboni, M. (2007). Us versus them: Political attitudes and party affiliation influence neural response to faces of presidential candidates. Neuropsychologia *45*, 55–64.
25. Inbar, Y., Pizarro, D., Iyer, R., and Haidt, J. (2012). Disgust sensitivity, political conservatism, and voting. Soc. Psychol. Pers. Sci. *3*, 537–544.
26. Lee, J.J., Sohn, Y., and Fowler, J.H. (2013). Emotion regulation as the foundation of political attitudes: does reappraisal decrease support for conservative policies? PLoS ONE *8*, e83143.
27. Gu, X., Liu, X., Van Dam, N.T., Hof, P.R., and Fan, J. (2013). Cognition-emotion integration in the anterior insular cortex. Cereb. Cortex *23*, 20–27.
28. Pessoa, L. (2008). On the relationship between emotion and cognition. Nat. Rev. Neurosci. *9*, 148–158.
29. Westen, D. (2008). Political Brain (New York: Public Affairs).
30. Schlozman, K.L. (2013). Two concerns about ten misconceptions. Perspect. Polit. *11*, 490–491.
31. Engelmann, S.G. (2010). Theory trouble: the case of biopolitical science. Aust. J. Polit. Sci. *1*, 55–71.
32. Duster, T. (2013). Emergence vs. reductionism in the debate over the role of biology in politics. Perspect. Polit. *11*, 495–499.
33. Charney, E. (2008). Genes and ideologies. Perspect. Polit. *6*, 299–319.
34. Ghitza, Y. and Gelman, A. (2014). The great society, Reagan's revolution, and generations of presidential voting. http://www.stat.columbia.edu/~gelman/research/unpublished/cohort_voting_20140605.pdf.
35. Carmil, D., and Breznitz, S. (1991). Personal trauma and world view—are extremely stressful experiences related to political attitudes, religious beliefs, and future orientation? J. Trauma. Stress *4*, 393–405.
36. Phillips, M.L., Young, A.W., Senior, C., Brammer, M., Andrew, C., Calder, A.J., Bullmore, E.T., Perrett, D.I., Rowland, D., Williams, S.C., et al. (1997). A specific neural substrate for perceiving facial expressions of disgust. Nature *389*, 495–498.
37. Sprengelmeyer, R., Rausch, M., Eysel, U.T., and Przuntek, H. (1998). Neural structures associated with recognition of facial expressions of basic emotions. Proc. Biol. Sci. *265*, 1927–1931.
38. Sprengelmeyer, R., Young, A.W., Calder, A.J., Karnat, A., Lange, H., Hömberg, V., Perrett, D.I., and Rowland, D. (1996). Loss of disgust. Perception of faces and emotions in Huntington's disease. Brain *119*, 1647–1665.
39. Craig, A.D. (2003). Interoception: the sense of the physiological condition of the body. Curr. Opin. Neurobiol. *13*, 500–505.

40. Wager, T.D., Atlas, L.Y., Lindquist, M.A., Roy, M., Woo, C.-W., and Kross, E. (2013). An fMRI-based neurologic signature of physical pain. N. Engl. J. Med. *368*, 1388–1397.
41. Keysers, C., Kaas, J.H., and Gazzola, V. (2010). Somatosensation in social perception. Nat. Rev. Neurosci. *11*, 417–428.
42. Wager, T.D., Davidson, M.L., Hughes, B.L., Lindquist, M.A., and Ochsner, K.N. (2008). Prefrontal-subcortical pathways mediating successful emotion regulation. Neuron *59*, 1037–1050.
43. Pessoa, L., and Adolphs, R. (2010). Emotion processing and the amygdala: from a 'low road' to 'many roads' of evaluating biological significance. Nat. Rev. Neurosci. *11*, 773–783.
44. Hutchinson, J.B., Uncapher, M.R., and Wagner, A.D. (2009). Posterior parietal cortex and episodic retrieval: convergent and divergent effects of attention and memory. Learn. Mem. *16*, 343–356.
45. Phelps, E.A. (2004). Human emotion and memory: interactions of the amygdala and hippocampal complex. Curr. Opin. Neurobiol. *14*, 198–202.
46. Aron, A.R. (2011). From reactive to proactive and selective control: developing a richer model for stopping inappropriate responses. Biol. Psychiatry *69*, e55–e68.
47. Friedman, J., Hastie, T., and Tibshirani, R. (2010). Regularization paths for generalized linear models via coordinate descent. J. Stat. Softw. *33*, 1–22.

Current Biology, Volume *24*

Supplemental Information

Nonpolitical Images Evoke Neural Predictors of Political Ideology

Woo-Young Ahn, Kenneth T. Kishida, Xiaosi Gu, Terry Lohrenz, Ann Harvey, John R. Alford, Kevin B. Smith, Gideon Yaffe, John R. Hibbing, Peter Dayan, and P. Read Montague

Figure S1

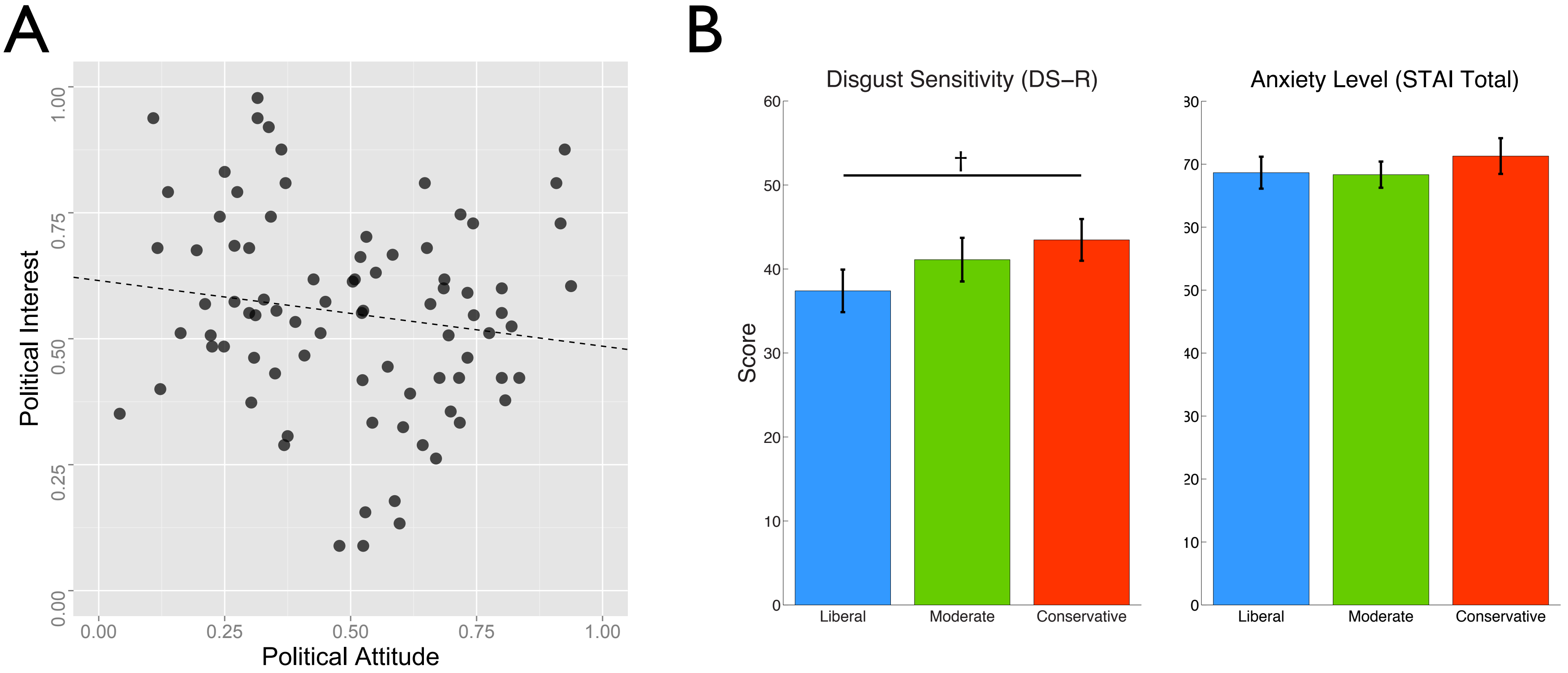
A
Political Interest
Political Attitude
0.00
0.25
0.50
0.75
1.00
B
Disgust Sensitivity (DS–R)
Anxiety Level (STAI Total)
Score
†
60
50
40
30
20
10
0
70
Liberal
Moderate
Conservative

Figure S2

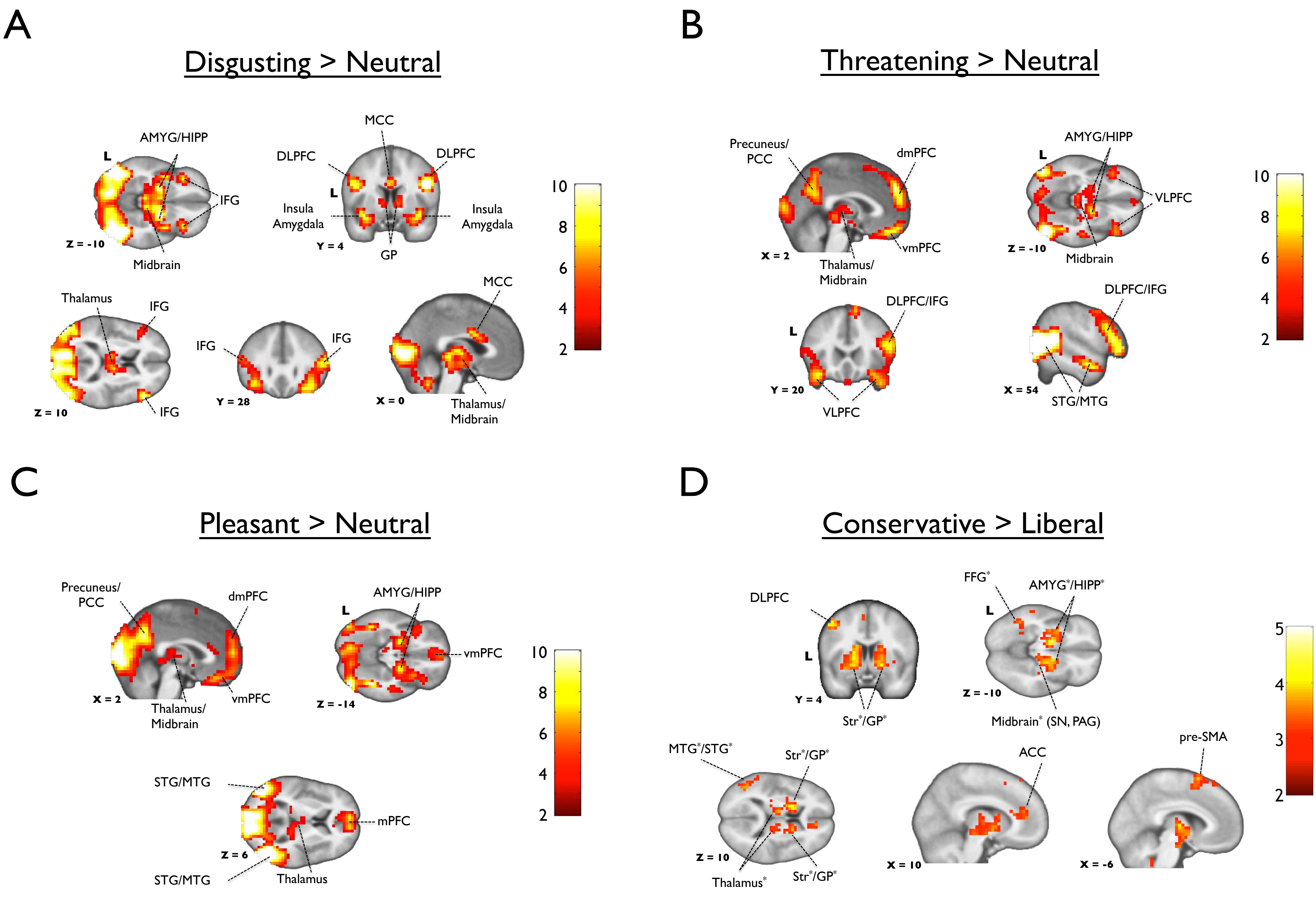
A
Disgusting > Neutral
AMYG/HIPP
L
IFG
Z = -10
Midbrain
MCC
DLPFC
DLPFC
L
Insula
Amygdala
Insula
Amygdala
Y = 4
GP
Thalamus
IFG
Z = 10
IFG
IFG
IFG
Y = 28
MCC
X = 0
Thalamus/
Midbrain
10
8
6
4
2
B
Threatening > Neutral
Precuneus/
PCC
dmPFC
X = 2
Thalamus/
Midbrain
vmPFC
AMYG/HIPP
L
VLPFC
Z = -10
Midbrain
DLPFC/IFG
L
Y = 20
VLPFC
DLPFC/IFG
X = 54
STG/MTG
10
8
6
4
2
C
Pleasant > Neutral
Precuneus/
PCC
dmPFC
X = 2
Thalamus/
Midbrain
vmPFC
AMYG/HIPP
L
vmPFC
Z = -14
STG/MTG
mPFC
Z = 6
STG/MTG
Thalamus
10
8
6
4
2
D
Conservative > Liberal
DLPFC
L
Y = 4
Str*/GP*
FFG*
AMYG*/HIPP*
L
Z = -10
Midbrain* (SN, PAG)
MTG*/STG*
Str*/GP*
Z = 10
Thalamus*
Str*/GP*
ACC
X = 10
pre-SMA
X = -6
5
4
3
2

Figure S3

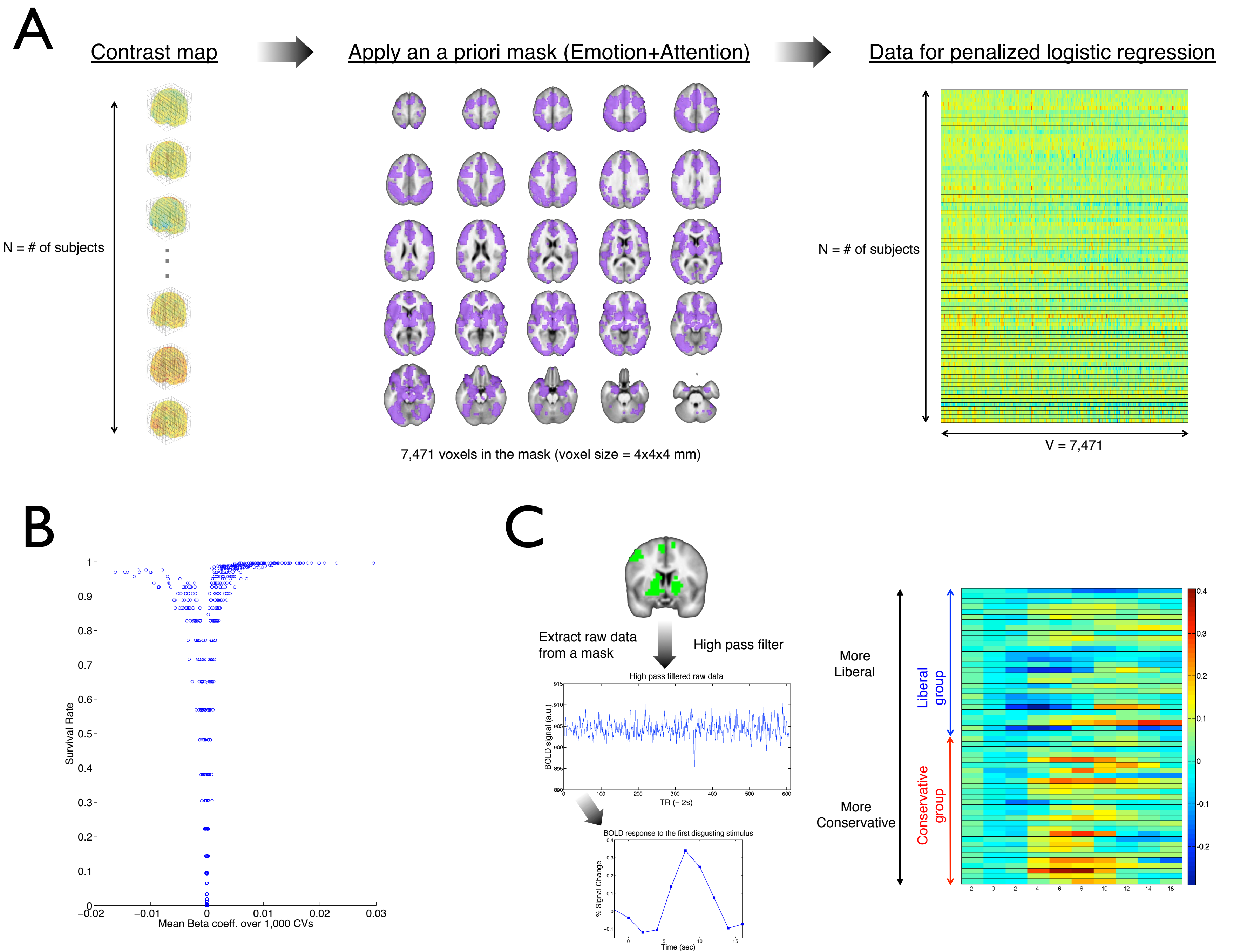
A
Contrast map
Apply an a priori mask (Emotion+Attention)
Data for penalized logistic regression
N = # of subjects
7,471 voxels in the mask (voxel size = 4x4x4 mm)
N = # of subjects
V = 7,471
B
Survival Rate
Mean Beta coeff. over 1,000 CVs
C
Extract raw data from a mask
High pass filter
High pass filtered raw data
BOLD signal (a.u.)
TR (= 2s)
BOLD response to the first disgusting stimulus
% Signal Change
Time (sec)
More Liberal
More Conservative
Liberal group
Conservative group

Figure S4

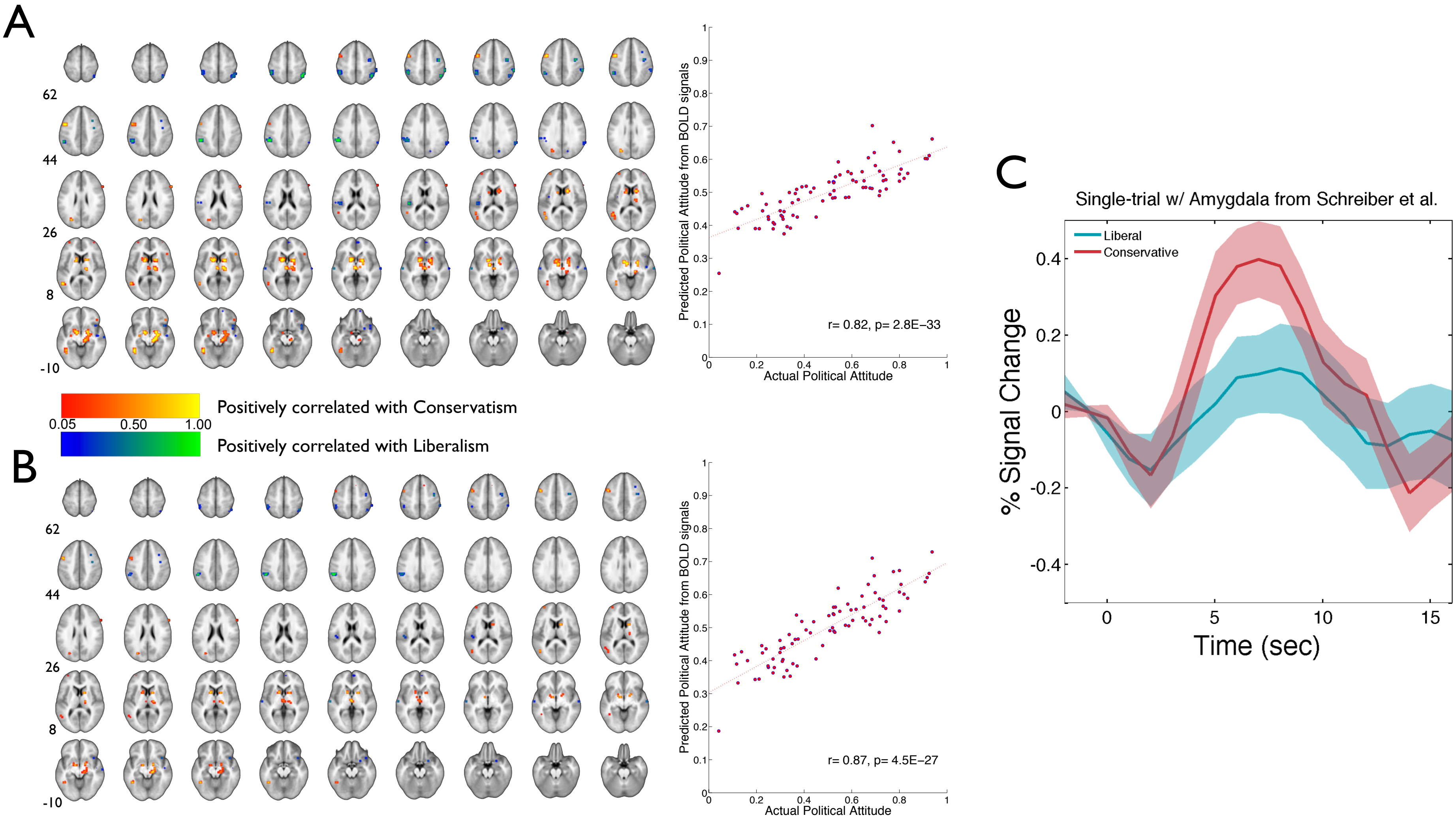
A
62
44
26
8
-10
0.05
0.50
1.00
Positively correlated with Conservatism
Positively correlated with Liberalism
B
Predicted Political Attitude from BOLD signals
Actual Political Attitude
r= 0.82, p= 2.8E−33
r= 0.87, p= 4.5E−27
C
Single-trial w/ Amygdala from Schreiber et al.
Liberal
Conservative
% Signal Change
Time (sec)

Supplemental Figure Legends

Figure S1: Related to Figure 1. (A) The relationship between political attitudes and political interest. A dashed line is the linear regression fit ($r = -0.148$, $p = 0.182$) (B) Disgust Sensitivity (DS-R) and Anxiety Level (STAI Total) survey scores. The Liberal group reported marginally lower disgust sensitivity than the Conservative group. Error bars indicate ±1 S.E.

Figure S2: Related to Figure 3. (A, B, C) The contrasts of [Disgusting > Neutral], [Threatening > Neutral], and [Pleasant > Neutral] stimuli using the standard General Linear Model (GLM) analysis across all participants (n=83). All maps are at $p < 0.001$, whole-brain family-wise error (FWE) corrected at the cluster level. (D) The comparison of Conservative and Liberal groups using standard General Linear Model (GLM) analysis for the [Disgusting > Neutral] contrast. All maps are at $p < 0.005$ (uncorrected, cluster size > 10). Regions with * marks survived the FWE cluster-level correction. AMYG, amygdala; HIPP, hippocampus; IFG, inferior frontal gyrus; MCC, middle cingulate cortex; DLPFC, dorsolateral prefrontal cortex; GP, globus pallidus; PCC, posterior cingulate cortex; vmPFC, ventromedial prefrontal cortex; dmPFC, dorsomedial prefrontal cortex; VLPFC, ventrolateral prefrontal cortex; STG/MTG, superior/middle temporal gyrus; Str, striatum; SN, substantia nigra; PAG, periaqueductal gray; ACC, anterior cingulate cortex; pre-SMA, pre-supplementary motor area; FFG, fusiform gyrus. Color scale denotes t-scores.

Figure S3: Related to Figure 2. (A) Preparation of data for the full-data penalized regression analysis. First, prepare a map of the contrast of interest (e.g., [Disgust > Neutral]) for each participant. Second, apply an a priori mask generated from the Neurosynth website (neurosynth.org). We used the union of activations from "Emotion" and "Attention" terms (including both forward and reverse inference maps). Third, generate a matrix (number of subjects x number of voxels) for a penalized (logistic or linear) regression analysis. (B) An illustration of the relationship between survival rates and regression coefficients. The plot shows that higher survival rates are associated with greater magnitudes of beta coefficients in penalized logistic regression analysis. Each dot indicates each voxel within the mask (total number of voxels = 7,471). (C) Preparation of data for the single-stimulus fMRI (penalized logistic regression) analysis. (A) In each participant, extract the raw time series data that are spatially averaged within the red-to-yellow voxels (**Figure 3A**) or a region of interest, and then apply a high pass filter to the data, which are then linearly interpolated every 1s. Extract an epoch of [-2s, 16s] (indicated by red dashed lines) every 2s from the onset of the first disgusting picture (11 time points for each participant). (B) Generate a matrix (number of participants x number of time points) for the single-stimulus fMRI analysis. Data are sorted by political attitudes for display purposes. Labels on the x-axis indicate time since stimulus presentation (sec). Color scale denotes percent signal change.

Figure S4: Related to Figure 4. Voxels that are positively (red-to-yellow) or negatively (blue-to-green) correlated with political attitudes (using the [Disgusting > Neutral] contrast). We used penalized linear regression and all participants for generating the maps. The numbers on axial slices indicate MNI Z-axis coordinates. (A) Using α (mixing parameter) value optimized for penalized logistic regression ($\alpha = 0.026$). (B) Using α value optimized for penalized linear regression ($\alpha = 0.222$). Scatter plots on the right side indicate correlation plots between actual political attitudes and predicted political attitudes from BOLD data across all participants. Color scale denotes survival rate. (C) Hemodynamic response to the first disgusting stimulus, extracted from an independent amygdala ROI from [S1]. Shaded regions indicate ±1 S.E. See **Supplemental Experimental Procedure** for complete details.

Table S1. Demographic/survey data and IAPS ratings of the first disgusting stimuli for the Liberal, Moderate, and Conservative groups. Values indicate means and standard deviations (in parentheses) except for Sex. C/A = Core/Contamination versus Animal Reminder (disgust).

	Liberal (n=28)	Moderate (n=27)	Conservative (n=28)
Age	32.7 (15.5)	27.6 (9.2)	26.8 (11.2)
Sex (Male/Female)	17/11	13/14	11/17
Political orientation	0.25 (0.08)	0.50 (0.08)	0.75 (0.09)
Political interest	0.63 (0.18)	0.46 (0.22)	0.55 (0.16)
DS-R Total	37.39 (13.41)	41.11 (13.51)	43.46 (13.14)
STAI Total	68.64 (13.42)	68.33 (10.82)	71.29 (15.01)
IAPS valence rating of the first disgusting stimuli	2.30 (0.81)	2.17 (0.67)	2.22 (0.79)
IAPS arousal rating of the first disgusting stimuli	6.04 (1.20)	6.20 (0.93)	6.27 (0.76)
Onset of the first disgusting stimuli (sec)	30.96 (36.87)	45.33 (60.90)	50.57 (40.64)
Subcondition of the first disgusting stimuli (C/A)	14/14	13/14	12/16

Table S2. The mean and standard deviations (in parentheses) of IAPS valence and arousal ratings as well as behavioral ratings in each rating category.

	IAPS valence rating	IAPS arousal rating	Disgusting rating	Threatening rating	Pleasant rating
Disgusting stimuli	2.42 (0.82)	5.96 (0.99)	6.35 (1.39)	3.29 (1.90)	2.34 (0.62)
Threatening stimuli	2.79 (0.62)	6.28 (0.59)	3.15 (1.81)	5.40 (1.72)	2.84 (0.85)
Pleasant stimuli	7.87 (0.38)	4.62 (0.75)	1.08 (0.22)	1.11 (0.18)	7.42 (0.70)
Neutral stimuli	5.10 (0.39)	3.01 (0.72)	1.12 (0.29)	1.17 (0.35)	5.18 (0.34)

Table S3. Regions from the [Disgusting > Neutral], [Threatening > Neutral], and [Pleasant > Neutral] contrasts (Figure S2). Whole-brain family-wise error (FWE) corrected at the cluster level ($p < 0.05$). Height threshold, t = 3.19; extent thresholds = 44, 40, and 43 voxels (4x4x4 mm3). L: Left, R: Right.

Contrast	Region	Cluster size	Side	MNI coordinates			Peak T-score
				x	y	z	
Disgusting > Neutral	Dorsolateral prefrontal cortex	3445	L	-46	4	26	8.46
	Dorsolateral prefrontal cortex		R	46	4	26	12.64
	Middle occipital gyrus		L	-50	-76	-2	14.35
	Middle occipital gyrus		R	50	-72	-6	13.22
	Fusiform gyrus		L	-42	-52	-18	13.31
	Fusiform gyrus		R	42	-52	-18	13.19
	Middle temporal gyrus		L	-46	-76	10	8.37
	Middle temporal gyrus		R	46	-76	10	9.05
	Supramarginal gyrus		L	-62	-28	34	7.92
	Amygdala	1701	L	-22	-8	-14	13.86
	Amygdala		R	22	-4	-14	12.12
	Hippocampus		L	-22	-32	-2	7.63
	Hippocampus		R	33	-32	-2	8.69
	Globus pallidus/caudate		L	-10	4	2	4.88
	Globus pallidus/caudate		R	10	4	2	5.47
	Anterior insula		L	-34	24	2	5.53
	Anterior insula		R	38	28	-2	5.87
	Posterior insula		L	-42	-4	-2	4.94
	Posterior insula		R	42	-4	-2	5.73
	Inferior frontal gyrus		L	-50	32	18	5.71
	Inferior frontal gyrus		R	50	32	10	9.34
	Thalamus		L	-6	-16	6	7.79
	Thalamus		R	6	-12	2	8.56
	Midbrain (substantia nigra/periaqueductal gray)		L	-6	-28	-6	8.46
	Midbrain (substantia nigra/periaqueductal gray)		R	6	-28	-6	8.57
	Middle cingulate cortex	59	L	-2	0	30	7.20
	Supramarginal gyrus	54	R	62	-24	34	6.23
	Middle temporal gyrus	1394	L	-46	-76	14	13.08
	Middle temporal gyrus		R	50	-68	2	19.41
	Superior temporal gyrus		R	54	-52	10	15.34
	Fusiform gyrus		L	-42	-48	-18	12.69
	Fusiform gyrus		R	42	-48	-18	16.12

Threatening > Neutral	Dorsolateral prefrontal cortex/Inferior frontal gyrus	1266	L	-46	24	6	4.90
	Dorsolateral prefrontal cortex/Inferior frontal gyrus		R	54	24	14	8.90
	Amygdala		L	-18	-8	-14	5.69
	Amygdala		R	22	-4	-14	7.25
	Hippocampus		L	-22	-28	-6	4.53
	Hippocampus		R	26	-24	-10	4.14
	Inferior frontal gyrus		L	-38	20	-18	7.42
	Inferior frontal gyrus		R	38	28	-14	7.89
	Midbrain (substantia nigra/periaqueductal gray)		L	-6	-28	-6	6.66
	Midbrain (substantia nigra/periaqueductal gray)		R	6	-28	-6	6.95
	Middle occipital gyrus	658	L	-46	-76	6	17.17
	Middle occipital gyrus		R	46	-76	2	15.09
	Dorsomedial prefrontal cortex	475	L	-2	48	34	9.52
	Precuneus	245	R	2	-60	30	7.15
	Ventromedial prefrontal cortex	110	R	2	40	-22	8.06
	Cerebellum	60	L	-18	-76	-38	6.24
Pleasant > Neutral	Amygdala	3854	L	-18	-8	-14	7.40
	Amygdala		R	22	-4	-14	8.15
	Hippocampus		L	-26	-24	-10	4.80
	Hippocampus		R	26	-28	-6	4.95
	Fusiform gyrus		L	-42	-52	-22	10.69
	Fusiform gyrus		R	42	-48	-18	13.09
	Middle occipital gyrus		L	-46	-80	6	15.83
	Middle occipital gyrus		R	50	-76	-2	16.81
	Middle/superior temporal gyrus		L	-46	-68	6	9.77
	Middle/superior temporal gyrus		R	50	-64	6	14.97
	Thalamus		L	-6	-12	6	3.63
	Precuneus		L	-2	-60	38	9.42
	Dorsomedial prefrontal cortex	582	L	-6	56	6	7.31
	Dorsomedial prefrontal cortex		R	6	56	10	8.03
	Ventromedial prefrontal cortex		R	2	36	-22	6.62
	Pre-supplmentary motor area	67	R	10	8	66	5.40
	Precentral gyrus	44	R	50	-4	50	6.21

Table S4. Conservative versus Liberal groups in the [Disgusting > Neutral] contrast (Figure S2D). Height threshold, t = 2.67; extent threshold = 10 voxels (4x4x4 mm^3). L: Left, R: Right. Activation with a * mark survives the whole-brain FWE correction at the cluster level ($p < 0.05$, $k \geq 109$).

Contrast	Region	Cluster size	Side	MNI coordinates x	y	z	Peak T-score
Conservative > Liberal	Globus pallidus*	591	L	-14	-4	6	4.72
	Globus pallidus*		R	18	-8	-6	4.64
	Caudate*		L	-14	8	10	4.05
	Caudate*		R	18	8	14	3.70
	Putamen*		L	-18	8	10	4.31
	Putamen*		R	22	0	-2	3.12
	Amygdala/hippocampus*		L	-30	-4	-14	3.20
	Amygdala/hippocampus*		R	22	-8	-10	3.66
	Thalamus*		L	-10	-16	14	3.97
	Thalamus*		R	18	-12	2	4.17
	Middle/superior temporal gyrus*	163	L	-46	-68	14	3.99
	Fusiform gyrus*		L	-42	-52	-14	3.17
	Pre-supplementary motor area	79	L	-6	12	66	3.75
	Cerebellum	52	L	-22	-84	-42	3.79
	Cerebellum		R	22	-76	-42	4.18
	Anterior cingulate cortex	29	R	10	44	10	3.48
	Dorsolateral prefrontal cortex	26	L	-46	4	50	4.09
	Ventromedial prefrontal cortex	12	R	6	36	-22	3.03
	Inferior frontal gyrus	11	R	50	28	6	3.08
	Posterior cingulate cortex	10	L	-6	-48	30	3.11

Supplemental Experimental Procedures

Inclusion/exclusion criteria for participants and demographic data

Participants were required to be at least 18 years of age and to meet standard health and safety requirements for the MRI experiment. Additional twelve participants were excluded from all the analyses: 5 participants had more than 3mm of maximum movement; 2 participants had aberrant brain structure (e.g., extremely large ventricles); 1 participant fell asleep during the fMRI experiment and had extremely long reaction time (RT) for button-press trials; 2 participants showed no or little activity in the visual cortex for the disgust > neutral contrast in the first level analysis (threshold $p < 0.05$, $k = 10$, uncorrected); 2 participants had signal dropout. Participants gave informed consent in accordance with the Institutional Review Board at Virginia Tech Carilion Research Institute (VTCRI), VA, USA. After filling out a screening form and a written consent, participants were given written and verbal instructions on the task. Participants were divided into three groups based on their political ideology score. Neither age ($F(2,80)=2.331$, $p=0.104$) nor sex ($\chi^2(2)=2.597$, $p=0.273$) was significantly different across groups. When we tested just Liberal and Conservative groups, the Liberal group was marginally older than the Conservative group ($t(54)=1.857$, $p=0.069$). There were more males in the Liberal group compared to the Conservative group, but the group difference on age was not significant ($\chi^2(1)=1.786$, $p=0.181$).

Subconditions of emotional pictures used in the fMRI task

Each emotional condition, except the neutral condition, has two subconditions: 9 core/contamination (e.g., a dirty toilet) and 11 animal reminder (e.g., mutilated body) pictures in the disgusting condition, 10 actual threat (threatening objects aimed at another individual) and 10 no actual threat (e.g., a knife or a barking dog) pictures in the threatening condition, and 9 social pleasure (e.g., babies playing together) and 11 nonsocial pleasure (e.g., beautiful scenery) pictures in the pleasant condition. Also see Appendix S1 for more details.

Behavioral rating session

After the fMRI session, participants evaluated how disgusting, threatening, or pleasant were each of the 80 pictures according to a 9-point Likert scale. For the pleasant rating, 1 indicates "extremely unpleasant", 5 indicates "neutral" and 9 indicates "extremely pleasant". For the disgusting and threatening ratings, 1 indicates "not at all", 5 indicates "moderately", and 9 indicates "extremely". Ratings for each condition of emotion were elicited in separate blocks. The order of blocks was counterbalanced across participants. Importantly, participants did not know they had to evaluate the pictures when participating in the fMRI session. The rating session took approximately 20-30 minutes. **Table S2** summarizes the behavioral ratings of all pictures in each rating category, which are consistent with previously known IAPS valences and picture conditions.

Survey session

After the rating session, participants filled out three computer-based questionnaires. The first questionnaire (Appendix S2A) asked their political attitudes and involvement as well as their religiousness. Participants' political orientations were based on three components: their ideological position (item#1), partisan affiliation (item#2), and their policy preferences on the Wilson-Patterson questionnaire (Wilson-Patterson Issue Battery). Each component was first converted to a normalized score such that its minimum score is 0 and maximum score is 1 (e.g., if a participant selects "2. moderate, leaning liberal" on item#1, its normalized score is (2-1)/4 = 0.25). The political orientation (attitude) score was created by equally weighting the three components (min = 0 and max = 1). We also asked for whom they planned to vote (until November 6, 2012) or for whom they actually voted (after November 6, 2012). The second questionnaire (State-Trait Anxiety Inventory) assessed the state and trait anxiety [S2], and the third questionnaire (Disgust Scale-Revised) assessed the individual differences in disgust sensitivity (Appendix S2B) [S3, S4]. It took approximately 10-20 minutes to complete the survey session. Participants were debriefed and thanked for their participation after the survey session.

Relationship of political attitudes with demographic and other variables

We examined whether demographic data (age, sex) or other study variables (political interest, religiousness, trait anxiety, state anxiety, disgust sensitivity) are related to political attitudes. When we examined their relationships by simple correlations across all 83 participants, religiousness ($r(81) = 0.284$, $p = 0.009$), age ($r(81) = -0.232$, $p = 0.035$), and sex ($r(81) = 0.221$, $p = 0.045$) were significantly correlated with political attitudes at $p < 0.05$ (uncorrected). However, this type of uncorrected correlation analysis with multiple predictors is more likely to produce "false alarms". Thus, we next conducted a hierarchical Bayesian multiple regression analysis [S5, S6], which assigns a higher-level distribution across the regression coefficients of multiple predictors. Specifically, regression coefficients are coming from a t-distribution and its parameters (mean, scale, and df) are estimated from data, modeling a typical scenario of regression analyses (i.e., regression coefficients of many predictors are nearly zero and only a few predictors have non-zero regression coefficients). Because of the hierarchical structure, we are much less likely to have falsely significant results. See [S5, S6] for more details and the code for hierarchical Bayesian multiple regression ("MultiLinRegressHyperJags.R") is available at John K. Kruschke's website (*http://www.indiana.edu/%7Ekruschke/DoingBayesianDataAnalysis/Programs/).*

The results with the hierarchical Bayesian multiple regression showed that neither age nor sex was credibly associated with political attitude. However, political attitude was associated with religiousness (mean beta coefficient = 0.07, 95% highest density interval (HDI) of its posterior distribution = [0.019, 0.117]). Here, we used a heuristic Bayesian decision rule [S6]: a predictor is credibly associated with our independent variable if the 95% HDI of the predictor excludes zero. Note that the relationship between religiousness and conservatism has been well documented in previous literature (e.g., [S7, S8]).

We added age and sex as covariates in our standard General Linear Model (GLM) imaging analyses. For elastic net analysis (see below for the details), we used only fMRI data as predictors to test how accurately we can predict political attitudes with neural data alone. When we add age and sex as additional regressors to neural data for the elastic net analysis, both age and sex fail to survive at all (survival rate = 0.000), which means all the results using the elastic net approach remain unchanged even when we add the demographic variables.

Test-retest reliability of political attitudes

For a subset of participants (n=32), we re-assessed their political attitudes after some interval (mean = 171.7 days, SD = 142.1 days). The test-retest correlation coefficient was very high (Pearson correlation: $r(30) = 0.952$, $p < 2.2E-16$; Robust correlation: $r = 0.986$, $p < 2.0E-16$). The interval between the first and the second assessments was not significantly related to the difference of political attitudes between two assessments ($r(30) = 0.080$, $p = 0.662$).

fMRI data analysis

Image acquisition and preprocessing analysis: The anatomical and functional imaging sessions were conducted on a 3.0 tesla Siemens Magnetom Trio scanner at VTCRI. High-resolution T1-weighted scans (1x1x1 mm^3) were acquired using an MP-RAGE sequence (Siemens). Functional images were collected using echo-planar imaging with repetition time (TR) = 2,000ms and echo time (TE) = 25ms. 37 4mm slices (voxel size = 3.4 x 3.4 x 4mm^3) were acquired after angled 30 degrees with respect to the anteroposterior commissural line [S9].

Functional data were first spike-corrected to reduce the impact of artifacts using AFNI's 3dDespike (http://afni.nimh.nih.gov/afni). Data were subsequently preprocessed with SPM8 (http://www.fil.ion.ucl.ac.uk/spm/software/spm8/) for slice-timing correction using the first slice as the reference slice, motion correction, coregistration, gray/white matter segmentation, normalization to the Montreal Neurological Institute (MNI) template, and spatial smoothing using an 8mm full-width/half-maximum Gaussian kernel. Postprocessing voxels were 4 x 4 x 4 mm^3.

General linear model for standard fMRI analyses: All standard imaging analyses were conducted using the General Linear Model (GLM) implemented in SPM8. All first level analyses included a temporal high-pass filter (128s) and order 1 temporal autocorrelation (AR(1)) was assumed. For the main effects of emotional processing, the first level GLM was specified for each participant. The onsets for each picture subcondition (core/contamination disgust, animal reminder disgust, actual threat, no actual threat, social pleasure, nonsocial pleasure) and fixation crosses were convolved with a canonical hemodynamic response function (with time and dispersion derivatives) using a delta function of zero duration (or reaction time (RT) for fixation crosses) in SPM8. Initially, we probed to examine the separate neural correlates of emotional subconditions, but we collapsed them within each emotional condition to simplify the analysis. Six head motion parameters were also included in the first level GLM as covariates. The

random-effects analyses were conducted using the contrast images from the first level GLM. We separately examined the maps of [Disgusting - Neutral], [Threatening - Neutral], and [Pleasant - Neutral] contrasts (see **Figure S2** for family-wise error (FWE) cluster-level corrected maps and also **Table S3**).

To examine the neural correlates of political attitudes, for each contrast of [Disgusting - Neutral], [Threatening - Neutral], and [Pleasant - Neutral], we conducted independent t-tests comparing the Liberal and Conservative groups at the 2nd level GLM. Age and sex were added in the 2nd level analysis as covariates. To report clusters of voxels significantly related to political attitudes, we used a height threshold of $p < 0.005$ with extent threshold of $k \geq 10$ voxels or FWE cluster-level correction ($k \geq 107$) (**Figure S2D**), which was implemented in *CorrClusTh* by Thomas Nichols (http://go.warwick.ac.uk/tenichols/scripts/spm/spm8/CorrClusTh.m).

fMRI results on individual differences using standard GLM analysis: The contrasts with threatening or pleasant pictures revealed no regions surviving multiple corrections. However, in the [Disgusting > Neutral] contrast, the Conservative group showed greater activity than the Liberal group in several regions (**Figure S2D** and **Table S4**), including striatum, globus pallidus, thalamus, periaqueductal gray, substantia nigra, hippocampus, amygdala, dorsolateral prefrontal cortex, anterior cingulate cortex, pre-supplementary motor area, fusiform gyrus, and middle/superior temporal gyrus. No regions survived correction for multiple comparisons for the [Liberal group > Conservative group] comparison.

Preparation of data for penalized logistic regression analysis: For penalized logistic regression analysis, we prepared the data as described below (**Figure S3A**). First, we extracted a map of the [Disgust > Neutral] contrast for each participant. Then, we applied an a priori mask, which was generated from the Neurosynth website (www.neurosynth.org) [S10] containing a total of 7,471 voxels (voxel size = 4 x 4 x 4 mm^3); that is, we only used voxels inside the mask as predictors in the following analysis. We obtained the union of meta-analytic (positively correlated and both forward and reverse inference) maps of “Emotion” and “Attention” terms, thresholded at $q < 0.05$ False Discovery Rate corrected. Then, we generated a matrix (matrix size = number of participants x number of voxels) by reshaping each participant’s contrast maps (three-dimensional data) into one-dimensional vectors for penalized regression analysis.

Cross-validated penalized logistic regression analysis: We used the elastic net [S11], a penalized (logistic) regression technique, to make cross-validated predictions of political group membership and select voxels important for such predictions. Here, we first briefly explained the penalization technique and its relationship to the ordinary regression analysis. We used the continuous regression analysis as an example because of its relative easiness to understand. For more detailed reviews, see [S11-S14].

In the usual multiple linear regression analysis, we have N observations (participants), p number of regressors (or predictors, $x_i = (x_{i1}, x_{i2}, \ldots, x_{ip})$), and N number of responses (y_i). For

example, p is the number of voxels (p = 7,471), x_i are subjects' beta coefficients of each voxel (x_i = a matrix whose size is N x p), and y_i is an individual difference measure (e.g., y_i are political ideology scores, a vector of length N). In ordinary multiple linear regression, the objective function ($f(\theta)$) to minimize is: $f(\theta)=\sum_{i=1}^{N}(y_i-\beta_0-x_i^T\cdot\beta)^2$ where β_0 is the intercept and β are regression coefficients (= a vector of length p). When there are too many predictors (i.e., $p \gg N$), it is well known that the ordinary multiple linear regression performs poorly in prediction accuracy (generalization) and interpretation [S11]. To improve the performance, several penalization methods have been proposed, which impose an L1 penalty, an L2 penalty, or both. The L1 penalty refers to when $\|\beta\|_1=\sum_{j=1}^{p}|\beta_j|$ (= sum of absolute values of coefficients) is constrained and the L2 penalty refers to when $\|\beta\|_2^2=\sum_{j=1}^{p}\beta_j^{\,2}$ (= sum of squared values of coefficients) is constrained when estimating regression coefficients. The elastic net that we used in our work employs both L1 and L2 penalization. As suggested by previous works [S15-S18], the elastic net has several advantages for fMRI data compared to other machine learning techniques such as the conventional Support Vector Machine (SVM) [S19] or the least absolute shrinkage and selection operator (LASSO) [S12]. First, like other penalized regression techniques (c.f., ridge regression), it does continuous shrinkage and automatic selection of predictors (i.e., the regression coefficients of unimportant predictors shrink to zero) due to L1 penalization. Thus, the elastic net automatically selects voxels that are critical for out-of-sample prediction accuracy (i.e., automatic variable selection), which increases the interpretability of the findings. Second, the elastic net enjoys a grouping effect, which clusters highly correlated predictors into a set of groups due to L2 penalization. Also, in fMRI studies, the number of predictors (p) is much greater than the number of subjects (N) (i.e., $p \gg N$) where the conventional LASSO is an inadequate choice (c.f., [S20, S21]).

The elastic net regression has two tuning parameters (α and λ), which are selected by cross validation (CV) over two-dimensional surface (**Figure 2D**). In the elastic net regression, the objective function to minimize for the Gaussian family is: $f(\theta)+\lambda\left[(1-\alpha)\|\beta\|_2^2/2+\alpha\|\beta\|_1\right]$ where the second term is called the *elastic net penalty* and $f(\theta)$ is the objective function for the ordinary multiple linear regression: $f(\theta)=\frac{1}{2N}\sum_{i=1}^{N}(y_i-\beta_0-x_i^T\beta)^2$. The tuning parameter λ ($\lambda \geq 0$) governs the overall complexity of the model (λ values close to zero approach the full (non-penalized) regression model), and α ($0 \leq \alpha \leq 1$) is the elastic-net mixing parameter, which compromises between the ridge (α = 0) and the LASSO (α = 1). For a penalized binomial logistic regression [S13], the objective function to minimize has the same form and the elastic net penalty: $f(\theta)+\lambda\left[(1-\alpha)\|\beta\|_2^2/2+\alpha\|\beta\|_1\right]$, but with different y_i (e.g., y_i = 0 for the Liberal

group and 1 for the Conservative group) and $f(\theta)$: $f(\theta) = -\left[\frac{1}{N} \sum_{i=1}^{N} y_i \cdot (\beta_0 + x_i^T \beta) - \log(1 + \exp(\beta_0 + x_i^T \beta)) \right]$.

For the selection of tuning parameters and estimation of the elastic net penalized logistic regression model, we used the *glmnet* package for Matlab (http://www.stanford.edu/~hastie/glmnet_matlab/) and R [S14]. Regressors were standardized prior to fitting the model, which is a standard routine for a penalized logistic regression analysis. We also used a built-in Matlab function (*cvglmnet.m*) in the glmnet package for CV and another built-in function (*glmnetPredict.m*) for making predictions. Two authors (W.-Y.A. and T.L.) independently tested the penalized logistic regression procedure and verified the findings.

For the selection of tuning parameters, we first estimated the elastic-net mixing parameter (α) and then the overall complexity parameter (λ). For the estimation of α, we conducted the following procedure over 1,000 grids of α (α = 0.001, 0.002, 0.003, … , 0.998, 0.999, 1.000):

1.1. At each α value, first fits the elastic net (LASSO if α = 1) model paths to get the λ sequence.
1.2. Divide data randomly into 10 partitions.
  1.2.1. Train the model based on 90% of the data (9 partitions = 50 subjects' data) and compute the minimum binomial deviance (at the λ value that minimizes the binomial deviance) on the 10% of the data (1 partition = 6 subjects' data).
  1.2.2. Repeat step 1.2.1 10 times and compute the average binomial deviance over the 10 repetitions. Calling *cvglmnet.m* (in Matlab) command runs both steps 1.2.1 and 1.2.2.
1.3. CV performance can vary slightly depending on how the data set is divided into K (=10) partitions. To get more reliable estimates, repeat the whole steps 1.1 and 1.2 twenty times and compute the average binomial deviance over the 20 repetitions, which is in fact the average binomial deviance over 200 (=10*20) iterations at each α.

As seen in **Figure 2D** (red dashed line in step 2), α value of 0.026 minimized the average binomial deviance. Setting α to 0.026, we estimated the second tuning parameter, overall complexity λ. While α is set to 0.026, we first estimated the trace plot of coefficient values for each λ value (**Figure 2D**, left figure in step 3). Then, we repeated the following procedure 1,000 times:

2.1. Run a 10-fold CV (similar to steps 1.2.1 and 1.2.2 but with setting α to 0.026) to find the λ value that minimizes the binomial deviance and one that is +1 S.E. from the minimum λ value (**Figure 2D**, two dashed lines in the right figure in step 3. The numbers on top of x-axis are the number of survived regressors for each λ value).

2.2. Extract β coefficients of all regressors (voxels) using the λ value that is +1 S.E. from the minimum (i.e., +1 S.E. λ). Using +1 S.E. λ is a heuristic strategy to produce a less complex and more conservative model [S22]. The β coefficients of many regressors would shrink to zero. Record whether each regressor (voxel) survived (β coefficient is non-zero) or not (β coefficient is zero) in the current iteration.

2.3. Make predictions (fitted probabilities that range from 0 to 1) for the political group based on the β coefficients of the penalized logistic regression model. Then compute the area under the curve (AUC) of the receiver operating characteristic (ROC) curve.

Calculating the "survival rate" of voxels: After finishing 1,000 CVs, we generated a matrix that indicates whether each voxel survived (1 if its β coefficient is non-zero, 0 if its β coefficient is zero) CV on each iteration (= a matrix size of 1,000 x 7,471, **Figure 2D**, step 4). We defined the 'survival rate' as the proportion of each voxel surviving CV over 1,000 iterations (i.e., 0 indicates that the voxel survived 0 times out of 1,000 iterations and 1 indicates that it survived all 1,000 iterations). Survival rate is closely related to regression coefficients (**Figure S3B**). We also generated another matrix that contained the signs of mean β coefficients over 1,000 iterations. To separately visualize voxels that predict either the Conservative ($\beta > 0$) or the Liberal group ($\beta < 0$), we projected the [survival rate x the sign of the mean β coefficient] of each voxel back into the brain (voxel) space (**Figure 3A**).

Checking the validity of the elastic net model: To make sure our elastic net procedure is unbiased toward finding group differences, we tested if the penalized logistic regression can predict permuted outcome variable [S20]. In other words, we permuted (randomly sampled) our outcome variable (political ideology group membership), and let the elastic net model predict the permuted variable. If our elastic net model is valid and unbiased, AUC for permuted data should be 0.5 (at chance level). Indeed, mean AUC of the penalized logistic regression model was 0.50 over 1,000 iterations, which confirms that our model is unbiased.

Supplementary Information for **Figure 3B**, histogram of CV ROC AUCs: In order to more fully demonstrate the out-of-sample performance of the penalized logistic regression analysis, we did the following. First, following the main approach in the text, we categorized participants' political attitudes by terciles, and then restricted our analysis to the low (Liberal group) and high (Conservative group) terciles. We then repeated 1,000 times 1) Run the CV procedure implemented by *cvglmnet.m* and find the value of λ and the beta coefficients of predictors that minimizes binomial deviance. Here we use 5-fold cross validation to have enough sample size for computing AUC. We set the mixing parameter (α) to 0.026; 2) for each iteration at the minimum λ, and for each of the five folds, use the stored fits on the data not including the fold (80% of the data) from the iteration's CV model to predict the class probabilities for the data in the fold. Then use the MATLAB *perfcurve* function to compute the receiver operating

characteristic curve (ROC), and the area under this curve. 5 AUCs are computed for each iteration, giving a total of 5,000 data points for the histogram.

Penalized linear regression analysis: **Figure 3A** shows the voxels critical for cross-validated classification accuracy for predicting Liberal and Conservative group membership. In **Figure S4**, we used panelized linear regression, predicting political attitudes in a continuous fashion across all participants (n=83). The procedure is identical to cross-validated penalized logistic regression analysis reported above except the following: First, we used penalized linear regression, which has a different objective function to minimize for the Gaussian family as described above. Second, we re-estimated α (mixing parameter) value that is optimized for penalized linear regression across all participants. The estimated α value optimized for penalized linear regression was 0.222, but we report the results with both of the α values (for **Figure S4A**, we used 0.026, which was optimized for penalized logistic regression and for **Figure S4B**, we used 0.222). Third, we used a correlation coefficient as a measure of predictive accuracy. In each of 1,000 iterations, we first computed each participant's predicted political attitude. After 1,000 iterations, we computed each participant's mean predicted political attitudes over 1,000 iterations. Then, we calculated the correlation coefficient between actual political attitudes and the averaged predicted political attitudes. As seen in **Figures 3A, S4A, and S4B**, regions predicting continuous political attitudes substantially overlap with regions predicting Liberal and Conservative group membership. Right panels in **Figures S4A and S4B** show the correlations between actual political attitudes and predicted political attitudes from the BOLD data, with each of the two α values.

Out-of-sample prediction using the split half approach: **Figures S4A and S4B** show the voxels critical for predicting individual differences in political ideology using data from all the participants (n=83). Alternatively, we tested if our machine learning procedure can make accurate predictions on the half of the data (test set) when the model is trained on the other half of the data (training set). To test it, participants were first sorted based on their political ideology scores (i.e., sorted participant#1 is the most liberal participant, and sorted participant#83 is the most conservative participant). Then, participants #1, #3, #5, …, #81, #83 were used as the test set, and participants #2, #4, #6, …, #80, #82 were used as the training set. We used this procedure to ensure that training and test sets are widely and evenly distributed across the whole spectrum of political ideology scores.

We used the identical procedure reported for the penalized linear regression analysis except the following: First, we only used the training set (n=41) to fit the machine learning (elastic net) model (i.e., estimation of λ and the beta coefficients of predictors). Second, the predictions were made only on the test set (n=42) based on the model estimated only with the training set. Third, we used the minimum λ (instead of +1 S.E. λ) when making the predictions on the test set to achieve best accuracy. We found that many regions (e.g., striatum, thalamus, inferior parietal lobule) overlap substantially with what we found from using all participants

(**Figure S4**). The out-of-sample prediction performance (correlation coefficient between actual and predicted political attitudes) on the test set was 0.52 (**Figure 3B (figure inset)**, p = 0.0004, robust r = 0.44, robust p = 0.0024). The correlation value is lower than when we computed cross-validated predictive accuracy across all participants (e.g., **Figures S4A and S4B**), but we should keep in mind that we used only 41 participant' data for the training of the elastic net model, and performance of a machine learning model crucially depends on the amount of data we can use for the training. Also note that model performance can vary depending on how we divide the data into test and training sets. We found that the out-of-sample prediction performance (correlation coefficient) varied between 0.40 and 0.52 when we divided the data in different ways (e.g., using the one third (33%) of the data as the test set and two thirds (67%) of the data as the training set). The correlation coefficients were always highly significant ($p < 0.003$).

Single-stimulus fMRI analysis: The elastic net analysis described above used the whole brain [Disgusting > Neutral] contrast map (the number of predictors = 7,741), collected approximately for 20 minutes. Here, we used the BOLD time series data of the first disgusting stimulus only (20 seconds), which was spatial averaged from all the (+) voxels (**Figure 3A** red-to-yellow regions).

Figure S3C illustrates how we prepared the data for the single-stimulus fMRI analysis. In each participant, we extracted raw BOLD time series during the whole experiment that are spatially averaged within each of two types of regions (i.e., (+) voxels or (-) voxels). Each voxel was equally weighted in each mask. After applying a high pass filter and linearly interpolating time series data every 1s, we captured the data for the period 2 seconds prior to the first disgusting stimulus presentation to 16 seconds post-stimulus onset (a total of 20 seconds) every TR (= 2s). Thus, each subject had 11 time points or predictors. BOLD signals were converted to percentage signal change after correcting the baseline activity using the mean baseline value (2 seconds prior to 0 seconds post stimulus). Then, we combined all participants' data into a single matrix (matrix size = number of participants x number of time points) (**Figure S3C, right side**). When conducting the single-trial fMRI analysis on each region of interest (ROI), we extracted averaged raw time series from each region only (**Figures 4C**).

The remaining steps for penalized logistic regression analysis remained the same: We estimated a tuning parameter (λ) and the beta coefficients of predictors using CV and computed the survival rate and the AUC of the ROC curve. We used the α value (= 0.026) estimated from the full-data analysis, but for the single-stimulus fMRI analysis when $p < n$, AUC values did not depend much on α value.

Single-stimulus fMRI analysis on independent ROIs: We used two ROIs (amygdala and posterior insula) from a previous study [S1] for a single-stimulus analysis. To our knowledge, Schreiber et al. (2013) is the only study that found neural correlates of political orientation using non-political stimuli. For each ROI, we first generated a 8mm sphere centered on its peak coordinates (Amygdala=[20, -6, -10], posterior insula=[-36, -40, 18] in Talairach coordinates, which were converted to MNI coordinates using http://noodle.med.yale.edu/~papad/mni2tal/).

Then we conducted single-stimulus analysis as we described above. We found that mean AUC of the amygdala ROI (**Figure S4C**) was 0.565 (SD = 0.098) when we used +1 S.E. λ. With minimum λ that maximizes predictive accuracy, mean AUC was 0.745 (SD = 0.025). AUC of posterior insula was 0.500 (= at chance level) with either +1 S.E. or minimum λ, which is consistent with what we found in our own peak ROI of posterior insula. Although AUC with the amygdala ROI from [S1] is worse than AUC with our peak amygdala/hippocampus ROI (mean AUC = 0.721 with +1 S.E. λ), we should keep in mind that [S1] used a risky decision-making paradigm, which is very different from our passive-viewing paradigm using emotionally evocative images.

<u>Group comparisons on the characteristics of the first disgusting pictures:</u> We checked IAPS valence ratings, IAPS arousal ratings, and onsets of the first disgusting pictures in each political group (**Table S1**). None of IAPS ratings were significantly different between Liberal and Conservative groups (IAPS valence, t(54) = 0.336, p = 0.738; IAPS arousal, t(54) = 0.891, p = 0.377). The conservative group had marginally longer onset time than the Liberal group (t(54) = 1.891, p = 0.064). Group difference on subconditions of the first disgusting stimuli was not significant between the Liberal and Conservative groups ($\chi^2(1)=0.072$, p=0.789).

Supplemental References

S1. Schreiber, D., Fonzo, G., Simmons, A. N., Dawes, C. T., Flagan, T., Fowler, J. H., and Paulus, M. P. (2013). Red Brain, Blue Brain: Evaluative Processes Differ in Democrats and Republicans. PloS one *8*, e52970. Available at: http://www.plosone.org/article/info%3Adoi%2F10.1371%2Fjournal.pone.0052970#s5.

S2. Spielberger, C. D., and Gorsuch, R. L. (1983). Manual for the State-trait anxiety inventory (form Y) ("self-evaluation questionnaire").

S3. Haidt, J., McCauley, C., and Rozin, P. (1994). Individual differences in sensitivity to disgust: A scale sampling seven domains of disgust elicitors. Pers. Individ. Dif. *16*, 701–713.

S4. Olatunji, B. O., Williams, N. L., Tolin, D. F., Abramowitz, J. S., Sawchuk, C. N., Lohr, J. M., and Elwood, L. S. (2007). The Disgust Scale: Item analysis, factor structure, and suggestions for refinement. Psychol. Assessment *19*, 281–297.

S5. Ahn, W.-Y., Vasilev, G., Lee, S.-H., Busemeyer, J. R., Kruschke, J. K., Bechara, A., and Vassileva, J. (2014). Decision-making in stimulant and opiate addicts in protracted abstinence: evidence from computational modeling with pure users. Front Decision Neurosci *5*.

S6. Kruschke, J. K. (2011). Doing Bayesian Data Analysis (Burlington, MA: Elsevier).

S7. Brandt, M. J., and Reyna, C. (2010). The Role of Prejudice and the Need for Closure in Religious Fundamentalism. Pers. Soc. Psychol. B. *36*, 715–725.

S8. Duriez, B., Luyten, P., and Snauwaert, B. (2002). The importance of religiosity and values in predicting political attitudes: Evidence for the continuing importance of religiosity in Flanders (Belgium). Mental Health *5*, 35–54.

S9. Deichmann, R., Gottfried, J. A., Hutton, C., and Turner, R. (2003). Optimized EPI for fMRI studies of the orbitofrontal cortex.

S10. Yarkoni, T., Poldrack, R. A., Nichols, T. E., Van Essen, D. C., and Wager, T. D. (2011). Large-scale automated synthesis of human functional neuroimaging data. Nat. Methods *8*, 665–670.

S11. Zou, H., and Hastie, T. (2005). Regularization and variable selection via the elastic net. J. R. Stat. Soc. Ser. B. *67*, 301–320.

S12. Tibshirani, R. (1996). Regression shrinkage and selection via the lasso. J. R. Stat. Soc. Ser. B Stat. Methodol., 267–288.

S13. Simon, N., Friedman, J., and Hastie, T. (2011). Regularization paths for Cox's proportional hazards model via coordinate descent. J. Stat. Softw.

S14. Hastie, T. (2010). Regularization paths for generalized linear models via coordinate descent. *33*, 1. Available at: http://www.ncbi.nlm.nih.gov/pmc/articles/PMC2929880/.

S15. Ryali, S., Chen, T., Supekar, K., and Menon, V. (2012). Estimation of functional connectivity in fMRI data using stability selection-based sparse partial correlation with elastic net penalty. *59*, 3852–3861.

S16. Ryali, S., Supekar, K., Abrams, D. A., and Menon, V. (2010). Sparse logistic regression for whole-brain classification of fMRI data. *51*, 752–764.

S17. Smith, A., Bernheim, B. D., Camerer, C. F., and Rangel, A. (2014). Neural Activity Reveals Preferences Without Choices. Am. Econ. J.-Microecon. *6*, 1–36.

S18. Whelan, R., Watts, R., Orr, C. A., Althoff, R. R., Artiges, E., Banaschewski, T., Barker, G. J., Bokde, A. L. W., Büchel, C., Carvalho, F. M., et al. (2014). Neuropsychosocial profiles of current and future adolescent alcohol misusers. Nature *512*, 185–189.

S19. Cortes, C., and Vapnik, V. (1995). Support-vector networks. Mach. Learn.

S20. Wager, T. D., Atlas, L. Y., Leotti, L. A., and Rilling, J. K. (2011). Predicting Individual Differences in Placebo Analgesia: Contributions of Brain Activity during Anticipation and Pain Experience. J. Neurosci. *31*, 439–452.

S21. Wager, T. D., Atlas, L. Y., Lindquist, M. A., Roy, M., Woo, C.-W., and Kross, E. (2013). An fMRI-based neurologic signature of physical pain. N. Engl. J. Med. *368*, 1388–1397.

S22. Hastie, T. J., Tibshirani, R. J., and Friedman, J. J. H. (2009). The elements of statistical learning (Springer).

Appendix S1. IAPS valence and arousal ratings of stimuli in each (sub)condition

IAPS picture number	Condition	Subcondition	Valence	Arousal	Description
1111	disgusting	core/contamination	3.25	5.20	Snakes
1274	disgusting	core/contamination	3.17	5.39	Roaches
7360	disgusting	core/contamination	3.59	5.11	FliesOnPie
7380	disgusting	core/contamination	2.46	5.88	RoachOnPizza
9008	disgusting	core/contamination	3.47	4.45	Needle
9290	disgusting	core/contamination	2.88	4.40	Garbage
9300	disgusting	core/contamination	2.26	6.00	Dirty
9320	disgusting	core/contamination	2.65	4.93	Vomit
9390	disgusting	core/contamination	3.67	4.14	Dishes
9570	disgusting	animal reminder	1.68	6.14	Dog
3010	disgusting	animal reminder	1.79	7.26	Mutilation
3030	disgusting	animal reminder	1.91	6.76	Mutilation
3060	disgusting	animal reminder	1.79	7.12	Mutilation
3102	disgusting	animal reminder	1.40	6.58	Burn victim
3130	disgusting	animal reminder	1.58	6.97	Mutilation
3150	disgusting	animal reminder	2.26	6.55	Mutilation
3170	disgusting	animal reminder	1.46	7.21	Baby tumor
3250	disgusting	animal reminder	3.78	6.29	OpenChest
3266	disgusting	animal reminder	1.56	6.79	Injury
9405	disgusting	animal reminder	1.83	6.08	Sliced hand
2485	neutral	neutral	5.69	3.74	Man
2514	neutral	neutral	5.19	3.50	Woman
7004	neutral	neutral	5.04	2.00	Spoon
7010	neutral	neutral	4.94	1.76	Basket
7035	neutral	neutral	4.98	2.66	Mug
7095	neutral	neutral	5.99	4.21	Headlight
7100	neutral	neutral	5.24	2.89	Fire hydrant
7140	neutral	neutral	5.50	2.92	Bus
7150	neutral	neutral	4.72	2.61	Umbrella
7170	neutral	neutral	5.14	3.21	Light Bulb
7175	neutral	neutral	4.87	1.72	Lamp
7180	neutral	neutral	4.73	3.43	NeonBuilding
7190	neutral	neutral	5.55	3.84	Clock
7224	neutral	neutral	4.45	2.81	File cabinets
7233	neutral	neutral	5.09	2.77	Plate
7235	neutral	neutral	4.96	2.83	Chair

7491	neutral	neutral	4.82	2.39	Building
7500	neutral	neutral	5.33	3.26	Building
7550	neutral	neutral	5.27	3.95	Office
7595	neutral	neutral	4.55	3.77	Traffic
1440	pleasant	nonsocial	8.19	4.61	Seal
1460	pleasant	nonsocial	8.21	4.31	Kitten
1710	pleasant	nonsocial	8.34	5.41	Puppies
1750	pleasant	nonsocial	8.28	4.10	Bunnies
1920	pleasant	nonsocial	7.90	4.27	Porpoise
2040	pleasant	nonsocial	8.17	4.64	Baby
2070	pleasant	nonsocial	8.17	4.51	Baby
5760	pleasant	nonsocial	8.05	3.22	Nature
5849	pleasant	nonsocial	6.65	4.89	Flowers
7502	pleasant	nonsocial	7.75	5.91	Castle
8190	pleasant	nonsocial	8.10	6.28	Skier
2080	pleasant	social	8.09	4.70	Babies
2091	pleasant	social	7.68	4.51	Girls
2165	pleasant	social	7.63	4.55	Father
2360	pleasant	social	7.70	3.66	Family
2530	pleasant	social	7.80	3.99	Couple
2540	pleasant	social	7.63	3.97	Mother
2550	pleasant	social	7.77	4.68	Couple
5831	pleasant	social	7.63	4.43	Seagulls
8496	pleasant	social	7.58	5.79	Water slide
3500	threatening	actual threat	2.21	6.99	Attack
6350	threatening	actual threat	1.90	7.29	Attack
6550	threatening	actual threat	2.73	7.09	Attack
6821	threatening	actual threat	2.38	6.29	Gang
6838	threatening	actual threat	2.45	5.80	Police
9050	threatening	actual threat	2.43	6.36	Plane Crash
9622	threatening	actual threat	3.10	6.26	Jet
9910	threatening	actual threat	2.06	6.20	Auto accident
9911	threatening	actual threat	2.30	5.76	Car accident
9920	threatening	actual threat	2.50	5.76	Auto accident
1120	threatening	no actual threat	3.79	6.93	Snake
1302	threatening	no actual threat	4.21	6.00	Dog
2120	threatening	no actual threat	3.34	5.18	AngryFace
5972	threatening	no actual threat	3.85	6.34	Tornado
6200	threatening	no actual threat	3.20	5.82	AimedGun
6242	threatening	no actual threat	2.69	5.43	Gang
6260	threatening	no actual threat	2.44	6.93	AimedGun

6510	threatening	no actual threat	2.46	6.96	Attack
6830	threatening	no actual threat	2.82	6.21	Guns
9630	threatening	no actual threat	2.96	6.06	Bomb

Appendix S2: Survey Instrument

All participants completed the computer-based survey questionnaires. The questions and their coding scales for political ideology and disgust sensitivity are reproduced below.

A. Political (items 1-8) and religious (items 9 and 10) questions

1. Labels are often misleading, but in general do you consider yourself liberal, conservative, or something in between
    1. liberal
    2. moderate, leaning liberal
    3. moderate
    4. moderate, leaning conservative
    5. conservative

2. In general, do your consider yourself a Democrat, a Republican, or an Independent?
    1. strong Democrat
    2. weak Democrat
    3. Independent, leaning Democrat
    4. Independent
    5. Independent, leaning Republican
    6. weak Republican
    7. strong Republican
    8. other

3. (Until November 6, 2012) If the 2012 presidential election were held today, whom would you vote for? (After November 6, 2012) Who did you vote for President in 2012?
(answer order was randomized for each participant)
    1. Barack Obama
    2. Mitt Romney
    3. Other
    4. Not voting
    5. Not sure

4. How interested are you in politics and public affairs?
    1. very interested
    2. somewhat interested
    3. not very interested
    4. not at all interested

5. How strongly would you say you feel about political issues? Imagine a scale of feelings ranging from 1-10 with 1 representing no feelings at all and 10 representing intense feelings, and place yourself on this scale.

1. 1—no feelings at all on political issues
2. 2
3. 3
4. 4
5. 5—moderately strong feelings about political issues
6. 6
7. 7
8. 8
9. 9
10. 10—intense feelings about political issues

6. For each of the following, note whether it is something you have ever done.

a. attended a political meeting or rally
b. worked in a political campaign in any capacity (even for no pay)
c. contributed money to a political cause, party, or candidate
d. held any governmental office no matter how minor
e. communicated your thoughts or requests to a governmental official
    1. yes
    2. no

7. How would you describe your voting behavior?

1. I vote in nearly every election.
2. I vote in most elections.
3. I rarely vote.
4. I never vote.
5. I am ineligible to vote.

8. How often do you have discussions about politics with others?

1. very often
2. somewhat often
3. rarely
4. never

9. How often do you attend religious services?

a. Never or very rarely
b. Occasionally
c. Once per week

d. More than once per week

10. Do you regularly say grace before meals?
    a. Always
    b. Usually
    c. Occasionally
    d. Never

Wilson-Patterson Issue Battery

Here is a list of various topics. Please indicate how you feel about each topic.

1. strongly agree
2. agree
3. uncertain
4. disagree
5. strongly disagree

a. School prayer
b. Pacifism
c. Stop immigration
d. Death penalty
e. Government-arranged healthcare
f. Premarital sex
g. Gay marriage
h. Abortion rights
i. Evolution
j. Biblical truth
k. Increase welfare spending
l. Protect gun rights
m. Increase military spending
n. Government regulation of business
o. Small government
p. Foreign aide
q. Lower taxes
r. Stem cell research
s. Abstinence-only sex education
t. Allow torture of terrorism suspects

B. Disgust Scale – Revised (http://people.stern.nyu.edu/jhaidt/Dscale-R.doc)

Please indicate how much you agree with each of the following statements, or how true it is about you. Please write a number (0-4) to indicate your answer:

0 = Strongly disagree (very untrue about me)
1 = Mildly disagree (somewhat untrue about me)
2 = Neither agree nor disagree
3 = Mildly agree (somewhat true about me)
4 = Strongly agree (very true about me)

____1. I might be willing to try eating monkey meat, under some circumstances.
____2. It would bother me to be in a science class, and to see a human hand preserved in a jar.
____3. It bothers me to hear someone clear a throat full of mucous.
____4. I never let any part of my body touch the toilet seat in public restrooms.
____5. I would go out of my way to avoid walking through a graveyard.
____6. Seeing a cockroach in someone else's house doesn't bother me.
____7. It would bother me tremendously to touch a dead body.
____8. If I see someone vomit, it makes me sick to my stomach.
____9. I probably would not go to my favorite restaurant if I found out that the cook had a cold.
____10. It would not upset me at all to watch a person with a glass eye take the eye out of the socket.
____11. It would bother me to see a rat run across my path in a park.
____12. I would rather eat a piece of fruit than a piece of paper
____13. Even if I was hungry, I would not drink a bowl of my favorite soup if it had been stirred by a used but thoroughly washed flyswatter.
____14. It would bother me to sleep in a nice hotel room if I knew that a man had died of a heart attack in that room the night before.

How disgusting would you find each of the following experiences? Please write a number (0-4) to indicate your answer:

0 = Not disgusting at all
1 = Slightly disgusting
2 = Moderately disgusting
3 = Very disgusting
4 = Extremely disgusting

____15. You see maggots on a piece of meat in an outdoor garbage pail.
____16. You see a person eating an apple with a knife and fork
____17. While you are walking through a tunnel under a railroad track, you smell urine.
____18. You take a sip of soda, and then realize that you drank from the glass that an acquaintance of yours had been drinking from.
____19. Your friend's pet cat dies, and you have to pick up the dead body with your bare hands.
____20. You see someone put ketchup on vanilla ice cream, and eat it.

____21. You see a man with his intestines exposed after an accident.
____22. You discover that a friend of yours changes underwear only once a week.
____23. A friend offers you a piece of chocolate shaped like dog-doo.
____24. You accidentally touch the ashes of a person who has been cremated.
____25. You are about to drink a glass of milk when you smell that it is spoiled.
____26. As part of a sex education class, you are required to inflate a new unlubricated condom, using your mouth.
____27. You are walking barefoot on concrete, and you step on an earthworm.
